# Development of a Vinylated Cyclic Allene: A Fleeting Strained Diene for the Diels–Alder Reaction

**DOI:** 10.1002/anie.202510319

**Published:** 2025-06-26

**Authors:** Haruki Mizoguchi, Takumi Obata, Taiki Hirai, Manaka Komatsu, Akira Sakakura

**Affiliations:** ^1^ Graduate School of Environmental, Life, Natural Science and Technology Okayama University 3‐1‐1 Tsushima‐naka, Kita‐ku Okayama 700‐8530 Japan

**Keywords:** Activation strain model, Carbocycles, Diels–Alder reaction, Strained diene, Vinylated cyclic allene

## Abstract

Fleeting molecules possessing strained multiple bonds are important components in organic synthesis due to their ability to undergo various chemical reactions driven by the release of strain energy. Although the use of strained π‐bonds as 2π components, represented by dienophiles in Diels–Alder reactions, has been well studied, “the strained diene (4π component) approach” for molecular construction remains underexplored. Herein, we report the design of a vinyl cyclic allene (1‐vinyl‐1,2‐cyclohexadiene) as a highly reactive strained diene and the development of its Diels–Alder reactions. Experimental and computational studies of vinyl cyclic allenes revealed that this diene system undergoes cycloaddition with dienophiles regio‐ and stereoselectively under mild reaction conditions. These studies also provide insight into the reactivity and selectivity of the system. The strained diene approach enables the convergent construction of polycyclic molecules through bond disconnections distinct from conventional retrosynthetic analysis, thus offering an efficient strategy for the assembly of functional molecules.

## Graphical abstract

A strain‐promoted Diels–Alder reaction of the vinylated cyclic allene, a highly reactive strained all‐carbon diene, was developed. Activation of the Kobayashi‐type precursor with fluoride ion generated the vinyl cyclic allene, which was trapped by dienophiles to produce decalin derivatives under mild conditions. This method would provide an efficient route to synthesize natural product‐relevant multicyclic skeletons in a convergent fashion.

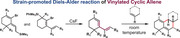

## Introduction

Strain has garnered significant attention from chemists due to its capacity to enhance the reactivity of bonds, thus facilitating challenging transformations without introducing electronic bias to the molecule.^[^ ^1^, ^2^, ^3^, ^4^, ^5^ ^]^ The release of strain energy serves as a powerful driving force in numerous bond‐forming and bond‐cleaving reactions. Consequently, strained molecular species are broadly used not only for reaction development and complex molecular syntheses^[^ ^6^, ^7^, ^8^, ^9^ ^]^ but also in interdisciplinary fields, such as polymer chemistry,^[^ ^10^ ^]^ material science,^[^ ^11^, ^12^ ^]^ and bioconjugation in chemical biology.^[^ ^13^, ^14^, ^15^, ^16^ ^]^

As the formation and cleavage of π‐bonds are involved in broad chemical reactions, the chemistry of short‐lifetime species that possess highly strained π‐bond, such as aryne,^[^ ^17^, ^18^ ^]^ cycloalkyne,^[^ ^19^ ^]^ cyclic allene,^[^ ^4^, ^20^, ^21^, ^22^, ^23^ ^]^ and their heterocyclic variants,^[^ ^24^, ^25^, ^26^ ^]^ has been extensively studied and made a variety of unique reactions possible with nonactivated reaction partners. Recently, there has been rapid growth in cycloaddition reactions^[^ ^27^, ^28^, ^29^, ^30^, ^31^, ^32^, ^33^, ^34^, ^35^, ^36^, ^37^, ^38^, ^39^ ^]^ and metal‐mediated reactions^[^ ^40^, ^41^, ^42^ ^]^ of carbocyclic and heterocyclic allenes due to the tremendous contributions of West, Garg, Houk, and other researchers, and applied in complex molecule synthesis.^[^ ^43^, ^44^, ^45^, ^46^ ^]^ Among these, the Diels–Alder reaction is one of the most well‐studied reactions in the chemistry of cyclic allenes. Although the Diels–Alder reaction often requires harsh reaction conditions to facilitate the coupling of electronically neutral dienes and dienophiles, the incorporation of strain in the π‐bond significantly lowers the activation energy barrier.^[^ ^47^ ^]^ Consequently, cycloadditions involving these strained π‐systems proceed under remarkably mild conditions.

**Figure 1:**
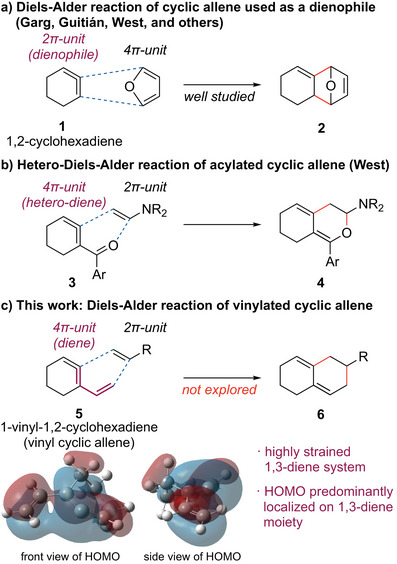
Diels–Alder reaction of a) cyclic allene, b) acylated cyclic allene, and c) vinylated cyclic allene (this work). Depicted HOMO was computed at HF/6‐31G level of theory.

As represented by the reaction of a cyclic allene (**1**, 1,2‐cyclohexadiene) with furan (Figure 1a), in most cases, cyclic allenes are utilized as dienophile components in the Diels–Alder reaction. The reaction proceeds at room temperature and produces fused carbocyclic frameworks **2**. An interesting exception to this reactivity was reported by West and coworkers utilizing acylated cyclic allene **3** as a hetero‐diene component (Figure 1b).^[^ ^48^ ^]^ They reported that ketone‐substituted cyclic allenes could react as both a dienophile and a hetero‐diene, and the reaction with electron‐rich alkene such as enamines produces dihydropyran‐containing cyclic systems **4** via hetero‐Diels–Alder reaction. On the other hand, to the best of our knowledge, utilization of the cyclic allene system as an all‐carbon 4π component in the Diels–Alder reaction has not been reported to date.^[^ ^49^ ^]^ We contemplated that the conjugation of a vinyl group to a cyclic allene framework (**5**, 1‐vinyl‐1,2‐cyclohexadiene) could provide a viable solution to this synthetic challenge (Figure 1c).

1,2‐Cyclohexadiene, a representative example of a cyclic allene, incorporates an allene moiety, which inherently possesses a linear structure within a 6‐membered ring. This structural arrangement imparts a high degree of strain energy (ca. 32 kcal mol^−1^) to the system.^[^ ^50^ ^]^ As mentioned above, this elevated strain energy correlates with enhanced reactivity. Therefore, we hypothesized that if a segment of this highly strained π bond could be incorporated as a component of a 1,3‐diene system, it would exhibit exceptionally high reactivity as an all‐carbon 4π‐component. As illustrated, the calculated highest occupied molecular orbital (HOMO) of the vinylated cyclic allene (vinyl cyclic allene) is predominantly localized on the 1,3‐diene moiety. Notably, this HOMO extends to the remaining double bond of the allene system forming a helical connection.^[^ ^28^, ^30^ ^]^ Apparently, the larger coefficient of the HOMO is found at the central carbon of the allene rather than the terminal carbon. This unsymmetric distribution within the diene system was anticipated to induce regioselective reactivity when engaged with polarized reaction partners.

**Figure 2:**
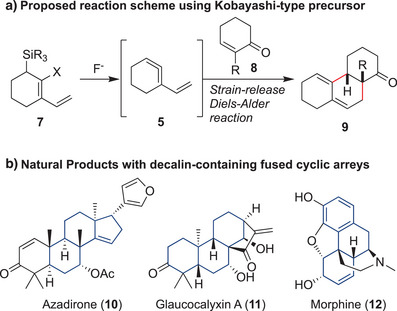
Diels–Alder reaction of a vinyl cyclic allene generated from a Kobayashi‐type precursor, which affords terpenoids relevant skeletons.

Based on this hypothesis, we planned the Diels–Alder reaction of the vinyl cyclic allene, generated in situ from the stable precursor, with a dienophile (Figure 2a). Considering the recent discoveries by Guitián, West, and Garg of a Kobayashi‐type cyclic allene precursor,^[^ ^35^, ^51^, ^52^ ^]^ we conceived of using a fluoride ion to trigger the generation of key fleeting vinyl cyclic allene **5** from precursor **7**. The reactive species **7** may easily undergo a Diels–Alder reaction with dienophiles such as enone **8** to give a decalin skeleton **9**. Although decalin synthesis using a Diels–Alder reaction is a common strategy,^[^ ^53^, ^54^, ^55^, ^56^ ^]^ an intermolecular approach often requires specialized conditions such as high temperature, high pressure, and a strong acid promoter with precisely designed substrates. The vinyl cyclic allene Diels–Alder reaction would provide an alternative, mild protocol for decalin construction. Additionally, we envisioned that the protocol using a cycloalkene as a dienophile could be an optimum solution for a convergent synthesis of polycyclic frameworks, which are frequently found in natural products and pharmaceuticals (Figure 2b, **10**–**12**). These skeletons often have an angular substituent, and thus, convergent synthesis through the intermolecular Diels–Alder reaction of two cyclic fragments is not trivial. We envisaged that the strain‐release Diels–Alder reaction of vinyl cyclic allene and cyclic dienophile could be a strong maneuver to solve various problems. Herein, we report a Diels–Alder reaction of vinyl cyclic allene. Basic reactivity, regio‐ and stereoselectivity of the cycloaddition, and mechanistic insight into this novel class of strained dienes are described.

## Results and Discussion

**Scheme 1:**
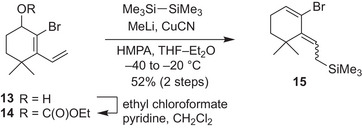
Preparation of the precursor **15**.

We began our research by synthesizing the precursor bearing an alkyl silyl group and a leaving group. Keeping a common terpene structure in mind, we designed a *gem*‐dimethyl substituted precursor **15** as a starting point. **15** was synthesized by carbonate formation and copper‐mediated silylation^[^ ^37^ ^]^ of known alcohol **13** ^[^ ^57^ ^]^ (Scheme 1). Unexpectedly, the silylation proceeded in a 1,5‐substitution manner, attacking the terminal carbon, and **15** was obtained as a mixture of *E/Z*‐isomers, which was used for the key reaction without separation. No S_N_2 and 1,3‐substitution product was obtained. Although the generation of cyclic allenes from this type of precursor is unreported, we envisaged the formation of active species through a vinylogous‐type elimination.^[^ ^58^, ^59^ ^]^

With the key precursor in hand, we screened the reaction conditions for the Diels–Alder reaction using 2‐cyclohexen‐1‐one as a dienophile. As shown in Table 1, when the precursor was treated with CsF at room temperature in a mixed solvent of THF and MeCN, the desired tricyclic product **16** was obtained in 58% yield as a single stereoisomer. Structure elucidation using NMR suggested that the *cis*‐fused structure was selectively obtained, supporting the concerted Diels–Alder mechanism. This result demonstrates that the 1,4‐elimination from “vinylogous” Kobayashi‐type precursor can indeed generate the strained cyclic allene under typical conditions. The major byproduct appeared to be a [2+2]‐type cycloadduct (**17**, mixture of isomers, proposed structure). This type of reaction has been reported in the literature to proceed via a stepwise open‐shell reaction pathway.^[^ ^29^ ^]^ The yield decreased when the reaction was conducted in MeCN, probably due to the low solubility of nonpolar precursor **15**. Other sources of F^−^ such as TBAF and TBAT(*n*‐Bu_4_NPh_3_SiF_2_) did not produce the desired cycloadduct in meaningful yield. The reaction temperature could be lowered to −40 °C in the presence of *n*‐Bu_4_NBr,^[^ ^43^ ^]^ but the best yield was obtained at room temperature.

**Table 1:** Diels–Alder reaction of the in‐situ‐generated vinyl cyclic allene with 2‐cyclohexenone.

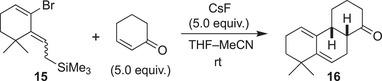		
Entry	Variation from the standard conditions^a)^	Yield (%)
1	None	58
2	MeCN instead of THF–MeCN	42
3	*n*‐Bu_4_NF instead of CsF	2^b)^
4	*n*‐Bu_4_NPh_3_SiF_2_ (2 equiv) instead of CsF	14^b)^
5	−20 °C instead of rt^c)^	36
6	−40 °C instead of rt^c)^	35
7	−60 °C instead of rt^c)^	0

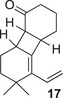

^a)^ Conditions: **15** (0.1 mmol), 2‐cyclohexene‐1‐one (0.5 mmol), CsF (0.5 mmol), MeCN–THF (3:2, 1 mL), rt, 22 h.

^b)^ NMR yield using 1,1,2‐trichloroethene as an internal standard.

^c)^ *n*‐Bu_4_NBr (0.4 equiv) was added.

**Figure 3:**
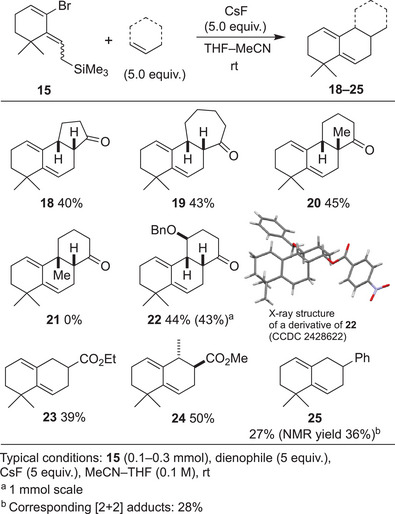
Scope and limitations of dienophiles.^[^ ^60^ ^]^

Subsequently, we investigated the reactivity of various dienophiles in this reaction (Figure 3). Not only 6‐membered ring enone but also 5‐ and 7‐membered enones afforded the corresponding Diels–Alder products (**18**, **19**). When 2‐methyl‐2‐cyclohexen‐1‐one was used, the tricyclic product bearing an angular substitution (**20**) was obtained. This result, demonstrating an intermolecular Diels–Alder reaction that constructs an all‐carbon quaternary center at room temperature, was particularly encouraging for us as this structural motif is frequently encountered in natural products such as diterpenoids. On the other hand, the Diels–Alder reaction of 3‐methyl‐2‐cyclohexen‐1‐one did not proceed to give the cycloadduct **21**, and decomposition was observed. The Diels–Alder reaction of 4‐substituted 2‐cyclohexene‐1‐one also proceeded smoothly, and cycloadduct **22** was obtained in a highly stereoselective manner. The reaction was also conducted on a 1 mmol scale, demonstrating the scalability of the Diels–Alder reaction. The stereochemistry of the product was determined based on the crystal structure of the derivative. Based on the observed stereoselectivity, the diene would approach the enone from the side opposite that the benzyl ether substituent occupied. Not only cyclic enones but also acyclic dienophiles, such as ethyl acrylate and methyl crotonate, produced decalin cycloadduct (**23**, **24**) with acceptable yields. In addition, styrene was found to function as a dienophile in the Diels–Alder reaction to give **25**, although a substantial amount of [2+2] cycloadducts were formed as byproducts.

**Figure 4:**
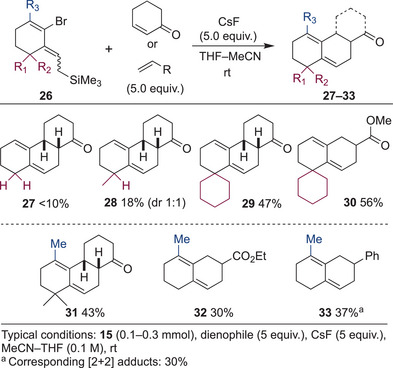
Scope and limitations of the substituted vinyl cyclic allenes.^[^ ^60^ ^]^

To understand the nature of the developed vinyl cyclic allene, the effect of the substituents in the Diels–Alder reaction was investigated (Figure 4). When the *gem‐*dimethyl group was removed from the ring, surprisingly, almost no Diels–Alder product was obtained (**27**). Although a small amount of the [2+2] adduct was observed, most of the material seems to be decomposed. According to the result, the *gem*‐dimethyl group might have two positive effects: stabilization of the strained double bond by steric shielding, and fixation of the configuration of the diene moiety in the s‐*cis* conformation, which results in acceleration of the productive pathway. To confirm the importance of the number of substitutions, a mono‐methylated substrate was also subjected to the reaction conditions in the presence of 2‐cyclohexene‐1‐one. Although the corresponding cycloadduct **28** was produced in this case, the yield was low (18%), supporting the hypothesis. In addition, the methyl group has little effect on stereoselectivity, and cycloadducts were obtained as a 1:1 mixture of diastereomers. Not surprisingly, *gem*‐disubstituted spirocyclic cyclic allene afforded the Diels–Alder products in good yield (**29**, **30**).

Next, a substituent at an allene carbon was examined. When the methyl group was attached to *gem*‐dimethyl substituted cyclic allene, the Diels–Alder reaction with 2‐cyclohexene‐1‐one proceeded smoothly to give a tricyclic compound **31** bearing a tetrasubstituted alkene. Interestingly, the formation of a [2+2] adduct was completely suppressed. This suppression might be attributed to both the electronic and steric effects of the methyl group. An increase in the electron density of the cyclic allene would facilitate the Diels–Alder reaction with an electron‐deficient alkene, whereas concurrently, the methyl group would hinder the [2+2] cycloaddition, which requires the formation of a quaternary center. This substituent effect was also observed for substrates lacking the *gem*‐dimethyl group. Although the Diels–Alder reaction with 2‐cyclohexene‐1‐one gave the desired product in less than 10% yield, the reaction with ethyl acrylate and styrene afforded the decalin products **32** and **33** in 30% and 37% yield, respectively. Interestingly, these methyl‐substituted series exhibited a propensity to form dimerized products. When the substrates were added to a reaction mixture in one portion, a significant amount of an isomeric mixture of dimers, presumably formed via a Diels–Alder reaction, was observed.^[^ ^48^ ^]^ Consequently, slow addition of the precursor solution to the reaction mixture using a syringe pump was effective to achieve the indicated yield.

**Scheme 2:**
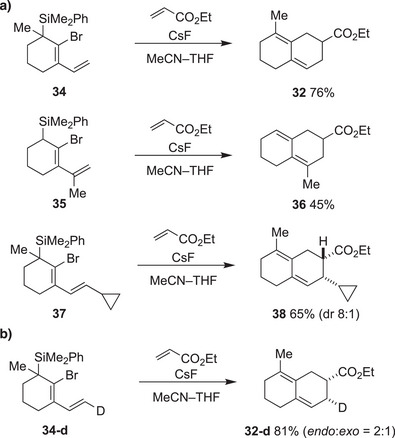
Alternative precursors for the Diels–Alder reaction of vinyl cyclic allene. a) Investigation of the substituent effects. b) Cycloaddition of a deuterium‐labelled vinyl cyclic allene.^[^ ^60^ ^]^

To further suppress the formation of dimer, we synthesized an alternative precursor for the vinylated cyclic allene possessing the dimethylphenylsilyl group at the cyclohexane ring with the intention of the slower generation of active species under the reaction conditions (Scheme 2a). Indeed, the reaction of the precursor **34** with methyl acrylate proceeds cleanly to give the Diels–Alder product **32** with improved yield without forming a dimeric byproduct. The substituent effect of a vinyl group was also examined using precursors bearing a dimethylphenylsilyl group. Isopropenyl cyclic allene was generated from precursor **35** and reacted with ethyl acrylate. As a result, despite having no substituents on the cyclohexane ring, the Diels–Alder product **36** was obtained in 45% yield. This result suggests that the substituent on this position might increase the proportion of s‐*cis* conformation of the diene, facilitating the Diels–Alder reaction. Additionally, terminal substitution was also accepted. The Diels–Alder reaction of vinyl cyclic allene having a cyclopropane substituent at the terminal position of the alkene proceeded smoothly to give cycloadduct **38**. The reaction proceeded stereoselectively (ca. 8:1 dr) to give the *endo*‐product as a major isomer. Importantly, the cyclopropane ring remained intact after the reaction, suggesting that the [4+2]‐cycloaddition does not involve a radical intermediate. To understand the *endo*/*exo* selectivity of the simple vinyl cyclic allene without a terminal substituent, a deuterium‐labeling experiment was also performed (Scheme 2b). The precursor **34‐d**, which has a deuterium at the terminal carbon, was synthesized and exposed to the reaction conditions. As a result, the cycloadduct **32‐d** was obtained as a mixture of diastereomers, and based on the NMR analysis, the *endo*‐product was found to be the major product, albeit with low selectivity (*endo*/*exo* = 2:1). Overall, the substituents at each carbon of vinyl cyclic allene were well tolerated, and structurally diverse decalin derivatives were synthesized using the Diels–Alder reaction.

**Figure 5:**
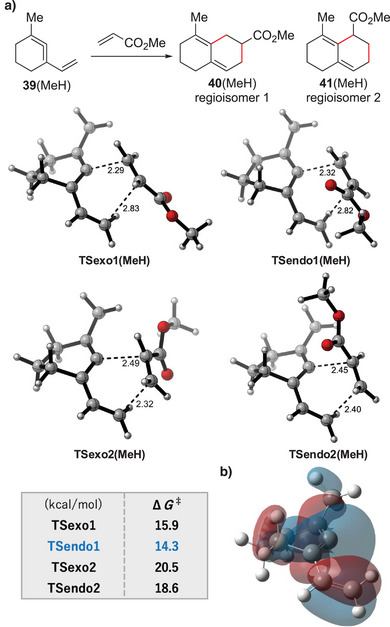
DFT calculations of the vinyl cyclic allene Diels–Alder reactions. a) Transition state (TS) structures and their energy profiles. Energies in kcal mol^−1^ are for the ωB97XD/6‐311+G(d,p)/SMD(MeCN) level. The distance between atoms is indicated in Å. b) Visualized isosurface of HOMO of vinyl cyclic allene **39**.

To gain insights into the reaction mechanism and selectivities involved in cycloaddition, DFT calculations were performed. The Diels–Alder reaction between methyl‐substituted vinyl cyclic allene (1‐methyl‐3‐vinylcyclohexa‐1,2‐diene) **39** (MeH) and methyl acrylate was chosen as a model system (Figure 5a), and *endo*‐ and *exo*‐pathways yielding two regioisomers were calculated. Geometry optimizations were carried out using the ωB97X‐D^[^ ^61^ ^]^ functional and the 6‐31G(d) basis set combined with SMD^[^ ^62^ ^]^ solvation model in acetonitrile (MeCN). The single point energies were computed at the ωB97XD/6‐311+G(d,p) basis set with SMD solvation model in MeCN. The transition state (TS) structures are depicted in Figure 5a with the labels TSexo1(MeH), TSendo1(MeH), TSexo2(MeH), and TSendo2(MeH). We found that the concerted cycloaddition preferably proceeds through TSendo1 with a reasonable energy barrier. The trend in Gibbs free energy barriers (*ΔG^‡^* in kcal mol^−1^) was endo‐1 (14.3) < exo‐1 (15.9) << endo‐2 (18.6) < exo‐2 (20.5). As the experiment produces the *endo*‐product of regioisomer‐1 as the major product and the *exo*‐product of regioisomer‐1 as a minor product without affording the regioisomer‐2, this calculated trend could be in good agreement.^[^ ^63^ ^]^ The origin of this regioselectivity could be consider based on a distribution of coefficient of HOMO of the vinyl cyclic allene **39**, which is more concentrated on the allene central carbon (Figure 5b, see Supporting Information for the details). The Diels–Alder reaction with methyl acrylate, which has a larger coefficient of the LUMO at the β‐carbon, would prefer to generate regioisomer‐1 over regioisomer‐2. In addition, Bickelhaupt, Fernández, and Hamlin previously proposed that the asynchronicity of the Diels–Alder reaction is connected to the energy barriers through the degree of structural deformation of the reactants. Specifically, a higher degree of asynchronicity leads to a lower energy barrier compared to the synchronous transition state.^[^ ^64^, ^65^ ^]^ In our system, the difference in the distance between the two forming carbon–carbon bonds in the calculated transition states are 0.54 and 0.50 Å for TSexo1 and TSendo1, respectively, and 0.17 and 0.05 Å for TSexo2 and TSendo2, respectively. These results indicate that TSexo1 and TSendo1 are significantly more asynchronous than TSexo2 and TSendo2. Accordingly, a similar relationship between asynchronicity and *ΔG^‡^* appears to be present in our vinyl cyclic allene Diels–Alder reaction, with the more asynchronous TSexo1 and TSendo1 showing lower activation barriers than their more synchronous counterparts.

To further elucidate the origin of kinetic preference for this cycloaddition step, we turned to the activation strain model (ASM). The ASM involves decomposition of the electronic energy Δ*E* into the strain energy Δ*E* ^‡^_strain_ associated with the structural deformation of the cyclic allene **39** and methyl acrylate from their equilibrium geometry and the interaction energy Δ*E* ^‡^_int_ between these deformed reactants (Equation 1):^[^ ^47^, ^66^, ^67^, ^68^, ^69^, ^70^, ^71^ ^]^

(1) $$\Delta E^{‡}=\Delta E_{\mathrm{strain}}^{‡}+\Delta E_{\mathrm{int}}^{‡}$$

As the electronic activation barriers (Δ*E* ^‡^) follow the same trend as Δ*G* ^‡^, the differences in reactivity can be traced back to the differences in the strain energy (Δ*E* ^‡^_strain_) and the interaction energies (Δ*E* ^‡^_int_).

**Figure 6:**
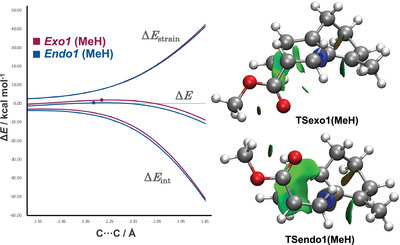
Activation strain model (left) for the *exo*1 and *endo*1 pathway for the Diels–Alder reaction of **39** and methyl acrylate (positions of TS indicated with a dot), and NCIplot isosurface of the calculated TSexo1 and TSendo1 (right). The green surface indicates weak attractive interactions.

The ASM of the Diels–Alder reaction of **39** and methyl acrylate revealed that the overall energy between the *exo*‐pathway and *endo*‐pathway is mainly affected by Δ*E*_int_, and Δ*E*_strain_ is similar over the course of the reaction (Figure 6, left). NCIPLOT analysis,^[^ ^72^, ^73^ ^]^ which indicates weak attractive interactions in the form of green surfaces, suggested the larger interaction between diene and dienophile was observed in TSendo1 (Figure 6, right). This might arise from a secondary orbital interaction involving the polar C–O double bond of the dienophile, which is commonly observed in the Diels–Alder reaction using an electron‐deficient dienophile.

**Figure 7:**
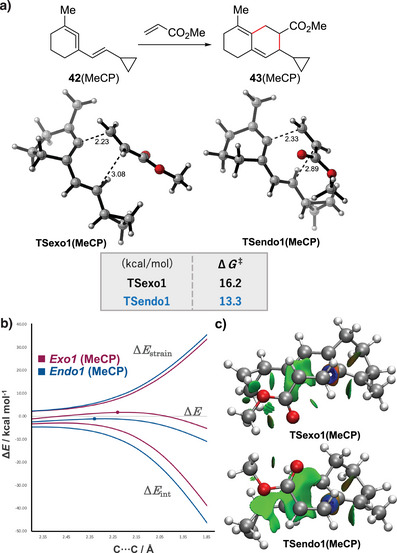
DFT calculations of the vinyl cyclic allene Diels–Alder reactions of **42**. a) Transition state (TS) structures and their energy profiles. Energies in kcal mol^−1^ are for the ωB97XD/6‐311+G(d,p)/SMD(MeCN) level. The distance between atoms is indicated in Å. b) Activation strain model (left) for the *exo*1 and *endo*1 pathway for the Diels–Alder reaction of **42** and methyl acrylate (positions of TS indicated with a dot), and NCIplot isosurface of the calculated TSexo1 and TSendo1 (right). The green surface indicates weak attractive interactions.

Next, transition state energies for the Diels–Alder reaction of cyclopropane‐substituted vinyl cyclic allene (**42**, MeCP) were calculated (Figure 7a). Although the overall trend in Δ*G* ^‡^ remained consistent with the result discussed above (see Supporting Information for details), ΔΔ*G* ^‡^ between TSendo1(MeCP) and TSexo1(MeCP) was more pronounced compared to that for MeH, correlating well with the experimental observations. As the electronic activation barriers (Δ*E* ^‡^) follow the same trend as Δ*G* ^‡^, the difference in energy between *endo*‐ and *exo*‐TS was analyzed using ASM. Again, the difference in reactivity was primarily attributed to differences in interaction energy (Δ*E* ^‡^_int_). NCIPLOT of the transition states revealed secondary orbital interactions in TSendo1, which may contribute to lowering the activation barrier along the *endo*‐pathway. Interestingly, the *endo* transition state appeared significantly earlier than the *exo* counterpart, as indicated by the forming bond distances (2.23 Å for exo1 and 2.33 Å for endo1). This might be due to the aforementioned attractive interactions, as well as steric repulsion between a cyclopropane group of a diene and an ester group of a dienophile in the *exo*‐pathway, which could destabilize the transition state and shift it later along the reaction coordinate.

## Conclusion

In conclusion, we have discovered a vinyl group‐conjugated cyclic allene (1‐vinyl‐1,2‐cyclohexadiene) as a unique highly strained 1,2,4‐triene system. Unlike the many fleeting species bearing highly strained π bonds that are typically used as a 2π‐component in a variety of reactions, the vinylated cyclic allene possesses reactivity as a 4π‐component. Consequently, the species participates in a Diels–Alder reaction as an electronically nonactivated but highly reactive diene. This reactivity allowed us to develop a room‐temperature, intermolecular Diels–Alder reaction to construct a multicyclic skeleton, represented by the construction of a terpene‐like tricyclic skeleton with angular substitution. Experimental and computational analyses revealed the scope and limitations of the Diels–Alder reaction with regio‐ and stereoselectivity to provide an understanding of the nature of the new strained diene. These results may lead to a useful synthetic method to combine a variety of units intermolecularly in an alternative fashion under mild conditions. Further studies to understand the nature of the vinyl cyclic allene, such as a detailed mechanistic study, expansion of the vinyl cyclic allene system to structural variants such as heterocyclic congeners (e.g., vinylated azacyclic allene), and application of the cycloaddition for the synthesis of complex molecules, are ongoing.

## Supporting Information

Preparation procedures and characterization data for all new compounds, computational methodology and data, and copies of ^1^H and ^13^C NMR spectra are provided in the Supporting Information.

## Conflict of Interests

The authors declare no conflict of interest.

## Supporting information



Supporting Information

Supporting Information

## Data Availability

The data that support the findings of this study are available in the Supporting Information of this article.

## References

[1] J. F. Liebman , A. Greenberg , Chem. Rev. 1976, 76, 311–365.

[2] K. B. Wiberg , Angew. Chem. Int. Ed. 1986, 25, 312–322.

[3] J. Turkowska , J. Durka , D. Gryko , Chem. Commun. 2020, 56, 5718–5734. 10.1039/d0cc01771j 32391543

[4] S. M. Anthony , L. G. Wonilowicz , M. S. McVeigh , N. K. Garg , JACS Au 2021, 1, 897–912. 34337603 10.1021/jacsau.1c00214 PMC8317162

[5] L. McDermott , Z. G. Walters , A. M. Clark , N. K. Garg , Nat. Synth. 2025, 4, 421–431. 10.1038/s44160-025-00776-w PMC12459645 41000096

[6] M. R. Wilson , R. E. Taylor , Angew. Chem. Int. Ed. 2013, 52, 4078–4087. 10.1002/anie.201207712 23450661

[7] P. M. Tadross , B. M. Stoltz , Chem. Rev. 2012, 112, 3550–3577. 22443517 10.1021/cr200478h

[8] C. M. Gampe , E. M. Carreira , Angew. Chem. Int. Ed. 2012, 51, 3766–3778. 10.1002/anie.201107485 22422638

[9] H. Takikawa , A. Nishii , T. Sakai , K. Suzuki , Chem. Soc. Rev. 2018, 47, 8030–8056. 30357181 10.1039/c8cs00350e

[10] Y. Mizukoshi , K. Mikami , M. Uchiyama , J. Am. Chem. Soc. 2015, 137, 74–77. 25459083 10.1021/ja5112207

[11] D. Pérez , D. Peña , E. Guitián , Euro. J. Org. Chem. 2013, 2013, 5981–6013.

[12] K. Y. Cheung , K. Watanabe , Y. Segawa , K. Itami , Nat. Chem. 2021, 13, 255–259. 33495606 10.1038/s41557-020-00627-5

[13] J. C. Jewett , C. R. Bertozzi , Chem. Soc. Rev. 2010, 39, 1272. 20349533 10.1039/b901970g PMC2865253

[14] N. J. Agard , J. A. Prescher , C. R. Bertozzi , J. Am. Chem. Soc. 2004, 126, 15046–15047. 15547999 10.1021/ja044996f

[15] K. Adhikari , M. Vanermen , G. D.a Silva , T. Van den Wyngaert , K. Augustyns , F. Elvas , EJNMMI Radiopharm. Chem. 2024, 9, 47. 38844698 10.1186/s41181-024-00275-x PMC11156836

[16] M. F. Debets , S. S. van Berkel , J. Dommerholt , A. T. J. Dirks , F. P. J. T. Rutjes , F. L. van Delft , Acc. Chem. Res. 2011, 44, 805–815. 21766804 10.1021/ar200059z

[17] H. H. Wenk , M. Winkler , W. Sander , Angew. Chem. Int. Ed. 2003, 42, 502–528. 10.1002/anie.200390151 12569480

[18] J. Shi , L. Li , Y. Li , Chem. Rev. 2021, 121, 3892–4044. 33599472 10.1021/acs.chemrev.0c01011

[19] J. M. Medina , T. C. McMahon , G. Jiménez‐Osés , K. N. Houk , N. K. Garg , J. Am. Chem. Soc. 2014, 136, 14706–14709. 25283710 10.1021/ja508635v PMC4207212

[20] G. Wittig , P. Fritze , Angew. Chem. Int. Ed. 1966, 5, 846–846.

[21] R. P. Johnson , Chem. Rev. 1989, 89, 1111–1124.

[22] A. V. Kelleghan , A. S. Bulger , D. C. Witkowski , N. K. Garg , Nature 2023, 618, 748–754. 37075803 10.1038/s41586-023-06075-8 PMC10460091

[23] D. C. Witkowski , D. W. Turner , A. S. Bulger , K. N. Houk , N. K. Garg , Nat. Synth. 2025, 1–8. 10.1038/s44160-025-00793-9 PMC12459638 41000355

[24] A. E. Goetz , T. K. Shah , N. K. Garg , Chem. Commun. 2015, 51, 34–45. 10.1039/c4cc06445c 25226878

[25] T. C. McMahon , J. M. Medina , Y.‐F. Yang , B. J. Simmons , K. N. Houk , N. K. Garg , J. Am. Chem. Soc. 2015, 137, 4082–4085. 25768436 10.1021/jacs.5b01589 PMC4502428

[26] S. K. Thompson , T. R. Hoye , J. Am. Chem. Soc. 2019, 141, 19575–19580. 31789026 10.1021/jacs.9b11243 PMC6921493

[27] M. Nendel , L. M. Tolbert , L. E. Herring , M. N. Islam , K. N. Houk , J. Org. Chem. 1999, 64, 976–983. 11674172 10.1021/jo982091c

[28] J. S. Barber , M. M. Yamano , M. Ramirez , E. R. Darzi , R. R. Knapp , F. Liu , K. N. Houk , N. K. Garg , Nat. Chem. 2018, 10, 953–960. 30061614 10.1038/s41557-018-0080-1 PMC6317513

[29] M. S. McVeigh , J. P. Sorrentino , A. T. Hands , N. K. Garg , J. Am. Chem. Soc. 2024, 146, 15420–15427. 38768558 10.1021/jacs.4c03369 PMC11459239

[30] M. Ramirez , D. Svatunek , F. Liu , N. K. Garg , K. N. Houk , Angew. Chem. Int. Ed. 2021, 60, 14989–14997. 10.1002/anie.202101809 PMC8217342 33851504

[31] M. Christl , H. Fischer , M. Arnone , B. Engels , Chem. Eur. J. 2009, 15, 11266–11272. 19746463 10.1002/chem.200900718

[32] J. S. Barber , E. D. Styduhar , H. V. Pham , T. C. McMahon , K. N. Houk , N. K. Garg , J. Am. Chem. Soc. 2016, 138, 2512–2515. 26854652 10.1021/jacs.5b13304 PMC4899974

[33] V. A. Lofstrand , F. G. West , Chem. Eur. J. 2016, 22, 10763–10767. 27219685 10.1002/chem.201602201

[34] M. M. Yamano , R. R. Knapp , A. Ngamnithiporn , M. Ramirez , K. N. Houk , B. M. Stoltz , N. K. Garg , Angew. Chem. Int. Ed. 2019, 58, 5653–5657. 10.1002/anie.201900503 PMC6456397 30811080

[35] Y. A. Almehmadi , F. G. West , Org. Lett. 2020, 22, 6091–6095. 32790431 10.1021/acs.orglett.0c02172

[36] V. A. Lofstrand , K. C. McIntosh , Y. A. Almehmadi , F. G. West , Org. Lett. 2019, 21, 6231–6234. 31343882 10.1021/acs.orglett.9b02085

[37] C. L. Jankovic , F. G. West , Org. Lett. 2022, 24, 9497–9501. 36519787 10.1021/acs.orglett.2c03978

[38] A. V. Kelleghan , A. T. Meza , N. K. Garg , Nat. Synth. 2024, 3, 329–336. 38645473 10.1038/s44160-023-00432-1 PMC11031199

[39] C. L. Jankovic , K. C. McIntosh , V. A. Lofstrand , F. G. West , Chem. Eur. J. 2023, 29, e202301668. 37352092 10.1002/chem.202301668

[40] D. C. Witkowski , M. S. McVeigh , G. M. Scherer , S. M. Anthony , N. K. Garg , J. Am. Chem. Soc. 2023, 145, 10491–10496. 37141000 10.1021/jacs.3c03102 PMC10460090

[41] M. M. Yamano , A. V. Kelleghan , Q. Shao , M. Giroud , B. J. Simmons , B. Li , S. Chen , K. N. Houk , N. K. Garg , Nature 2020, 586, 242–247. 32846425 10.1038/s41586-020-2701-2 PMC8297713

[42] K. A. Spence , A. T. Meza , N. K. Garg , Chem Catal 2022, 2, 1870–1879. 36386492 10.1016/j.checat.2022.06.014 PMC9645731

[43] F. M. Ippoliti , N. J. Adamson , L. G. Wonilowicz , D. J. Nasrallah , E. R. Darzi , J. S. Donaldson , N. K. Garg , Science 2023, 379, 261–265. 36656952 10.1126/science.ade0032 PMC10462259

[44] F. M. Ippoliti , L. G. Wonilowicz , N. J. Adamson , E. R. Darzi , J. S. Donaldson , D. J. Nasrallah , M. M. Mehta , A. V. Kelleghan , K. N. Houk , N. K. Garg , Angew. Chem. Int. Ed. 2024, 63, e202406676. 10.1002/anie.202406676 PMC11461081 38695853

[45] M. M. Mehta , J. A. M. Gonzalez , J. L. Bachman , N. K. Garg , Org. Lett. 2023, 25, 5553–5557. 37387644 10.1021/acs.orglett.3c01489 PMC10460088

[46] M. V. Westphal , L. Hudson , J. W. Mason , J. A. Pradeilles , F. J. Zécri , K. Briner , S. L. Schreiber , J. Am. Chem. Soc. 2020, 142, 7776–7782. 32267148 10.1021/jacs.9b13186 PMC7294439

[47] F. Liu , R. S. Paton , S. Kim , Y. Liang , K. N. Houk , J. Am. Chem. Soc. 2013, 135, 15642–15649. 24044412 10.1021/ja408437u

[48] B. Wang , M.‐G. Constantin , S. Singh , Y. Zhou , R. L. Davis , F. G. West , Org. Biomol. Chem. 2021, 19, 399–405. 33300539 10.1039/d0ob02285c

[49] While highly strained cyclobutadiene exhibits high reactivity as a conjugated diene, its pronounced reactivity can be attributed to its antiaromatic character as well. For the synthesis and reaction of metal‐free cyclobutadiene, see: B. R. Boswell , C. M. F. Mansson , G. E. Cabrera , C. R. Hansen , A. G. Oliver , N. Z. Burns , J. Am. Chem. Soc. 2023, 145, 5631–5636. 36856576 10.1021/jacs.3c01591

[50] K. J. Daoust , S. M. Hernandez , K. M. Konrad , I. D. Mackie , J. Winstanley Jr , R. P. Johnson , J. Org. Chem. 2006, 71, 5708–5714. 16839152 10.1021/jo060698k

[51] I. Quintana , D. Peña , D. Pérez , E. Guitián , Euro. J. Org. Chem. 2009, 2009, 5519–5524.

[52] D. Peña , B. Iglesias , I. Quintana , D. Pérez , E. Guitián , L. Castedo , Pure Appl. Chem. 2006, 78, 451–455.

[53] M. M. Heravi , V. F. Vavsari , RSC Adv. 2015, 5, 50890–50912.

[54] A. A. Sara , U.‐E.‐F. Um‐e‐Farwa , A. Saeed , M. Kalesse , Synthesis 2022, 54, 975–998.

[55] S. Rashid , W. I. Lone , A. Rashid , B. A. Bhat , Tetrahedron Chem. 2024, 9, 100066.

[56] M. Sherburn , E. Mackay , Synthesis 2014, 47, 1–21.

[57] N. Hatae , I. Suzuki , T. Choshi , S. Hibino , C. Okada , E. Toyota , Tetrahedron Lett. 2014, 55, 4146–4148.

[58] M. Bamba , T. Nishikawa , M. Isobe , Tetrahedron 1998, 54, 6639–6650.

[59] W. S. Trahanovsky , J. R. Macias , J. Am. Chem. Soc. 1986, 108, 6820–6821.

[60] A minor amount of [2+2]‐cycloadducts was also observed in some examples. Diels–Alder reaction was highly stereoselective and no detectable amount of diastereomers was obtained except for the indicated examples.

[61] J.‐D. Chai , M. Head‐Gordon , Phys. Chem. Chem. Phys. 2008, 10, 6615. 18989472 10.1039/b810189b

[62] A. V. Marenich , C. J. Cramer , D. G. Truhlar , J. Phys. Chem. B 2009, 113, 6378–6396. 19366259 10.1021/jp810292n

[63] Although the magnitude of ΔΔG‡ between endo‐1 and exo‐1 differs from the experimentally determined endo/exo ratio, this level of theory provided reliable results among the theories tested, and referring to the detailed precedent work by Houk and Garg,^28^ and Piquemal and Spezia, we chose ωB97XD as a functional for further calculations. D. Loco , I. Chataigner , J.‐P. Piquemal , R. Spezia , ChemPhysChem 2022, 23, e202200349. 35696652 10.1002/cphc.202200349 PMC9796631

[64] P. Vermeeren , T. A. Hamlin , I. Fernández , F. M. Bickelhaupt , Chem. Sci. 2020, 11, 8105–8112. 34094173 10.1039/d0sc02901g PMC8163289

[65] P. Vermeeren , T. A. Hamlin , I. Fernández , F. M. Bickelhaupt , Angew. Chem. Int. Ed. 2020, 59, 6201–6206. 10.1002/anie.201914582 PMC7187354 31944503

[66] W.‐J. van Zeist , F. M. Bickelhaupt , Org. Biomol. Chem. 2010, 8, 3118. 20490400 10.1039/b926828f

[67] I. Fernández , F. M. Bickelhaupt , Chem. Soc. Rev. 2014, 43, 4953–4967. 24699791 10.1039/c4cs00055b

[68] I. Fernández , F. M. Bickelhaupt , Chem. Asian J. 2016, 11, 3297–3304. 27863108 10.1002/asia.201601203

[69] F. M. Bickelhaupt , K. N. Houk , Angew. Chem. Int. Ed Engl. 2017, 56, 10070–10086. 28447369 10.1002/anie.201701486 PMC5601271

[70] P. Vermeeren , S. C. C. van der Lubbe , C. F. Guerra , F. M. Bickelhaupt , T. A. Hamlin , Nat. Protoc. 2020, 15, 649–667. 31925400 10.1038/s41596-019-0265-0

[71] A. M. Sarotti , Org. Biomol. Chem. 2014, 12, 187–199. 24085334 10.1039/c3ob41628c

[72] E. R. Johnson , S. Keinan , P. Mori‐Sánchez , J. Contreras‐García , A. J. Cohen , W. Yang , J. Am. Chem. Soc. 2010, 132, 6498–6506. 20394428 10.1021/ja100936w PMC2864795

[73] J. Contreras‐García , E. R. Johnson , S. Keinan , R. Chaudret , J.‐P. Piquemal , D. N. Beratan , W. Yang , J. Chem. Theory Comput. 2011, 7, 625–632. 21516178 10.1021/ct100641a PMC3080048

