# Supplementary material for: Development of a Vinylated Cyclic Allene: A Fleeting Strained Diene for the Diels–Alder Reaction
Source: Angew Chem Int Ed Engl. 2025 Jun 26;64(33):e202510319. doi: 10.1002/anie.202510319 (PMC12338423; doi:10.1002/anie.202510319)
Supplement: Supplementary file 1 — Supporting Information [file ANIE-64-e202510319-s001.pdf]

**Supporting Information**

# Development of a Vinylated Cyclic Allene: A Fleeting Strained Diene for the Diels-Alder Reaction

Haruki Mizoguchi*, Takumi Obata, Taiki Hirai, Manaka Komatsu, and Akira Sakakura*

## Table of contents


| | |
|---|---|
| General Methods | S2 |
| Optimization of reaction conditions | S3 |
| Preparation of substrates | S4–S15 |
| Procedures for CsF mediated Strain release Diels-Alder reaction | S16–S26 |
| References | S27 |
| Computational Study | S28–S51 |
| Crystallographic Data | S52–S53 |
| $^{1}H$ and $^{13}C$ NMR Spectra of New Compounds | S54–S88 |

## General Methods

IR spectra were recorded on a SHIMADZU FTIR-8400 spectrometer. $^{1}H$ NMR spectra were measured on JEOL JNMECZ600R spectrometer (600 MHz), Varian NMR System 600 PS600 spectrometer (600 MHz), a Varian 400-MR ASW spectrometer (400 MHz). Data were recorded as follows: chemical shift in ppm from the solvent resonance employed as the internal standard ($CHCl_3$ at 7.26 ppm) on the $\delta$ scale, multiplicity (s = singlet; d = doublet; t = triplet; m = multiplet), coupling constant (Hz), and integration. $^{13}C$ NMR spectra were measured on JEOL JNMECZ600R spectrometer (150* MHz), Varian NMR System 600 PS600 spectrometer (150 MHz) and a Varian 400-MR ASW spectrometer (100 MHz) at ambient temperature. Chemical shifts were recorded in ppm from the solvent resonance employed as the internal standard ($CDCl_3$ at 77.16 ppm). For TLC analysis, Merck precoated TLC plates (silica gel 60 $F_{254}$ 0.25 mm) and Wako precoated TLC places (silica gel 60 $F_{254}$ 0.25 mm) were used. For preparative column chromatography, Fuji Silysia Chemical PSQ60B, Fuji Silysia Chemical PSQ100B, and Kanto Chemical Co., Inc. silica gel 60 (spherical) $NH_2$ were used. High- and low-resolution mass spectral analysis (HRMS) was measured on a Bruker micrOTOF II (ESI) at the Chemical Instrument Facility, Okayama University. Dry toluene, tetrahydrofuran (THF), dichloromethane ($CH_2Cl_2$), dimethyl sulfoxide (DMSO), methanol (MeOH), diethyl ether ($Et_2O$), ethyl acetate (EtOAc) and chloroform ($CHCl_3$) were purchased from Kanto Chemical Co., Inc. or Wako Pure Chemical Industries Ltd. as the "anhydrous" and stored under nitrogen. Other materials were obtained from commercial supplies and used without further purification. All reactions were conducted in flame-dried glassware under a nitrogen atmosphere, otherwise noted.

## Optimization of the reaction conditions

**Table S1. Reaction condition optimizations**

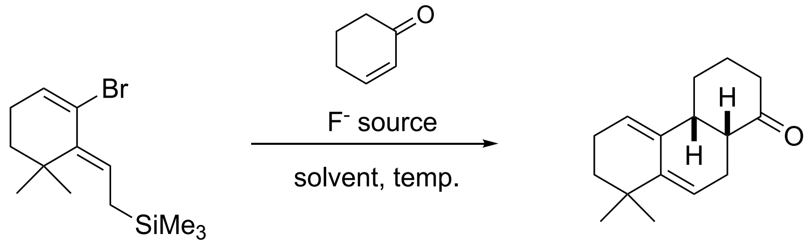


| entry | Solvent | $F^-$ source | Temp. | yield [*2] |
|---|---|---|---|---|
| 1 | MeCN | CsF | rt | 42% |
| 2 | MeCN-THF | CsF | rt | 58% |
| 3[*2] | MeCN-THF | *n*-$Bu_4NF$ | rt | 2% |
| 4[*2] | MeCN-THF | TASF | rt | 14% |
| 5[*1] | MeCN-THF | CsF | −20 °C | 36% |
| 6[*1] | MeCN-THF | CsF | −40 °C | 35% |
| 7[*1,3] | MeCN-THF | CsF | −60 °C | 0% |

[*1] *n*-$Bu_4NBr$ (40 mol%) was added [*2] NMR yield using 1,1,2-trichloroethene as an internal standard
[*3] EtCN was used instead of MeCN

## Preparation of substrates

***rac*-2-bromo-4,4-dimethyl-3-vinylcyclohex-2-en-1-ol (13)**

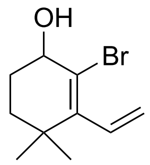


**13**

**13** was prepared according to a reported procedure.[74]

***rac*-2-bromo-3-vinylcyclohex-2-en-1-ol (S1)**

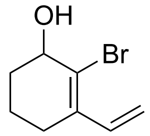


**S1**

**S1** was prepared according to a reported procedure.[75]

***rac*-4-(benzyloxy)cyclohex-2-en-1-one (S25)**

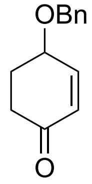


**S25**

**S25** was prepared according to a reported procedure.[75]

***rac-2-bromo-4,4-dimethyl-3-vinylcyclohex-2-en-1-yl ethyl carbonate* (14)**

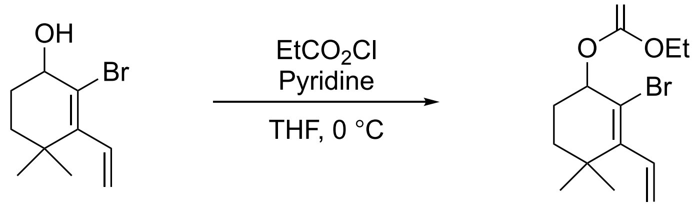


To a solution of alcohol **13** (2.06 g, 8.92 mmol) in $CH_2Cl_2$ (89 mL) was added pyridine (1.7 mL, 21.1 mmol) and ethyl chloroformate (2.0 mL, 21.4 mmol) at 0 °C. After being stirred at 0 °C for 5 min, the

mixture was warmed to room temperature and stirred for 1 h. The reaction was quenched with saturated aqueous solution of $NaHCO_3$. The mixture was extracted with $CH_2Cl_2$ and the combined organic layer was washed with brine, dried over $Na_2SO_4$, filtered, and concentrated under reduced pressure. The crude mixture was purified by column chromatography on silica gel to obtain carbonate **14** (2.70 g, 8.91 mmol, quant) as a white oil.

**14**: IR (film) 2969, 2890, 1744, 1453, 1371, 1236, 1167, 1019, 855, 768, 490; $^1$H NMR (400 MHz, $CDCl_3$) δ 6.13 (ddd, *J* = 17.6, 12.0, 1.2 Hz, 1H), 5.36 (dd, *J* = 12.0, 1.6 Hz, 1H), 5.27 (dd, *J* = 17.6, 1.6 Hz, 1H), 5.31–5.27 (m, 1H), 4.20 (dq, *J* = 7.2, 2.0 Hz, 2H), 2.48 (app t, *J* = 7.2 Hz, 1H), 2.11–1.88 (m, 2H), 1.74 (td, *J* = 13.2, 3.2 Hz, 1H), 1.45 (dddd, *J* = 14.0, 5.6, 3.2, 0.4 Hz, 1H), 1.30 (t, *J* = 7.2 Hz, 3H), 1.071 (s, 3H), 1.067 (s, 3H); $^{13}$C NMR (100 MHz, $CDCl_3$) δ 154.7, 149.2, 134.3, 120.6, 117.5, 76.8, 64.1, 37.7, 33.7, 28.6, 26.4, 26.1, 14.2; HRMS (ESI) *m/z* calcd for $C_{13}H_{19}BrNaO_3$ [M+Na]$^+$ 325.0415, found 325.0413.

***rac-(2-(2-bromo-6,6-dimethylcyclohex-2-en-1-ylidene)ethyl)trimethylsilane*** **(15)**

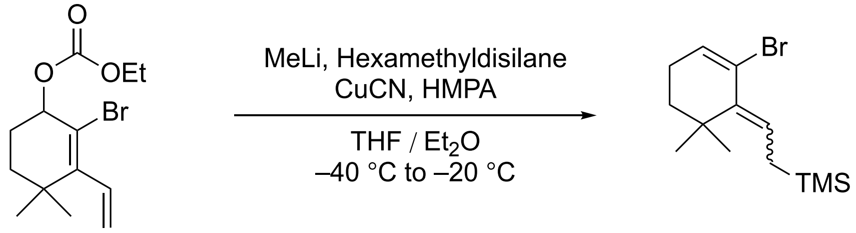


**14** **15**

To a solution of hexamethyldisilane (0.76 mL, 3.73 mmol) in $Et_2O$ (8.0 mL) and HMPA (8.0 mL) was added MeLi (3.1 M solution in diethoxymethane, 1.2 mL, 3.73 mmol) dropwise at 0 °C and stirred for 20 min. The solution was added to another flask containing a suspension of CuCN (167 mg, 1.86 mmol) in $Et_2O$ (8.0 mL) and the resulting suspension was stirred for 20 min before it was cooled down to –40 °C.[76] To the suspension of silyl-cuprate was added carbonate **14** (565 mg, 1.86 mmol) in THF (15 mL), which was precooled to –40 °C, dropwise. The reaction was warmed up to 0 °C and stirred for 3 h. The reaction was quenched with saturated aqueous solution of $NaHCO_3$. The mixture was extracted with hexane and the combined organic layer was washed with brine, dried over $Na_2SO_4$, filtered, and concentrated under reduced pressure. The crude mixture was purified by column chromatography on alumina to obtain allylsilane **15** (276 mg, 0.96 mmol, ca. 1:0.7 mixture of *E/Z*-isomers, 52%) as a colorless oil.

**15**: IR (film) 2955, 2920, 2334, 1464, 1437, 1360, 1339, 1248, 1138, 1082, 1057, 1020, 1010, 853, 760, 693, 530, 484, 452; $^1$H NMR (400 MHz, $CDCl_3$) δ 6.19 (t, *J* = 4.8 Hz, 1H), 6.14–6.06 (m, 1.7H), 5.53 (t, *J* = 9.2 Hz, 0.7H), 2.23–2.11 (m, 5.4H), 1.86 (d, *J* = 9.6 Hz, 1.7H), 1.54–1.43 (m, 3.4H), 1.25 (s, 6H), 1.08 (s, 4.2H), 0.07 (s, 9H), 0.03 (s, 6.3H); $^{13}$C NMR (150 MHz, $CDCl_3$) δ 137.8, 133.2, 124.7, 118.3, 38.2, 37.1, 27.2, 26.3, 21.3, -1.3; HRMS (ESI, APCI) peaks were not detectable.

***rac-2-bromo-1,4,4-trimethyl-3-vinylcyclohex-2-en-1-yl acetate* (S4)**

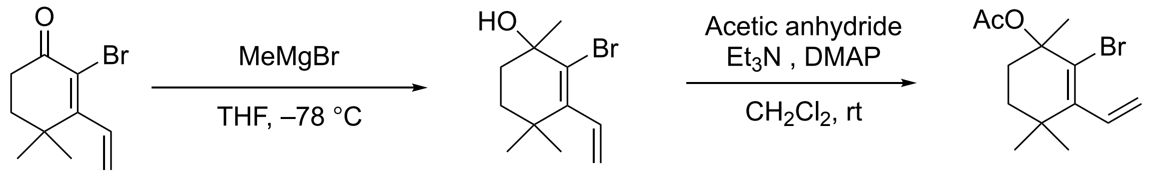


**S2** **S3** **S4**

To a solution of ketone **S2** (981 mg, 4.28 mmol) in THF (4.2 mL) was added MeMgBr (1.0 M solution in THF, 5.13 mL, 5.13 mmol) –78 °C. The mixture was warmed up to room temperature over 8 h and stirred for 12 h at the temperature. The reaction was quenched with saturated aqueous solution of $NH_4Cl$. The mixture was extracted with $Et_2O$ and the combined organic layer was washed with brine, dried over $Na_2SO_4$, filtered, and concentrated under reduced pressure. The crude mixture was purified by column chromatography on silica gel to obtain alcohol **S3** (480 mg), which contains some impurities. This mixture was dissolved in $CH_2Cl_2$ (3.0 mL) and treated with $Et_3N$ (0.54 mL, 3.90 mmol), $Ac_2O$ (0.4 mL, 3.90 mmol) and DMAP (24.4 mg, 0.20 mmol) at 0 °C. The mixture was warmed up to room temperature and stirred for 18 h. The reaction was quenched with saturated aqueous solution of $NaHCO_3$. The mixture was extracted with $Et_2O$, and the combined organic layer was washed with brine, dried over $Na_2SO_4$, filtered, and concentrated under reduced pressure. The crude mixture was purified by column chromatography on alumina to obtain acetate **S4** (402 mg, 1.40 mmol, 35% over 2 steps) as a colorless oil.

**S4**: IR (film) 2942, 2928, 2364, 1738, 1690, 1458, 1366, 1339, 1283, 1240, 1163, 1130, 1057, 1017, 959, 934, 837, 756 488, 451, 436; $^1$H NMR (600 MHz, $CDCl_3$) δ 6.15 (dd, *J* = 18.0, 11.4 Hz, 1H), 5.34 (dd, *J* = 11.4, 1.8 Hz, 1H), 5.22 (dd, J = 18.0, 1.8 Hz, 1H), 2.79 (app td, *J* = 12.0, 4.8 Hz, 1H), 2.04 (s, 3H), 1.89 (ddd, *J* = 13.2, 5.4, 3.6 Hz, 1H), 1.63–1.58 (m, 2H), 1.18 (s, 3H), 1.06 (s, 3H); $^{13}$C NMR (150 MHz, $CDCl_3$) δ 169.7, 145.1, 135.5, 125.6, 119.9, 81.8, 38.1, 36.4, 30.6, 29.2, 26.9, 26.0, 22.4; HRMS (ESI) *m/z* calcd for $C_{13}H_{19}BrNaO_2$ $[M+Na]^+$ 309.0461, found 309.0461.

***(E)-(2-(2-bromo-3,6,6-trimethylcyclohex-2-en-1-ylidene)ethyl)trimethylsilane* (S5)**

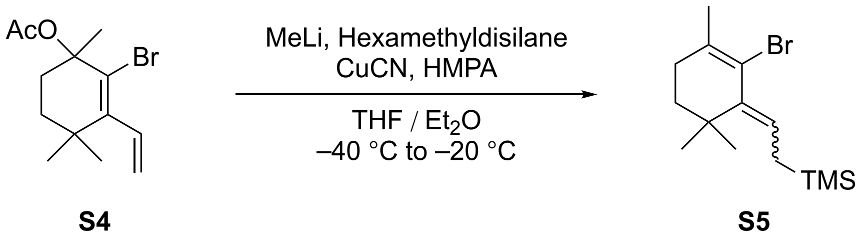


**S4** **S5**

To a solution of hexamethyldisilane (0.55 mL, 2.72 mmol) in $Et_2O$ (5.8 mL) and HMPA (5.8 mL) was added MeLi (3.1 M solution in diethoxymethane, 0.88 mL, 2.72 mmol) dropwise at 0 °C and stirred for 20 min. The solution was added to another flask containing a suspension of CuCN (122 mg, 1.36 mmol) in $Et_2O$ (8.0 mL) and the resulting suspension was stirred for 20 min before it was cooled down to –40 °C. To the suspension of silyl-cuprate was added acetate **S4** (390 mg, 1.36 mmol) in THF (12 mL) dropwise. The reaction was warmed up to –20 °C and stirred for 3 h. The reaction was quenched with saturated

aqueous solution of $NaHCO_3$. The mixture was extracted with hexane and the combined organic layer was washed with brine, dried over $Na_2SO_4$, filtered, and concentrated under reduced pressure. The crude mixture was purified by column chromatography on alumina to obtain allylsilane **S5** (247 mg, 0.82 mmol, ca. 1:0.8 mixture of E/Z-isomers, 60%) as a colorless oil.

**S5**: IR (film) 2965, 2932, 2338, 1464, 1248, 1217, 1144, 1017, 853, 762, 503, 469, 451; $^{1}$H NMR (400 MHz, $CDCl_3$) δ 6.13 (t, *J* = 9.2 Hz, 1H), 5.39 (t, *J* = 9.2 Hz, 0.8H), 2.24–2.16 (m, 3.6H), 2.06 (d, *J* = 9.6 Hz, 1.8H), 1.99 (s, 3H), 1.88 (s, 2.4H), 1.85 (d, *J* = 9.6 Hz, 1.8H), 1.50 (d, *J* = 6.4 Hz, 1.8H), 1.47 (d, *J* = 6.4 Hz, 1.8H), 1.24 (s, 6H), 1.06 (s, 4.8H), 0.06 (s, 9H), 0.01 (s, 7.2H); $^{13}$C NMR (100 MHz, $CDCl_3$) δ 139.9, 137.9, 136.2, 133.7, 128.9, 124.9, 122.4, 115.7, 39.8, 38.4, 38.0, 36.3, 31.7, 30.8, 28.5, 26.9, 26.3, 23.8, 21.7, 21.2, -1.3, -1.3; HRMS (ESI, APCI) peaks were not detectable.

***rac-2-bromo-1-methyl-3-vinylcyclohex-2-en-1-yl acetate*** **(S7)**

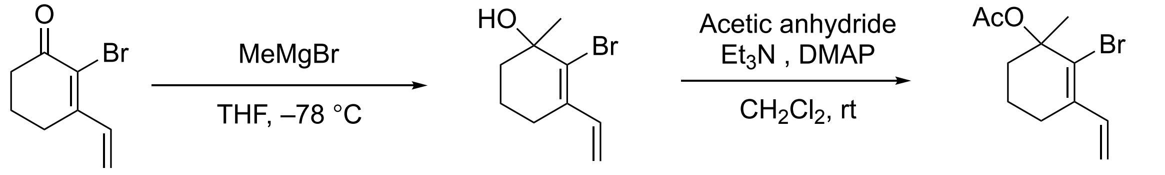


**S1** **S6** **S7**

To a solution of ketone **S1** (697 mg, 3.46 mmol) in THF (4.0 mL) was added MeMgBr (1.0 M solution in THF, 4.20 mL, 4.16 mmol) –78 °C. The mixture was warmed up to room temperature over 1.0 h and stirred for 1.5 h at the temperature. The reaction was quenched with saturated aqueous solution of $NH_4Cl$. The mixture was extracted with $Et_2O$ and the combined organic layer was washed with brine, dried over $Na_2SO_4$, filtered, and concentrated under reduced pressure. The crude mixture was purified by column chromatography on silica gel to obtain alcohol **S6** (483 mg), which contains some impurities. This mixture was dissolved in $CH_2Cl_2$ (4.0 mL) and treated with $Et_3N$ (0.96 mL, 6.92 mmol), $Ac_2O$ (0.66 mL, 6.92 mmol) and DMAP (42.8 mg, 0.35 mmol) at 0 °C. The mixture was warmed up to room temperature and stirred for 36 h. The reaction was quenched with saturated aqueous solution of $NaHCO_3$ at 0 °C. The mixture was extracted with $Et_2O$, and the combined organic layer was washed with brine, dried over $Na_2SO_4$, filtered, and concentrated under reduced pressure. The crude mixture was purified by column chromatography on alumina to obtain acetate **S7** (437 mg, 1.69 mmol, 49% over 2 steps) as a colorless oil.

**S7**: IR (film) 2936, 2872, 1765, 1736, 1622, 1369, 1346, 1242, 1201, 1167, 991, 920, 752, 590, 503, 461; $^{1}$H NMR (600 MHz, $CDCl_3$) δ 6.92 (dd, *J* = 17.4 Hz, 10.8 Hz, 1H), 5.37 (dd, *J* = 17.4 Hz, 1.2 Hz, 1H), 5.26 (dd, *J* = 10.8 Hz, 0.6 Hz, 1H), 2.65 (td, *J* = 12.6 Hz, 3.6 Hz, 1H), 2.34 (app dd, *J* = 7.2 Hz, 4.2 Hz, 2H), 2.04 (s, 3H), 1.99 (ddd, *J* = 13.2 Hz, 6.0 Hz, 3.0 Hz, 1H), 1.93–1.85 (m, 1H), 1.72–1.63 (m, 1H), 1.60 (s, 3H); $^{13}$C NMR (100 MHz, $CDCl_3$) δ 169.6, 137.3, 135.0, 129.0, 117.2, 82.1, 34.4, 27.2, 26.9, 22.3, 19.8; HRMS (ESI) *m/z* calcd for $C_{11}H_{15}BrNaO_2$ $[M+Na]^+$ 281.0148, found 281.0140.

***(E)-(2-(2-bromo-3-methylcyclohex-2-en-1-ylidene)ethyl)trimethylsilane*** **(S8)**

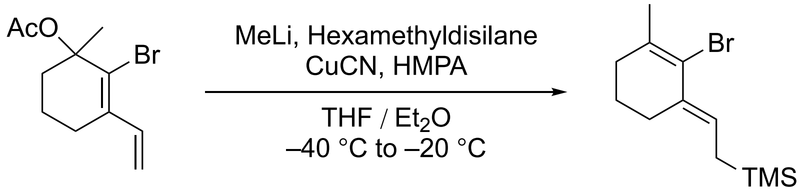


**S7** **S8**

To a solution of hexamethyldisilane (0.69 mL, 3.36 mmol) in $Et_2O$ (6.8 mL) and HMPA (6.8 mL) was added MeLi (3.1 M solution in diethoxymethane, 1.09 mL, 3.38 mmol) dropwise at 0 °C and stirred for 15 min. The solution was added to another flask containing a suspension of CuCN (151 mg, 1.69 mmol) in $Et_2O$ (8.0 mL) and the resulting suspension was stirred for 20 min before it was cooled down to –40 °C. To the suspension of silyl-cuprate was added acetate **S7** (437 mg, 1.69 mmol) in THF (14 mL) dropwise. The reaction was warmed up to –20 °C and stirred for 3 h. The reaction was quenched with saturated aqueous solution of $NaHCO_3$. The mixture was extracted with hexane and the combined organic layer was washed with brine, dried over $Na_2SO_4$, filtered, and concentrated under reduced pressure. the crude mixture was purified by column chromatography on alumina to obtain allylsilane **S8** (257 mg, 0.95 mmol, 57%) as a colorless oil.

**S8**: IR (film) 2953, 1716, 1686, 1609, 1431, 1250, 1144, 976, 845, 758, 692; $^1$H NMR (400 MHz, $CDCl_3$) δ 5.98 (t, *J* = 8.8 Hz, 1H), 2.33 (td, *J* = 6.0, 1.2 Hz, 2H), 2.25 (t, *J* = 6.0 Hz, 2H), 1.96 (s, 3H), 1.73–1.63 (m, 2H), 1.58 (d, *J* = 9.2 Hz, 1H), 0.02 (s, 9H); $^{13}$C NMR (100 MHz, $CDCl_3$) δ 134.0, 130.8, 126.0, 122.6, 34.3, 27.0, 25.3, 22.6, 19.9, -1.5;
HRMS (ESI, APCI) peaks were not detectable.

***(E)-(2-(2-bromospiro[5.5]undec-2-en-1-ylidene)ethyl)trimethylsilane* (S16)**

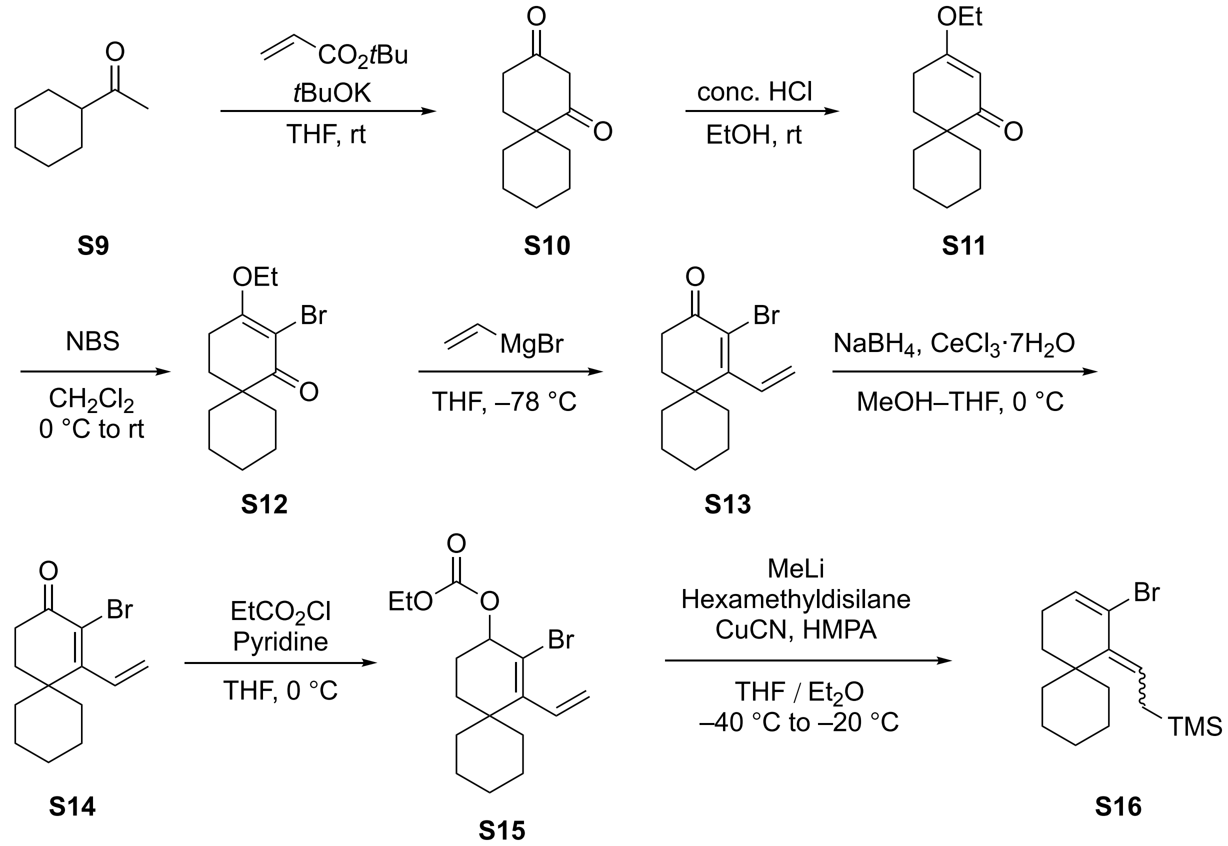


To a solution of cyclohexyl methyl ketone **S9** (1.51 mL, 11.0 mmol) in THF (12 mL) was added *t*BuOK (1.23 g, 11.0 mmol) at room temperature. After 10 min, the mixture was cooled to 0 °C and *tert*-butyl acrylate (1.46 mL, 10.0 mmol) was added. The mixture was warmed to room temperature and stirred for 45 min. The mixture was acidified with 2 M aqueous HCl solution (8.5 mL) and extracted with EtOAc. The organic layer was dried over $Na_2SO_4$, filtered, and concentrated under reduced pressure. The obtained white solid containing **S10** was used for the next reaction without purification.

Obtained **S10** was dissolved in EtOH (20 mL) and conc. HCl (0.5 mL) was added at room temperature. After being stirred for 20 h, the solution was poured into a 0 °C saturated solution of $NaHCO_3$ to quench the reaction. EtOH was removed under reduced pressure and the resulting aqueous mixture was extracted with EtOAc. The combined organic extracts were washed with brine, dried over $Na_2SO_4$, filtered, and concentrated under reduced pressure. The crude mixture was purified by column chromatography on silica gel to obtain vinylogous ester **S11** (1.04 g, contains impurities) which was used for the next reaction.

Vinylogous ester **S11** (1.04 g) was dissolved in $CH_2Cl_2$ (10 mL) and the solution was cooled to 0 °C. To the solution was added NBS (890 mg, 5.0 mmol) portionwise over 40 min. The reaction was quenched with a saturated aqueous solution of $NaHCO_3$ and $Na_2S_2O_3$ at 0 °C. The mixture was extracted with $CH_2Cl_2$, and the combined organic layer was washed with brine, dried over $Na_2SO_4$, filtered, and concentrated

under reduced pressure. The crude mixture was purified by column chromatography on silica gel to obtain vinyl bromide **S12** (1.09 g, contains a small amount of impurity).

To a solution of vinyl bromide **S12** (1.09 g) in THF (8.3 mL) was added vinyl magnesium bromide (1.0 M solution in THF, 5.7 mL, 5.7 mmol) at 0 °C. After being stirred at the same temperature for 3.5 h, the reaction was quenched with a 2 M aqueous HCl at 0 °C. The mixture was extracted with EtOAc, and the combined organic layer was washed with a saturated aqueous solution of $NaHCO_3$ and brine, dried over $Na_2SO_4$, filtered, and concentrated under reduced pressure. The crude mixture was purified by column chromatography on silica gel to obtain enone **S13** (689 mg, 2.56 mmol), which was used for the next reaction immediately since it was somewhat unstable.

To a solution of enone **S13** (689 mg, 2.56 mmol) in THF (6.0 mL) and MeOH (6.0 mL) was added $CeCl_3·7H_2O$ (1.07 g, 2.87 mmol) and $NaBH_4$ (106 mg, 2.81 mmol) sequentially at 0 °C. After being stirred at the same temperature for 2 h, the reaction was quenched with water. MeOH was removed under reduced pressure and the resulting aqueous mixture was extracted with EtOAc. The combined organic extracts were washed with brine, dried over $Na_2SO_4$, filtered, and concentrated under reduced pressure. The crude mixture was purified by column chromatography on silica gel to obtain alcohol **S14** (658 mg, 2.43 mmol).

To a solution of alcohol **S14** (658 mg, 2.43 mmol) in $CH_2Cl_2$ (24 mL) was added pyridine (0.47 mL, 5.83 mmol) and ethyl chloroformate (0.55 mL, 5.83 mmol) at 0 °C. After being stirred at 0 °C for 5 min, the mixture was warmed to room temperature and stirred for 1 h. The reaction was quenched with a saturated aqueous solution of $NaHCO_3$. The mixture was extracted with EtOAc, and the combined organic layer was washed with brine, dried over $Na_2SO_4$, filtered, and concentrated under reduced pressure. The crude mixture was purified by column chromatography on silica gel to obtain carbonate **S15** (726 mg, 2.11 mmol).

To a solution of hexamethyldisilane (0.52 mL, 2.59 mmol) in $Et_2O$ (3.7 mL) and HMPA (3.7 mL) was added MeLi (2.78 M solution in diethoxymethane, 0.67 mL, 1.86 mmol) dropwise at 0 °C and stirred for 15 min. The solution was added to another flask containing a suspension of CuCN (83 mg, 0.93 mmol) in $Et_2O$ (4.65 mL) and the resulting suspension was stirred for 20 min before it was cooled down to –40 °C. To the suspension of silyl-cuprate was added carbonate **S15** (360 mg, 0.87 mmol) in THF (7.4 mL), which was pre-cooled to –40 °C, dropwise. The reaction was warmed up to –20 °C and stirred for 1.5 h. The reaction was quenched with a saturated aqueous solution of $NaHCO_3$. The mixture was extracted with hexane, and the combined organic layer was washed with brine, dried over $Na_2SO_4$, filtered, and concentrated under reduced pressure. The crude mixture was purified by column chromatography on alumina to obtain allylsilane **S16** (176 mg, 0.538 mmol, 62%, 5:1 mixture of isomers) as a colorless oil.

**S16**: IR (film) 3131, 2924, 1636, 1451, 1426, 1246, 1138, 1049, 854, 756, 692; $^1$H NMR (400 MHz, $CDCl_3$) δ 6.15 (t, *J* = 4.2 Hz, 0.2H), 6.10 (t, *J* = 3.6 Hz, 0.2H), 6.00 (t, *J* = 3.6 Hz, 1H), 5.46 (t, *J* = 3.6 Hz), 2.14 (td, *J* = 6.6, 4.2 Hz, 2.2H), 2.07 (d, *J* = 9.6 Hz, 2H), 1.94 (d, *J* = 9.6 Hz, 0.4H), 1.91 (td, *J* = 13.8 Hz, 4.8 Hz, 0.2H), 1.77 (t, *J* = 6.0 Hz, 0.4H), 1.64–1.35 (m, 14H), 0.08 (s, 1.8H), 0.03 (s, 9H); $^{13}$C NMR (150 MHz, $CDCl_3$) δ 138.6, 137.8(minor), 132.3, 130.3(minor), 128.7(minor), 128.0(minor), 125.3, 118.0, 41.0, 40.7(minor),

34.7, 34.3(minor), 34.1(minor), 29.9(minor), 27.1, 26.3(minor), 25.7, 24.4(minor), 22.8, 22.3(minor), 22.2, 21.2. -1.19(major+minor); HRMS (ESI, APCI) peaks were not detectable.

***(E)-(2-(2-bromo-6-methylcyclohex-2-en-1-ylidene)ethyl)trimethylsilane*** **(S20)**

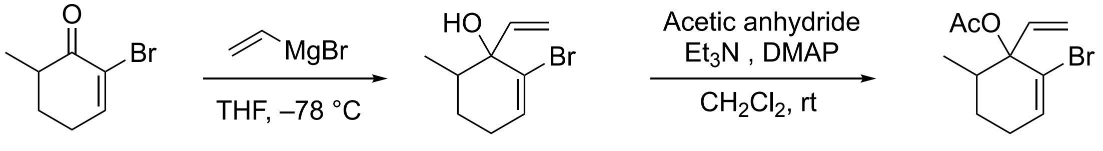


**S17** **S18** **S19**

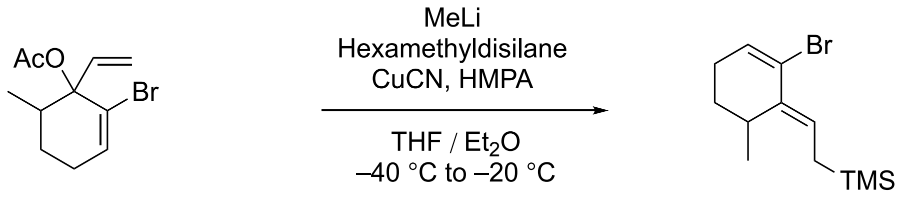


**S19** **S20**

To a solution of vinyl magnesium bromide (1.0 M solution in THF, 10 mL, 10 mmol) diluted with $Et_2O$ (55 mL) was added a solution of bromoenone **S17**[77] (955 mg, 5.05 mmol) in $Et_2O$ (8 mL) at –78 °C dropwise. The mixture was stirred for 30 min, and the reaction was quenched with a saturated aqueous solution of $NH_4Cl$ at the same temperature, which was then warmed to room temperature. The mixture was extracted with $Et_2O$, and the combined organic layer was washed with brine, dried over $Na_2SO_4$, filtered, and concentrated under reduced pressure. The crude mixture was purified by column chromatography on silica gel to obtain alcohol **S18** (700 mg, 3.22 mmol, 64%).

To the solution of alcohol **S18** (700 mg, 3.22 mmol) in $CH_2Cl_2$ (4.3 mL) was added $Et_3N$ (0.63 mL, 5.76 mmol), $Ac_2O$ (0.54 mL, 5.76 mmol) and DMAP (35.4 mg, 0.29 mmol) at 0 °C. The mixture was warmed up to room temperature and stirred for 45 h. The reaction was quenched with saturated aqueous solution of $NaHCO_3$. The mixture was extracted with $CH_2Cl_2$, dried over $Na_2SO_4$, filtered, and concentrated under reduced pressure. The crude mixture was purified by column chromatography on silica gel, which was pre-treated with $Et_3N$, to obtain acetate **S19** (334 mg, 1.29 mmol, 40%).

To a solution of hexamethyldisilane (0.60 mL, 2.99 mmol) in $Et_2O$ (4.2 mL) and HMPA (4.2 mL) was added MeLi (2.78 M solution in diethoxymethane, 0.76 mL, 2.12 mmol) dropwise at 0 °C and stirred for 15 min. The solution was added to another flask containing a suspension of CuCN (95.0 mg, 1.06 mmol) in $Et_2O$ (8.0 mL) and the resulting suspension was stirred for 20 min before it was cooled down to –40 °C. To the suspension of silyl-cuprate was added acetate **S19** (322 mg, 1.24 mmol) in THF (8.5 mL) which was pre-cooled to –40 °C dropwise. The reaction was warmed up to –20 °C and stirred for 2 h. The reaction was quenched with a saturated aqueous solution of $NaHCO_3$. The mixture was extracted with hexane and the combined organic layer was washed with brine, dried over $Na_2SO_4$, filtered, and concentrated under reduced pressure. the crude mixture was purified by column chromatography on alumina to obtain allylsilane **S20** (145 mg, 0.531 mmol, 43%) as a colorless oil.

**S20**: IR (film) 3416, 2874, 2361, 1730, 1632, 1248, 854, 837, 777, 691, 465, 413; $^1$H NMR (400 MHz, $CDCl_3$) δ 6.07 (dd, *J* =6.0, 2.8 Hz, 1H), 5.91 (t, *J* = 9.2 Hz, 1H), 2.98–2.89 (m, 1H), 2.40–2.28 (m, 1H), 2.11 (dtd, *J* = 18.0, 6.0, 2.0 Hz, 1H), 1.73–1.50 (m, 4H), 1.00 (d, *J* = 7.2 Hz 3H), 0.03 (s, 9H, major); $^{13}$C NMR (150 MHz, $CDCl_3$) δ 134.7, 128.3, 128.0, 124.2, 30.1, 28.0, 24.1, 19.2, 17.7, -1.5; HRMS (ESI, APCI) peaks were not detectable.

***rac-2-bromo-3-(dimethyl(phenyl)silyl)-3-methylcyclohex-1-en-1-yl trifluoromethanesulfonate* (S22)**

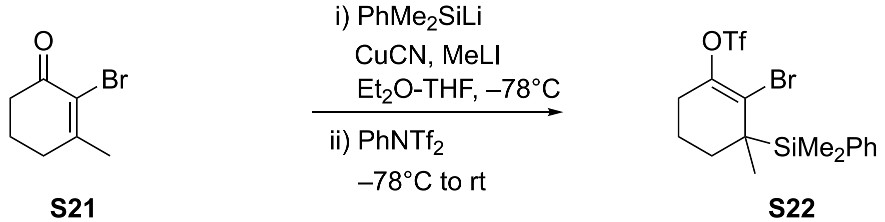


**S21** **S22**

Silyl lithium preparation: Lithium ribbon (314 mg, 45.2 mmol after washing) was added to a flask, and paraffin oil was removed by washing with hexane. The flask was then filled with THF (30 mL). PhMe2SiCl (1.65 mL, 9.96 mmol) was added to this mixture at 0 °C, and after 5 min, the mixture was warmed to room temperature and stirred for 15 h. The resulting brown suspension was diluted with THF (20 mL) and used as a silyl lithium solution for the following reaction.

CuCN (985 mg, 11.0 mmol) was added to a flask and suspended by adding $Et_2O$ (13 mL). The suspension was cooled to –78 °C and added MeLi (3.1 M solution in diethoxymethane, 3.2 mL, 10 mmol).[79] The dryice-acetone bath was then removed, and the mixture was warmed to 0 °C using the ice-water bath. After being stirred for 10 min, the mixture, which became a bright yellow suspension, was re-cooled to –78 °C, and silyl lithium solution was added dropwise via cannula. To the cuprate suspension was then added a solution of bromo enone **S21**[78] (1.04 g, 5.52 mmol) in $Et_2O$ (10 mL) and stirred for 100 min. To the mixture was added a solution of $PhNTf_2$ (3.57 g, 10 mmol) in THF (20 mL) at the same temperature, and the resulting mixture was gradually warmed up to room temperature over 29 h. The reaction was quenched by adding a saturated aqueous solution of NH4Cl at 0 °C. The mixture was extracted with EtOAc, and combined organic extracts were washed with a saturated aqueous solution of $NaHCO_3$ and brine. The organic layer was dried over $Na_2SO_4$, filtered, and concentrated under reduced pressure. the crude mixture was purified by column chromatography on short pad of silica gel, followed by on alumina to obtain vinyl triflate **S22** (1.37 g, 2.99 mmol, 54%) as a colorless oil.

**S22**: IR (film) 2951, 2868, 1645, 1456, 1422, 1375, 1341, 1250, 1209, 1140, 1013, 974, 891, 814, 737, 702; $^1$H NMR (600 MHz, $CDCl_3$) δ 7.55-7.53 (m, 2H), 7.40-7.34 (m, 3H), 2.30 (app t, J = 6.6 Hz, 2H), 1.73 (ddd, J = 13.2, 7.2, 6.0 Hz, 1H), 1.68-1.63 (m, 2H), 1.35-1.31 (m, 1H), 1.33 (s, 3H), 0.51 (s, 3H), 0.44 (s, 3H); $^{13}$C NMR (150 MHz, $CDCl_3$) δ 144.2, 136.7, 134.7, 129.5, 127.9, 127.0, 118.4 (q, *J* = 318.8 Hz), 34.6, 33.3, 29.6, 23.0, 18.6, -3.1, -4.1; HRMS (ESI, APCI) peaks were not detectable.

***rac-(2-bromo-1-methyl-3-vinylcyclohex-2-en-1-yl)dimethyl(phenyl)silane* (34)**

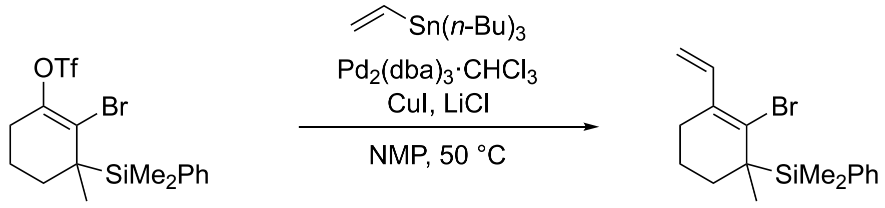


**S22** **34**

To a solution of vinyl triflate **S22** (236 mg, 0.516 mmol) in NMP (2.5 mL) was added tributyl(vinyl)tin (175 µL, 0.602 mmol), CuI (6.0 mg, 0.0315 mmol), LiCl (63.6 mg, 1.50 mmol) and $Pd_2(dba)_3·CHCl_3$ (25.9 mg, 0.025 mmol) at room temperature.[80] The flask was evacuated and backfilled with nitrogen. The mixture was then stirred at 50 °C for 80 min. After cooling down to room temperature, the mixture was poured into a biphasic mixture of hexane-EtOAc-water (ca. 10:1:5). The organic layer was separated, and the aqueous layer was extracted with hexane-EtOAc mixture (ca. 10:1). The combined organic layers were washed with water and brine, dried over $Na_2SO_4$, filtered, and concentrated under reduced pressure. the crude mixture was purified by column chromatography on alumina to obtain diene **34** (140 mg, 0.418 mmol, 81%) as a colorless oil.

**34**: IR (film) 2940, 2864, 1616, 1458, 1427, 1250, 1217, 1109, 939, 905, 816, 758, 702; $^{1}$H NMR (600 MHz, $CDCl_3$) δ 7.56-7.53 (m, 2H), 7.36-7.31 (m, 3H), 6.99 (dd, J = 17.4, 10.8 Hz, 1H), 5.18 (dd, J = 17.4, 1.8 Hz, 1H), 5.08 (dd, J = 10.8, 1.2 Hz, 1H), 2.27 (dt, J = 16.8, 6.0 Hz, 1H), 2.06 (dt, J = 16.8, 6.6 Hz, 1H), 1.74 (ddd, J = 12.0, 7.2, 4.8 Hz, 1H), 1.58-1.54 (m, 2H), 1.38-1.33 (m, 1H), 1.36 (s, 3H), 0.49 (s, 3H), 0.40 (s, 3H); $^{13}$C NMR (150 MHz, $CDCl_3$) δ 138.4, 138.1, 136.7, 134.7, 131.4, 129.1, 127.6, 114.1, 36.3, 35.4, 27.7, 24.2, 18.7, -2.5, -3.4; HRMS (ESI, APCI) peaks were not detectable.

A deuterium labeled **34** was also obtained with the same procedure. Vinyl triflate **S22** (228 mg, 0.498 mmol) was converted to diene **34-d₁** (115.6 mg, 0.344 mmol, 69%) using deuterium-labeled tributyl(vinyl)tin[82].

***Rac-(E)-(2-bromo-3-(2-cyclopropylvinyl)-1-methylcyclohex-2-en-1-yl)dimethyl(phenyl)silane* (37)**

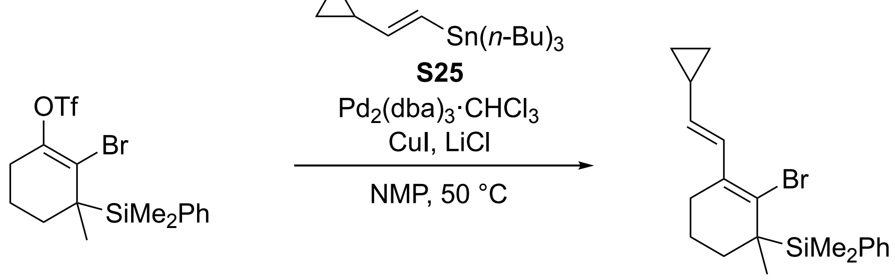


**S22** **37**

To a solution of vinyl triflate **S22** (248 mg, 0.542 mmol) in NMP (2.5 mL) was added vinyltin **S25**[81] (582 mg, 1.6 mmol, *E*-isomer: *Z*-isomer=1.4:1), CuI (4.8 mg, 0.0252 mmol), LiCl (63.6 mg, 1.50 mmol) and

$Pd_2(dba)_3{\cdot}CHCl_3$ (25.9 mg, 0.025 mmol) at room temperature. The flask was evacuated and backfilled with nitrogen. The mixture was then stirred at room temperature for 30 min and at 50 °C for 3 h. After cooling down to room temperature, the mixture was poured into a biphasic mixture of hexane-EtOAc-water (ca. 10:1:5). The organic layer was separated, and the aqueous layer was extracted with hexane-EtOAc mixture (ca. 10:1). The combined organic layers were washed with water and brine, dried over $Na_2SO_4$, filtered, and concentrated under reduced pressure. the crude mixture was purified by column chromatography on alumina to obtain diene **37** (33.9 mg, 0.0903 mmol, 17%, *E*-isomer only) as a colorless oil.

**37**: IR (film) 3275, 1647, 1420, 1250, 1217, 1142, 1107, 816, 760, 702; $^{1}$H NMR (600 MHz, $CDCl_3$) δ 7.55-7.52 (m, 2H), 7.35-7.30 (m, 3H), 6.79 (d, *J* = 15.6 Hz, 1H), 5.16 (dd, *J* = 15.6, 9.0 Hz, 1H), 2.20 (dt, *J* = 16.2, 6.0 Hz, 1H), 2.02 (dt, *J* = 16.2, 7.2 Hz, 1H), 1.73-1.68 (m, 1H), 1.55-1.50 (m, 3H), 1.33-1.30 (m, 4H), 0.79-0.76 (m, 2H), 0.48 (s, 3H), 0.43-0.40 (m, 2H), 0.38 (s, 3H); $^{13}$C NMR (150 MHz, $CDCl_3$) δ 138.4, 135.4, 134.7, 133.5, 130.8, 129.4, 129.0, 127.6, 36.3, 35.1, 28.3, 24.3, 18.8, 15.0, 7.7, 7.6, -2.4, -3.5; HRMS (ESI, APCI) peaks were not detectable.

***rac-2-bromo-3-(dimethyl(phenyl)silyl)cyclohex-1-en-1-yl trifluoromethanesulfonate*** **(S24)**

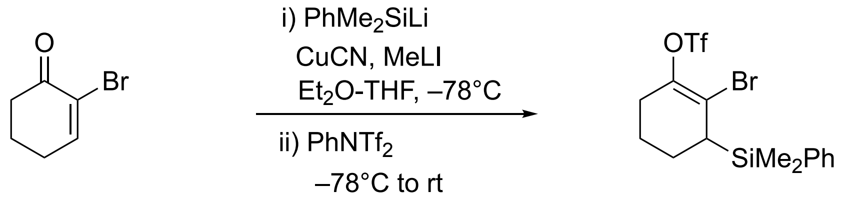


**S23** **S24**

Silyl lithium preparation: Lithium ribbon (340 mg, 49.0 mmol after washing) was added to a flask, and paraffin oil was removed by washing with hexane. The flask was then filled with THF (35 mL). $PhMe_2SiCl$ (1.65 mL, 9.96 mmol) was added to this mixture at 0 °C, and after 5 min, the mixture was warmed to room temperature and stirred for 15 h. The resulting brown suspension was used as a silyl lithium solution for the following reaction.

CuCN (990 mg, 11.1 mmol) was added to a flask and suspended by adding $Et_2O$ (13 mL). The suspension was cooled to –78 °C and added MeLi (2.8 M solution in diethoxymethane, 3.6 mL, 10 mmol). The dry ice-acetone bath was then removed, and the mixture was warmed to 0 °C using the ice-water bath. After being stirred for 15 min, the mixture, which became a bright yellow suspension, was re-cooled to –78 °C, and silyl lithium solution was added dropwise via cannula. To the cuprate suspension was then added a solution of bromo enone **S23** (1.31 g, 7.47 mmol) in THF (10 mL) and stirred for 2 h. To the mixture was added a solution of $PhNTf_2$ (3.57 g, 10 mmol) in THF (20 mL) at the same temperature, and the resulting mixture was gradually warmed up to room temperature over 17 h. The reaction was quenched by adding a saturated aqueous solution of NH4Cl at 0 °C. The mixture was extracted with EtOAc, and combined organic extracts were washed with a saturated aqueous solution of $NaHCO_3$ and brine. The organic layer was dried over $Na_2SO_4$, filtered, and concentrated under reduced pressure. the

crude mixture was purified by column chromatography on a short pad of silica gel, followed by alumina to obtain vinyl triflate **S24** (2.68 g, 6.03 mmol, 81%) as a colorless oil.

**S24**: IR (film) 2955, 2864, 1655, 1589, 1420, 1339, 1204, 1140, 1057, 1026, 988, 963, 891, 702; $^1$H NMR (600 MHz, $CDCl_3$) δ 7.53-7.50 (m, 2H), 7.40-7.35 (m, 3H), 2.43-2.33 (m, 2H), 2.29 (dtd, *J* = 16.8, 6.0, 2.4 Hz, 1H), 1.77-1.71 (m, 1H), 1.71-1.57 (m, 3H), 0.51 (s, 3H), 0.45 (s, 3H); $^{13}$C NMR (150 MHz, $CDCl_3$) δ 143.3, 137.3, 134.0, 129.5, 128.1, 119.5, 118.5(q, *J* = 318.8 Hz), 34.9, 29.1, 26.7, 21.8, -1.9, -3.1; HRMS (ESI, APCI) peaks were not detectable.

***rac-(2-bromo-3-(prop-1-en-2-yl)cyclohex-2-en-1-yl)dimethyl(phenyl)silane*** **(35)**

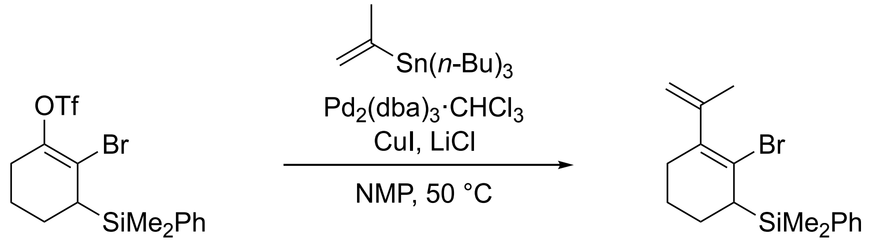


**S24** **35**

To a solution of vinyl triflate **S24** (443 mg, 0.999 mmol) in NMP (5.0 mL) was added tributyl(isopropenyl)tin (364 mg, 1.1 mmol), CuI (14.2 mg, 0.0747 mmol), LiCl (121 mg, 2.85 mmol) and $Pd_2(dba)_3{\cdot}CHCl_3$ (49.0 mg, 0.0473 mmol) at room temperature. The flask was evacuated and backfilled with nitrogen. The mixture was then stirred at 50 °C for 4 h. After cooling down to room temperature, the mixture was poured into a biphasic mixture of hexane-EtOAc-water (ca. 10:1:5). The organic layer was separated, and the aqueous layer was extracted with hexane-EtOAc mixture (ca. 10:1). The combined organic layers were washed with water and brine, dried over $Na_2SO_4$, filtered, and concentrated under reduced pressure. the crude mixture was purified by column chromatography on alumina to obtain diene **35** (146 mg, 0.435 mmol, 44%) as a colorless oil.

**35**: IR (film) 2936, 2860, 1647, 1624, 1427, 1370, 1250, 1217, 1111, 963, 897, 833, 818, 758, 702; $^1$H NMR (600 MHz, $CDCl_3$) δ 7.57-7.55 (m, 2H), 7.36-7.34 (m, 3H), 4.91 (t, J = 1.8 Hz, 1H), 4.64 (t, J = 1.2 Hz, 1H), 2.36-2.33 (m, 1H), 2.19-2.12 (m, 1H), 1.98 (dtd, J = 16.8, 5.4, 1.8 Hz, 1H), 1.80 (s, 3H), 1.80-1.72 (m, 1H), 1.67-1.47 (m, 3H), 0.48 (s, 3H), 0.45 (s, 3H); $^{13}$C NMR (150 MHz, $CDCl_3$) δ 148.1, 138.6, 137.3, 134.2, 129.1, 127.7, 119.9, 113.1, 36.1, 31.7, 27.8, 21.6, 21.4, -1.7, -2.2; HRMS (ESI, APCI) peaks were not detectable.

# Procedures for CsF mediated Strain release Diels-Alder reaction

## General procedure for the Diels-Alder reaction

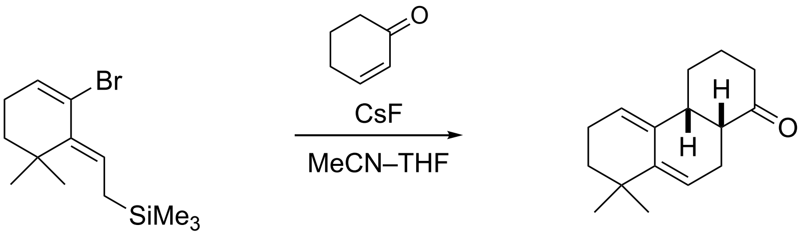


**15** **16**

To a suspension of CsF (76.0 mg, 0.500 mmol) in MeCN (0.2 mL) was added 2-cyclohexene-1-one (50 µL, 0.520 mmol) followed by a solution of allylsilane **15** (29.0 mg, 0.100 mmol) in MeCN–THF (1:1, 0.8 mL) at room temperature, and the mixture was stirred for 22 h. The reaction was diluted with water and the mixture was extracted with $Et_2O$. Combined organic extracts were washed with brine, dried over $Na_2SO_4$, filtered, and concentrated under reduced pressure. the crude mixture was purified by column chromatography on silica gel to obtain Diels-Alder adduct **16** (13.4 mg, 0.058 mmol, 58%) as a colorless oil.

***rac-(4aR,10aR)-8,8-dimethyl-3,4,4a,6,7,8,10,10a-octahydrophenanthren-1(2H)-one*** **(16)**

**16**: IR (film) 3412, 2940, 2365, 1684, 1458, 1364, 1256, 1046, 843, 754, 569, 543, 499, 470, 457 cm$^{-1}$; $^{1}$H NMR ($CDCl_3$, 600 MHz) δ 5.53–1.51 (m, 1H), 5.50 (br s, 1H), 2.66–2.58 (m, 2H), 2.51 (ddd, *J* = 15.0, 12.6, 7.2 Hz, 1H), 2.41 (dddd, *J* = 18.0, 10.2, 3.0, 1.2 Hz, 1H), 2.27–2.19 (m, 2H), 2.16–2.12 (m, 2H), 2.06–2.01 (m, 1H), 1.82 (dtd, *J* = 13.8, 11.4, 3.6 Hz, 1H), 1.76–1.68 (m, 1H), 1.58–1.83 (m, 1H), 1.46 (ddd, *J* = 12.6, 7.8, 6.6 Hz, 1H), 1.40 (dt, *J* = 13.2, 5.4 Hz, 1H), 1.03 (s, 3H), 0.98 (s, 3H); $^{13}$C NMR ($CDCl^3$, 150 MHz) δ 214.2, 141.6, 134.2, 123.4, 115.5, 49.6, 43.8, 37.9, 36.8, 33.4, 28.1, 27.8, 27.3, 25.6, 25.1, 23.1; HRMS (ESI) *m/z* calcd for $C_{16}H_{22}NaO$ $[M+Na]^+$ 253.1568, found 253.1572

**rac-(3aR,9bR)-6,6-dimethyl-1,2,3a,4,6,7,8,9b-octahydro-3H-cyclopenta[a]naphthalen-3-one (18)**

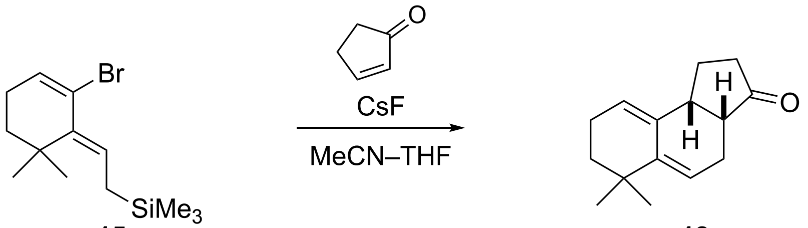


**15** **18**

Following the general procedure, allylsilane **15** (99.7 mg, 0.350 mmol) was converted to the corresponding cycloadduct using 2-cyclopentene-1-one as a dienophile. Purification of the crude mixture by flash chromatography on silica gel gave **18** (30.5 mg, 0.141 mmol, 40%) as a colorless oil.

**18**: IR (film) 3468, 2962, 2916, 1740, 1647, 1464, 1431, 1404, 1361, 1215, 1140, 1064, 1049, 754, 667; $^{1}$H NMR (600MHz, $CDCl_3$) $\delta$ 5.66 (br t, $J$ = 3.6 Hz, 1H), 5.55 (br t, $J$ = 3.6 Hz, 1H), 2.99 (app q, $J$ = 6.6 Hz, 1H), 2.36 (app q, $J$ = 6.6 Hz, 1H), 2.29–2.23 (m, 3H), 2.20 (t, $J$ = 7.2 Hz, 1H), 2.19–2.14 (m, 2H), 2.07–2.00 (m, 1H), 2.00–1.94 (m, 1H), 1.48–1.38 (m, 2H), 1.03 (s, 3H), 0.99 (s, 3H); $^{13}$C NMR (150 MHz, $CDCl_3$) δ 220.7, 142.3, 131.8, 123.9, 116.6, 46.8, 40.5, 36.8, 36.4, 33.6, 28.6, 27.9, 27.6, 23.1, 22.4; HRMS (ESI) *m/z* calcd for $C_{15}H_{20}NaO$ $[M+Na]^+$ 239.1406, found 239.1409;

**rac-(6aR,11aR)-4,4-dimethyl-2,3,4,6,6a,8,9,10,11,11a-decahydro-7H-cyclohepta[a]naphthalen-7-one (19)**

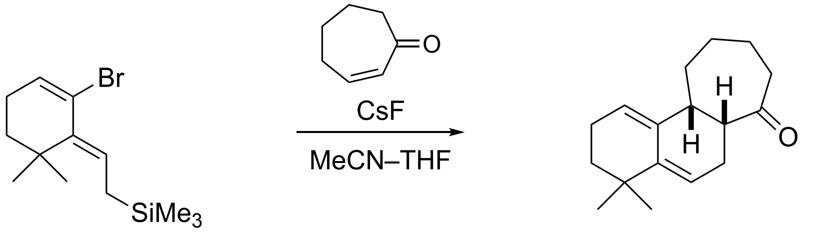


**15** **19**

Following the general procedure, allylsilane **15** (54.2 mg, 0.189 mmol) was converted to the corresponding cycloadduct using 2-cycloheptene-1-one as a dienophile. Purification of the crude mixture by flash chromatography on silica gel gave **19** (19.8 mg, 0.0812 mmol, 43%) as a colorless oil.

**19**: IR (film) 2931, 2348, 1699, 1508, 1262, 795, 536, 524, 519, 503, 494, 475, 461, 453, 442, 424, 414 cm$^{-1}$; $^{1}$H NMR ($CDCl_3$, 400 MHz) δ 5.55–5.51 (m, 1H), 5.50 (br t, $J$ = 4.0 Hz, 1H), 2.79–2.70 (m, 2H), 2.64–2.55 (m, 2H), 2.50–2.42 (m, 1H), 2.24 (app ddq, $J$ = 19.6, 12.4, 1.2 Hz, 1H), 2.17–2.10 (m, 2H), 1.95–1.83 (m, 2H), 1.63–1.54 (m, 2H), 1.54–1.34 (m, 4H), 1.06 (s, 3H), 0.94 (s, 3H); $^{13}$C NMR ($CDCl_3$, 100 MHz) δ

216.2, 141.6, 138.4, 122.0, 117.0, 50.2, 43.2, 42.5, 36.9, 33.2, 33.1, 29.6, 28.3, 26.7, 25.7, 25.1, 23.1 ; HRMS (ESI) *m/z* calcd for $C_{17}H_{24}NaO$ $[M+Na]^+$ 267.1725, found 267.1720

**_rac_- (4a*S*,10a*R*)-8,8,10a-trimethyl-3,4,4a,6,7,8,10,10a-octahydrophenanthren-1(2*H*)-one (20)**

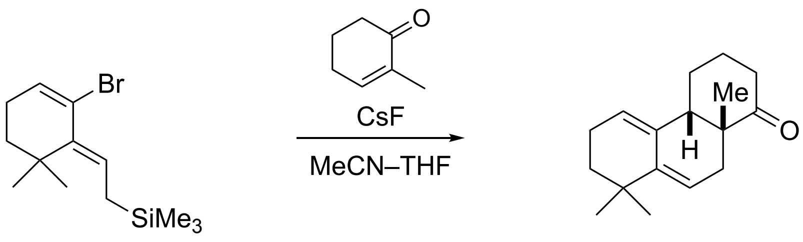


**15** **20**

Following the general procedure, allylsilane **15** (51.0 mg, 0.177 mmol) was converted to the corresponding cycloadduct using 2-methyl-2-cyclohexene-1-one as a dienophile. Purification of the crude mixture by flash chromatography on silica gel gave **20** (19.5 mg, 0.0797 mmol, 45%) as a colorless oil.

**20**: IR (film) 2960, 2935, 2369, 1705, 1458, 1374, 1258, 1094, 924, 754, 744, 520, 503, 493, 467, 457, 439, 4344, 426 $cm^{-1}$;
$^1$H NMR ($CDCl_3$, 600 MHz) δ 5.49 (br t, *J* = 4.2 Hz, 1H), 5.43–5.41 (m, 1H), 2.65 (ddd, *J* = 15.0, 13.8, 6.6 Hz, 1H), 2.60 (br d, *J* = 18 Hz, 1H), 2.29–2.25 (m, 1H), 2.16–2.09 (m, 3H), 2.01–1.97 (m, 1H), 1.85 (dd, *J* = 18.0, 5.4 Hz, 1H), 1.78–1.64 (m, 2H), 1.55–1.50 (m, 1H), 1.46 (ddd, *J* = 12.6, 8.4, 6.6 Hz,1H), 1.41 (app dt, *J* =13.2, 5.4 Hz, 1H), 1.07 (s, 3H), 1.01 (s, 3H), 1.00 (s, 3H); $^{13}$C NMR ($CDCl_3$, 100 MHz) δ 215.5, 140.7, 134.4, 124.3, 113.9, 51.1, 48.4, 37.7, 37.0, 33.3, 32.9, 29.9, 28.2, 27.1, 25.6, 23.2, 20.2;
HRMS (ESI) *m/z* calcd for $C_{17}H_{24}NaO$ $[M+Na]^+$ 267.1725, found 267.1706.

**_rac_-(4a*R*,10a*R*)-4-(benzyloxy)-8,8-dimethyl-3,4,4a,6,7,8,10,10a-octahydrophenanthren-1(2*H*)-one (22)**

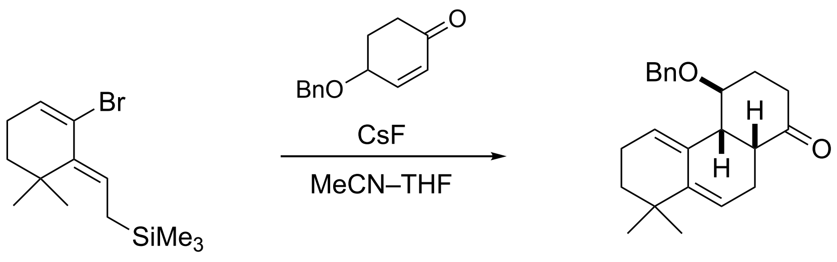


**15** **22**

Following the general procedure, allylsilane **15** (56.8 mg, 0.198 mmol) was converted to the corresponding cycloadduct using 4-benzyloxy-2-cyclohexene-1-one as a dienophile. Purification of the crude mixture by flash chromatography on silica gel gave **22** (29.7 mg, 0.088 mmol, 44%) as a colorless oil.

The same reaction was also conducted using allylsilane **15** (301 mg, 1.03 mmol) to obtain **22** (149 mg, 0.443 mmol, 43%)

**22**: IR (film) 3036, 2926, 1717, 1497, 1454, 1362, 1248, 1098, 1069, 1028, 841, 802, 750, 735, 696; $^1$H NMR (600 MHz, $C_6D_6$) δ 7.31 (d, *J* = 7.8 Hz, 2H), 7.22 (t, *J* = 7.8 Hz, 2H), 7.13 (br t, *J* = 7.2 Hz, 1H), 5.51 (app t, *J* = 3.6 Hz, 1H), 5.34 (app t, *J* = 3.6 Hz, 1H), 4.34 (d, *J* = 12.0 Hz, 1H), 4.27 (d, *J* = 12.0 Hz, 1H), 3.60 (app td, *J* = 6.6, 3.6 Hz, 1H), 2.94 (app q, *J* = 6.0 Hz, 1H), 2.83 (app t, *J* = 6.0 Hz, 1H), 2.41 (ddd, *J* = 18.0, 6.6, 4.2 Hz, 1H), 2.35–2.28 (m, 1H), 2.16 (ddd, *J* = 15.0, 7.8, 5.4 Hz, 1H), 2.06–1.86 (m, 4H), 1.66–1.59 (m, 1H), 1.40–1.29 (m, 2H), 1.02 (s, 3H), 1.01 (s, 3H); $^{13}$C NMR (150 MHz, $CDCl_3$) δ 212.2, 141.6, 138.8, 130.4, 128.5, 127.7, 127.6, 125.1, 116.8, 74.2, 71.7, 47.2, 46.5, 36.6, 36.1, 33.6, 29.0, 28.2, 27.6, 25.5, 23.3; HRMS (ESI) *m/z* calcd for $C_{23}H_{28}NaO_2$ $[M+Na]^+$ 359.1982, found 359.1979.

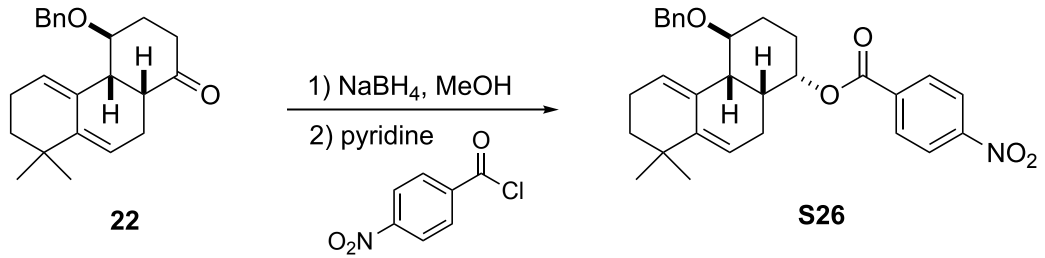


22 S26

Ketone **22** (7.0 mg, 0.021 mmol) in MeOH (0.5 mL) was treated with $NaBH_4$ (2.0 mg, 0.031 mmol) at 0°C to obtain alcohol 3.7 mg (0.011 mmol, 52%).

To the alcohol (28 mg, 0.083 mmol) was dissolved in THF (1.0 mL) and treated with $Et_3N$ (0.04 mL, 0.25 mmol), 4-nitrobenzoyl chloride (32.0 mg, 0.17 mmol), and DMAP (5.0 mg) at room temperature to obtain nitrobenzoate **S26** (40.0 mg, quant.) as a pale yellow solid.

**S26**: IR (film) 2909, 1719, 1530, 1352, 1279, 1215, 1103, 754, 509; $^1$H NMR (600 MHz, $CDCl_3$) δ 8.31–8.26 (m, 2H), 8.20–8.17 (m, 2H), 7.34–7.25 (m, 5H), 5.66 (br t, *J* = 3.6 Hz, 1H), 5.61–5.59 (m, 1H), 5.23 (dt, *J* = 12.0, 4.8 Hz, 1H), 4.49 (d, *J* = 11.4 Hz, 1H), 4.41 (d, *J* = 11.4 Hz, 1H), 3.33 (app td, *J* = 10.8, 4.2 Hz, 1H), 2.58 (app sextet, *J* = 5.4 Hz, 1H), 2.41 (dd, *J* = 10.2, 4.2 Hz, 1H), 2.34 (dd, *J* = 18.0, 12.0 Hz, 1H), 2.28–2.14 (m, 4H), 2.06–2.00 (m, 1H), 1.94–1.86 (m, 1H), 1.55–1.46 (m, 2H), 1.46–1.41 (m, 1H), 1.09 (s, 3H), 1.03 (s, 3H); $^{13}$C NMR (150 MHz, $CDCl_3$) δ 164.1, 150.6, 142.0, 139.1, 136.2, 131.6, 130.8, 128.4, 127.6, 126.6, 123.7, 116.3, 75.9, 74.7, 72.4, 48.4, 37.2, 37.1, 33.6, 29.8, 28.7, 27.2, 24.9, 23.3, 22.9; HRMS (ESI) *m/z* calcd for $C_{30}H_{33}NNaO_5$ $[M+Na]^+$ 510.2251, found 510.2230.

***rac*-ethyl 5,5-dimethyl-1,2,3,5,6,7-hexahydronaphthalene-2-carboxylate (25)**

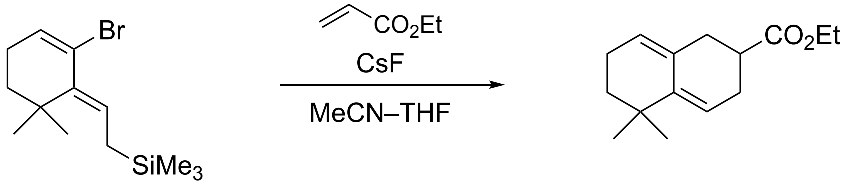


**15** **23**

Following the general procedure, allylsilane **15** (36.4 mg, 0.126 mmol) was converted to the corresponding cycloadduct using ethyl acrylate as a dienophile. Purification of the crude mixture by flash chromatography on silica gel gave **23** (11.4 mg, 0.0486 mmol, 39%) as a colorless oil.

**23**: IR (film) 2949, 2913, 1734, 1453, 1373, 1310, 1250, 1167, 1069, 1030, 862, 802, 525, 500, 432 $cm^{-1}$; $^1$H NMR ($CDCl_3$, 600 MHz) δ 5.58 (br s, 1H), 5.46 (br s, 1H), 4.16–4.10 (m, 2H), 2.62–2.55 (m, 1H), 2.52 (dd, *J* = 14.4, 3.0 Hz, 1H), 2.42–2.31 (m, 3H), 2.12 (br s, 2H), 1.44 (ddd, *J* = 13.2, 7.8, 6.6 Hz, 1H), 1.39 (app dt, *J* = 13.2, 5.4 Hz, 1H) ,1.25 (t, *J* = 7.2 Hz, 3H), 1.04 (s, 3H), 1.00 (s, 3H); $^{13}$C NMR ($CDCl_3$, 150 MHz) δ 175.6, 142.7, 131.3, 123.0, 117.7, 60.4, 40.3, 37.0, 34.3, 33.4, 28.9, 28.3, 27.4, 23.0, 14.4; HRMS (ESI) *m/z* calcd for $C_{15}H_{22}NaO_2$ $[M+Na]^+$ 257.1517, found 257.1521

***rac*-methyl (1*R*,2*S*)-1,5,5-trimethyl-1,2,3,5,6,7-hexahydronaphthalene-2-carboxylate (26)**

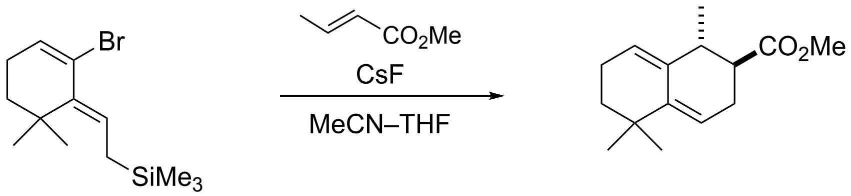


**15** **24**

Following the general procedure, allylsilane **15** (34.1 mg, 0.118 mmol) was converted to the corresponding cycloadduct using ethyl crotonate as a dienophile. Purification of the crude mixture by flash chromatography on silica gel gave **24** (13.7 mg, 0.0585 mmol, 50%) as a colorless oil.

**24**: IR (film) 2951, 2918, 2384, 2373, 1736, 1453, 1362, 1263, 1248, 1209, 1161, 1030, 802, 542, 435 $cm^{-1}$;

$^1$H NMR ($CDCl_3$, 400 MHz) δ 5.55–5.49 (m, 2H), 3.66 (s, 3H), 2.65–2.56 (m, 1H), 2.53–2.45 (m, 1H), 2.43–2.37 (m, 1H), 2.37–2.29 (m, 1H), 2.17–2.11 (m, 2H), 1.41 (t, *J* = 6.4 Hz, 2H), 1.06 (d, *J* = 6.8 Hz, 3H), 1.01 (s, 3H), 1.00 (s, 3H); $^{13}$C NMR ($CDCl_3$, 100 MHz) δ 176.1, 141.8, 135.2, 121.7, 116.1, 51.7, 46.4, 36.8, 36.2, 33.4, 27.9, 27.5, 26.9, 23.2, 20.0, 18.2; HRMS (ESI) *m/z* calcd for $C_{15}H_{22}NaO_2$ $[M+Na]^+$ , 257.1517 found 257.1494

***rac*-1,1-dimethyl-6-phenyl-1,2,3,5,6,7-hexahydronaphthalene (25)**

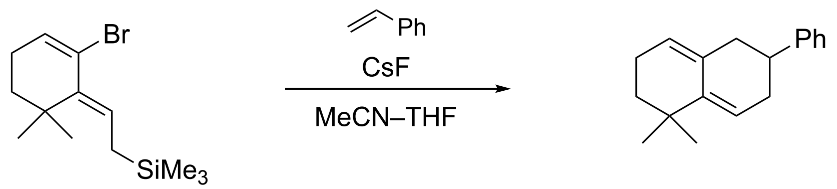


**15** **25**

Following the general procedure, allylsilane **15** (66.6 mg, 0.230 mmol) was converted to the corresponding cycloadduct using ethyl acrylate as a dienophile. The NMR yield using 1,1,2-trichloroethylene as an internal standard resulted in 36% yield along with 28% yield of [2+2]-cycloaddition. Purification of the crude mixture by flash chromatography on silica gel gave **25** (15.0 mg, 0.0629 mmol, 27%) as a colorless oil.

**25**: IR (film) 2959, 2918, 2903, 2895, 1493, 1215, 760, 700, 496, 478, 469, 459, 453, 446, 440, 426, 408 $cm^{-1}$;
$^{1}$H NMR ($CDCl_3$, 600 MHz) δ 7.33–7.29 (m, 2H), 7.25–7.19 (m, 3H), 5.71–7.68 (m, 1H), 5.45 (br s, 1H), 2.90–2.83 (m, 1H), 2.50–2.46 (m, 2H), 2.43 (dt, *J* = 18.0, 5.4 Hz, 1H), 2.31 (dd, *J* = 18.0, 10.8 Hz, 1H), 2.17–2.13 (m, 2H), 1.51 (ddd, *J* = 13.2, 7.8, 7.2 Hz, 1H), 1.44 (dt, *J* = 12.6, 5.4 Hz, 1H), 1.10 (s, 3H), 1.05 (s, 3H); $^{13}$C NMR ($CDCl_3$, 150 MHz) δ 146.8, 142.7, 133.3, 128.5, 127.0, 126.2, 122.3, 119.4, 41.2, 39.2, 37.2, 34.7, 33.4, 28.5, 27.4, 23.0;
HRMS (ESI) *m/z* calcd for $C_{18}H_{22}Na$ $[M+Na]^+$ 261.1619, found 261.1628

### *rac*-ethyl 8-methyl-1,2,3,5,6,7-hexahydronaphthalene-2-carboxylate (32)

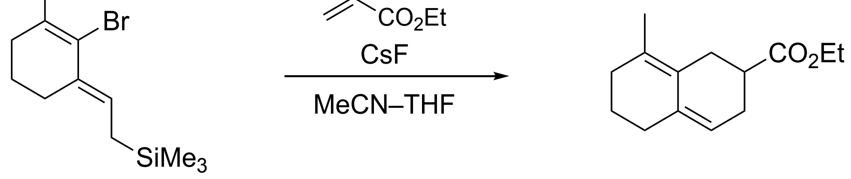


**S8** **32**

Following the general procedure, allylsilane **S8** (45.5 mg, 0.168 mmol) was converted to the corresponding cycloadduct using ethyl acrylate as a dienophile. A solution of allylsilane **S8** was added to a reaction mixture at 45 °C. Purification of the crude mixture by flash chromatography on silica gel gave **32** (10.9 mg, 0.050 mmol, 30%) as a colorless oil.

**32**: IR (film) 2936, 1734, 1716, 1541, 1521, 1456, 1437, 1373, 1250, 1177, 1031, 853, 835, 677 $cm^{-1}$; $^{1}$H NMR ($CDCl_3$, 400 MHz) δ 5.31 (br s, 1H), 4.14 (q, *J* = 7.2 Hz, 2H), 2.76 (dd, *J* = 14.8 Hz, 3.6 Hz 1H), 2.62–2.53 (m, 1H), 2.38–2.25 (m, 2H), 2.25–2.17 (m, 2H), 2.12–2.05 (m, 2H), 1.72 (s, 3H), 1.70–1.55 (m, 3H), 1.25 (t, *J* = 7.2 Hz, 3H); $^{13}$C NMR ($CDCl_3$, 100 MHz) δ 176.0, 134.9, 130.8, 126.0, 118.2, 60.4, 40.2, 33.0, 31.5, 28.7, 28.2, 23.3, 19.5, 14.4; HRMS (ESI) *m/z* calcd for $C_{14}H_{20}NaO_2$ $[M+Na]^+$ 243.1356, found 243.1361

***rac*-ethyl 8-methyl-1,2,3,5,6,7-hexahydronaphthalene-2-carboxylate (33)**

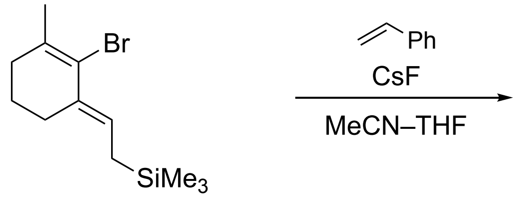


**S8** **33**

Following the general procedure, allylsilane **S8** (54.4 mg, 0.200 mmol) was converted to the corresponding cycloadduct using styrene as a dienophile. Purification of the crude mixture by flash chromatography on silica gel gave **33** (16.6 mg, 0.074 mmol, 37%) as a colorless oil. The [2+2]-cycloadduct was also obtained as an inseparable mixture of isomers (30%).

**33**: IR (film) 2922, 2852, 1717, 1670, 1491, 1458, 995, 839, 831, 760, 698 cm$^{-1}$; $^{1}$H NMR ($CDCl_3$, 400 MHz) δ 7.35–7.30 (m, 2H), 7.29–7.24 (m, 2H), 7.22 (app tt, *J* = 7.2, 1.6 Hz, 1H), 5.45 (br d, *J* = 3.6 Hz, 1H), 2.90–2.81 (m, 1H), 2.77 (dd, *J* = 14.4 Hz, 3.6 Hz 1H), 2.39–2.21 (m, 5H), 2.13 (br s, 2H), 1.78–1.64 (m, 2H), 1.70 (s, 3H); $^{13}$C NMR ($CDCl_3$, 100 MHz) δ 147.2, 134.8, 130.0, 128.5, 127.9, 127.2, 126.2, 120.1, 41.2, 34.0, 33.8, 33.0, 31.6, 23.4, 19.5; HRMS (ESI, APCI) peaks were not detectable.

***rac*-(4a*R*,10a*R*)-5,8,8-trimethyl-3,4,4a,6,7,8,10,10a-octahydrophenanthren-1(2*H*)-one (31)**

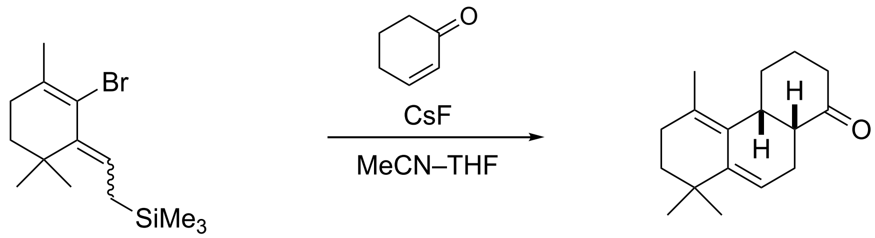


**S5** **31**

Following the general procedure, allylsilane **S5** (128.6 mg, 0.427 mmol) was converted to the corresponding cycloadduct using 2-cyclohexene-1-one as a dienophile. A solution of allylsilane **S5** was added to a reaction mixture via a syringe pump over 2 h. Purification of the crude mixture by flash chromatography on silica gel gave **31** (44.7 mg, 0.183 mmol, 43%) as a colorless oil. A small amount of the dimer (6%) was also formed in an inseparable mixture of isomers.

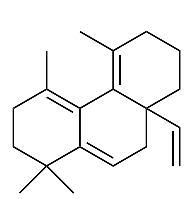

plausible structure
for the dimer (mixture of isomres)

The amount of dimer was increased to 15% when allylsilane **S5** was added to the reaction mixture in one portion.

**31**: IR (film) 2928, 2363, 1709, 1539, 1456, 1420, 759, 548, 500, 488, 482, 453 cm$^{-1}$; $^{1}$H NMR ($CDCl_3$, 600 MHz) δ 5.44 (dd, *J* = 6.0, 2.4 Hz, 1H), 2.91 (dt, *J* = 12.6, 4.2 Hz, 1H), 2.60–2.52 (m, 2H), 2.37 (dd, *J* = 17.4, 6.0 Hz, 1H), 2.27–2.22 (m, 1H), 2.20 (dt, *J* = 18.0, 6.0, Hz, 1H), 2.17–2.09 (m, 1H), 2.06–2.01 (m, 2H), 1.73–1.65 (m, 1H), 1.70 (s, 3H), 1.63 (app qd, *J* = 13.2, 3.6 Hz, 1H), 1.49–1.44 (m, 2H), 1.37 (ddd, *J* = 13.2, 6.0, 4.8 Hz, 1H), 1.04 (s, 3H), 0.95 (s, 3H); $^{13}$C NMR ($CDCl_3$, 150 MHz) δ 215.0, 141.8, 129.1, 127.8, 113.5, 49.5, 38.8, 37.2, 36.4, 33.2, 29.8, 28.5, 27.2, 25.4, 25.2, 24.9, 18.8; HRMS (ESI) *m/z* calcd for $C_{17}H_{24}NaO$ $[M+Na]^+$ 267.1719, found 267.1722

### ***rac*-(4a*R*,10a*R*)-8-methyl-3,4,4a,6,7,8,10,10a-octahydrophenanthren-1(2*H*)-one (28)**

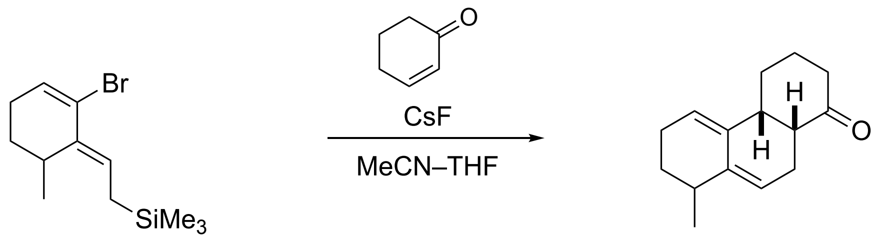


**S20** **28**

Following the general procedure, allylsilane **S20** (55.0 mg, 0.200 mmol) was converted to the corresponding cycloadduct using 2-cyclohexene-1-one as a dienophile. Purification of the crude mixture by flash chromatography on silica gel gave **28** (7.70 mg, 0.0356 mmol, 18%) as a colorless oil.

**28**: IR (film) 3374, 2926, 1707, 1636, 756, 667, 503, cm$^{-1}$; $^{1}$H NMR ($CDCl_3$, 600 MHz) δ 5.51 (dd, *J* = 9.0, 4.2 Hz, 1H (major+minor)), 5.40 (br s, 1H (major+minor)), 2.68-2.60 (m, 2H (major+minor)), 2.53-2.46 (m, 1H (major+minor)), 2.45-2.39 (m, 1H (major+minor)), 2.36-2.22 (m, 2H (major+minor)), 2.22-2.09 (m, 4H (major+minor)), 2.08-2.00 (m, 1H (major+minor)), 1.92-1.82 (m, 1H (major+minor)), 1.77-1.65 (m, 2H (major+minor)), 1.49-1.36 (m, 1H (major+minor)), 1.04 (d, *J* = 6.6, 3H minor), 1.02 (d, *J* = 7.2 3H major); $^{13}$C NMR ($CDCl_3$, 150 MHz) δ 214.2(2C), 138.2, 138.1, 134.7, 134.2, 124.2, 123.9, 117.4, 116.9, 49.8, 49.8, 43.4, 43.2, 38.2, 38.2, 33.9, 33.6, 30.9, 29.9, 28.0, 27.9, 25.4, 25.3, 25.1, 25.0, 24.5, 23.3, 19.4, 19.2; HRMS (ESI) *m/z* calcd for $C_{15}H_{20}NaO$ $[M+Na]^+$ 239.1406, found 239.1413

***rac*-(4b'*R*,8a'*R*)-2',3',4b',5',6',7',8a',9'-octahydro-8'*H*-spiro[cyclohexane-1,1'-phenanthren]-8'-one (29)**

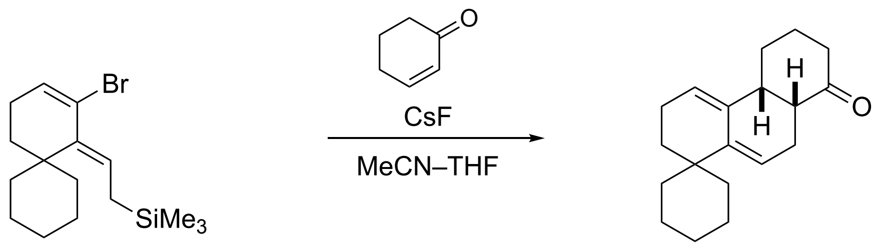

**S16** **29**

Following the general procedure, allylsilane **S16** (65.5 mg, 0.200 mmol) was converted to the corresponding cycloadduct using ethyl acrylate as a dienophile. Purification of the crude mixture by flash chromatography on silica gel gave **29** (25.4 mg, 0.0939 mmol, 47%) as a colorless oil.

**29**: IR (film) 3233, 2915, 2853, 1714, 1701, 1456, 1215, 754, 667, 496 cm$^{-1}$; $^{1}$H NMR (CDCl$_3$, 600 MHz) δ 5.52 (br s, 1H), 5.45 (br s, 1H), 2.67–2.62 (m, 1H), 2.59 (dt, *J* = 11.4 Hz, 4.2 Hz, 1H), 2.51 (ddd, *J* = 15.0 Hz, 12.6 Hz, 6.6 Hz, 1H), 2.42 (ddd, *J* = 17.4 Hz, 10.2 Hz, 1.8 Hz, 1H), 2.26–2.21 (m, 2H), 2.10–2.06 (m, 2H), 2.05–2.00 (m, 1H), 1.85–1.76 (m, 2H), 1.76–1.67 (m, 1H), 1.66–1.61 (m, 1H), 1.60–1.35 (m, 9H), 1.24–1.16 (m, 2H); $^{13}$C NMR (CDCl$_3$, 150 MHz) δ 214.4, 142.5, 134.5, 122.9, 115.0, 49.5, 44.1, 37.8, 35.9, 35.0, 34.3, 29.1, 27.7, 26.8, 25.6, 25.1, 22.3, 22.2, 22.1; HRMS (ESI) *m/z* calcd for $C_{19}H_{26}NaO$ [M+Na]$^+$ 293.1876, found 293.1868

***rac*-methyl 3',5',6',7'-tetrahydro-2'*H*-spiro[cyclohexane-1,1'-naphthalene]-6'-carboxylate (30)**

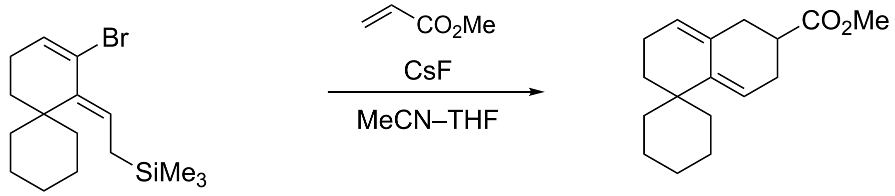

**S16** **30**

Following the general procedure, allylsilane **S16** (65.5 mg, 0.200 mmol) was converted to the corresponding cycloadduct using ethyl acrylate as a dienophile. Purification of the crude mixture by flash chromatography on silica gel gave **30** (29.2 mg, 0.112 mmol, 56%) as a colorless oil.

**30**: IR (film) 2922, 2850, 1736, 1435, 1302, 1267, 1248, 1192, 1167, 1047, 1005, 905, 851, 797, 758 cm$^{-1}$; $^{1}$H NMR (CDCl$_3$, 400 MHz) δ 5.58 (br s, 1H), 5.42 (br s, 1H), 3.68 (s, 3H), 2.65–2.56 (m, 1H), 2.51 (dd, *J* = 14.0 Hz, 3.2 Hz, 1H), 2.40–2.36 (m, 2H), 2.10–2.03 (m, 2H), 1.77 (dt, *J* = 13.2 Hz, 5.2 Hz, 1H), 1.68-1.36 (m,

10H), 1.25–1.15 (m, 2H); $^{13}$C NMR (CDCl$_3$, 100 MHz) δ 176.1, 143.7, 131.6, 122.6, 116.9, 51.8, 40.2, 35.1, 34.5, 34.3, 29.1, 28.9, 26.8, 22.2, 22.1;
HRMS (ESI) *m/z* calcd for $C_{17}H_{24}NaO_2$ [M+Na]$^+$ 283.1669, found 283.1662

***rac*-ethyl 8-methyl-1,2,3,5,6,7-hexahydronaphthalene-2-carboxylate (32)**

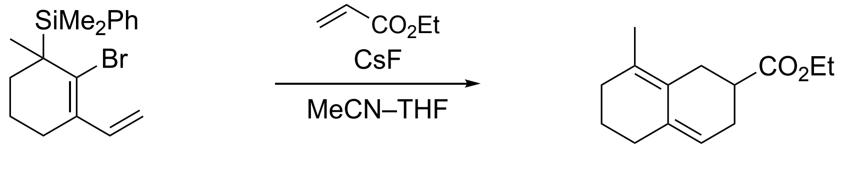


**34** **32**

Following the general procedure, allylsilane **34** (75.6 mg, 0.225 mmol) was converted to the corresponding cycloadduct using ethyl acrylate as a dienophile. Purification of the crude mixture by flash chromatography on silica gel gave **32** (37.8 mg, 0.171 mmol, 76%) as a colorless oil.

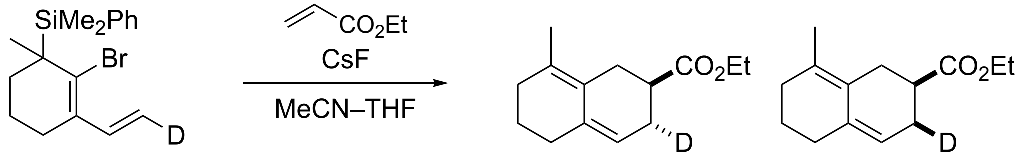


**34-d₁** **32-d₁** (1:2)

A deuterium labeled **34-d$_1$** (65.4 mg, 0.194 mmol) was converted to the corresponding cycloadduct **32-d$_1$** (35.0 mg, 0.158 mmol, 81%). The incorporation ratio of deuterium was as shown.

***rac*-ethyl 4-methyl-1,2,3,5,6,7-hexahydronaphthalene-2-carboxylate (36)**

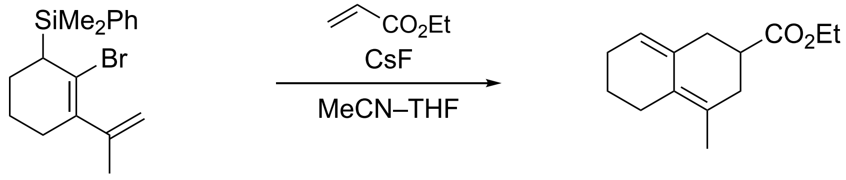


**35** **36**

Following the general procedure, allylsilane **35** (65.0 mg, 0.194 mmol) was converted to the corresponding cycloadduct using methyl acrylate as a dienophile. Purification of the crude mixture by flash chromatography on silica gel gave **36** (19.2 mg, 0.0873 mmol, 45%) as a colorless oil.

**36**: IR (film) 2937, 2926, 1730, 1447, 1375, 1184, 1038, 754, 667 cm$^{-1}$; $^1$H NMR (CDCl$_3$, 600 MHz) δ 5.44 (s, 1H), 3.68 (s, 3H), 2.66-2.60 (m, 1H), 2.48 (dd, *J* = 14.4, 3.0 Hz, 1H),

2.39-2.31 (m, 3H), 2.28-2.18 (m, 2H), 2.08 (app s, 2H), 1.71 (s, 3H), 1.69-1.59 (m, 2H); $^{13}$C NMR ($CDCl_3$, 150 MHz) δ 176.0, 133.0, 127.7, 126.7, 122.5, 51.8, 40.1, 35.0, 34.1, 26.1, 25.7, 23.1, 19.1; HRMS (ESI) *m/z* calcd for $C_{14}H_{20}NaO_2$ $[M+Na]^+$ 243.1356, found 243.1350

### *rac*-ethyl (2*S*,3*R*)-3-cyclopropyl-8-methyl-1,2,3,5,6,7-hexahydronaphthalene-2-carboxylate (38)

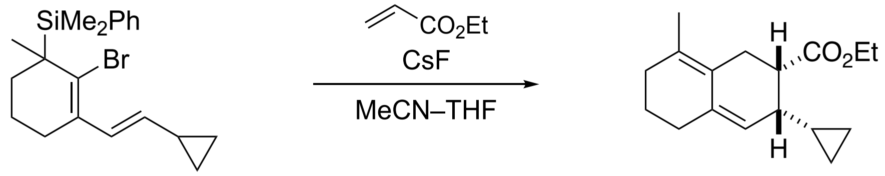


**37** **38**

Following the general procedure, allylsilane **37** (31.0 mg, 0.0829 mmol) was converted to the corresponding cycloadduct using ethyl acrylate as a dienophile. Purification of the crude mixture by flash chromatography on silica gel gave **38** (14.0 mg, 0.0538 mmol, 65%, 8:1 mixture of diastereomers) as a colorless oil.

**38**: IR (film) 3451, 2980, 2936, 1728, 1609, 1445, 1371, 1217, 1180, 1098, 1032, 862, 756, 667 cm$^{-1}$; $^{1}$H NMR ($CDCl_3$, 600 MHz) δ 5.32 (d, J = 5.5 Hz, 1H), 4.22–4.07 (m, 2H), 2.73 (ddd, J = 13.8, 5.4, 4.2 Hz, 1H), 2.61 (dd, J = 15.0, 3.6 Hz, 1H), 2.48 (app t, J = 13.8 Hz, 1H), 2.23–2.19 (m, 2H), 2.12–2.05 (m, 2H), 1.88-1.85 (m, 1H), 1.72 (s, 3H), 1.71–1.65 (m, 1H), 1.65–1.57 (m, 1H), 1.27 (t, J = 7.2 Hz, 3H), 0.70–0.62 (m, 1H), 0.43–0.37 (m, 1H), 0.36–0.30 (m, 1H), 0.14–0.09 (m, 1H), 0.09–0.04 (m, 1H); $^{13}$C NMR ($CDCl_3$, 150 MHz) δ 175.1, 134.6,131.3, 126.1, 121.7, 60.2, 44.0, 41.7, 33.0, 31.6, 24.2, 23.3, 19.6, 14.4, 12.0, 3.8, 2.1; HRMS (ESI) *m/z* calcd for $C_{17}H_{24}NaO_2$ $[M+Na]^+$ 283.1669, found 283.1657;

## References

[74] N. Hatae, I. Suzuki, T. Choshi, S. Hibino, C. Okada, E. Toyota, "Halogen Effect on Tandem [4 + 2] Cycloaddition/Aromatization Sequence of Allenyl 2-Halo-3-vinylcyclohex-2-enyl Ether", *Tetrahedron Lett.* **2014**, *55*, 4146–4148.

[75] M. Chen, G. Dong, "Platinum-Catalyzed α,β-Desaturation of Cyclic Ketones through Direct Metal–Enolate Formation", *Angew. Chem. Int. Ed.* **2021**, *60*, 7956–7961.

[76] C. L. Lankovic, F. G. West, "2 + 2 Trapping of Acyloxy-1,2-cyclohexadienes with Styrenes and Electron-Deficient Olefins", *Org. Lett.* **2022**, *24*, 9497–9501.

[77] F. A. Marques, C. A. Lenz, F. Simonelli, B. H. L. N. S. Maia, A. P. Vellasco, M. N. Eberlin, "Structure Confirmation of a Bioactive Natural Product by ESI-MS/MS and NMR", *J. Nat. Prod.* **2004**, *67*, 1939–1941.

[78] Y.-J. Hu, J.-H. Fan, S. Li, J. Zhao, C.-C. Li, "Total Synthesis of (+)-Frondosin B via a Tandem Cyclization Strategy", *Org. Lett.* **2018**, *20*, 5905–5909.

[79] B. Pedram, A. van Oeveren, D. E. Mais, K. B. Marschke, P. M. Verbost, M. B. Groen, L. Zhi, "A Tissue-Selective Nonsteroidal Progesterone Receptor Modulator: 7,9-Difluoro-5-(3-methylcyclohex-2-enyl)-2,2,4-trimethyl-1,2-dihydrochromeno[3,4-f]quinoline", *J. Med. Chem.* **2008**, *51*, 3696–3699.

[80] M. Inoue, M. W. Carson, A. J. Frontier, S. J. Danishefsky, "Total Synthesis and Determination of the Absolute Configuration of Frondosin B", *J. Am. Chem. Soc.* **2001**, *123*, 1878–1889.

[81] K. Sakamoto, Y. Nagashima, C. Wang, K. Miyamoto, K. Tanaka, M. Uchiyama, "Nucleophilic Germylation of Stable π Bonds via Ge–Ge Bond Cleavage", *J. Am. Chem. Soc.* **2021**, *143*, 5629–5635.

[82] G. J. Kramp, M. Kim, H.-J. Gais, C. Vermeeren, "Stereoselective Synthesis of Optically Active 2-Methyl-4-hexynoic Acid Ethyl Ester Using Benzyl-Substituted Oxazolidine as Chiral Auxiliary", *J. Am. Chem. Soc.* **2005**, *127*, 17910–17920.

## Computational study

### General Method

Density functional theory (DFT) calculations were performed using Gaussian 16 package.[82] Geometries were optimized using ωB97XD[60] with the 6-31G(d) basis set combined with SMD[61] solvation model in MeCN. Tight convergence criteria and the default "ultrafine" pruned (99590) integration grid of Gaussian 16 were applied. Thermochemical corrections were obtained from frequency calculations at the same level of theory. The single point energies were computed at the ωB97XD/6-311+G(d,p) basis set with SMD solvation model in MeCN. Enthalpy and free energies in the solution were computed by adding the gas phase thermochemical corrections to the solution phase single point energy. Computed structures are illustrated using CYLView.[83] The Isosurface of the molecular orbitals was visualized using the Pymol program.[84] Non-covalent interaction (NCI) was calculated by using the NCIPLOT software.[71,72] NCI surfaces were plotted by using the VMD-1.9.4 program.[85]

Quantitative analyses of the activation barriers associated with the cycloaddition were obtained by means of the activation strain model (ASM), which involves decomposing the electronic energy of the transition structure $\Delta E^{\ddagger}$ into the strain $\Delta E^{\ddagger}_{\text{strain}}$ associated with the structural deformation of the reactants from their equilibrium geometry and the interaction $\Delta E^{\ddagger}_{\text{int}}$ between the deformed reactants [Eq. 1]. [47,65–69,71–72] The $\Delta E^{\ddagger}_{\text{strain}}$ is determined by the rigidity of the reactants and by the extent to which they must deform to achieve the geometry of the transition structure. The $\Delta \mathrm{E}^{\ddagger}_{\text{int}}$ is related to the electronic structure of the reactants and how they are mutually oriented over the course of the reaction.

$$\Delta \mathrm{E}^{\ddagger} = \Delta \mathrm{E}^{\ddagger}_{\text{strain}} + \Delta \mathrm{E}^{\ddagger}_{\text{int}} \quad (1)$$

---

83. Gaussian 16, Revision C.01, M. J. Frisch, G. W. Trucks, H. B. Schlegel, G. E. Scuseria, M. A. Robb, J. R. Cheeseman, G. Scalmani, V. Barone, G. A. Petersson, H. Nakatsuji, X. Li, M. Caricato, A. V. Marenich, J. Bloino, B. G. Janesko, R. Gomperts, B. Mennucci, H. P. Hratchian, J. V. Ortiz, A. F. Izmaylov, J. L. Sonnenberg, D. Williams-Young, F. Ding, F. Lipparini, F. Egidi, J. Goings, B. Peng, A. Petrone, T. Henderson, D. Ranasinghe, V. G. Zakrzewski, J. Gao, N. Rega, G. Zheng, W. Liang, M. Hada, M. Ehara, K. Toyota, R. Fukuda, J. Hasegawa, M. Ishida, T. Nakajima, Y. Honda, O. Kitao, H. Nakai, T. Vreven, K. Throssell, J. A. Montgomery Jr., J. E. Peralta, F. Ogliaro, M. J. Bearpark, J. J. Heyd, E. N. Brothers, K. N. Kudin, V. N. Staroverov, T. A. Keith, R. Kobayashi, J. Normand, K. Raghavachari, A. P. Rendell, J. C. Burant, S. S. Iyengar, J. Tomasi, M. Cossi, J. M. Millam, M. Klene, C. Adamo, R. Cammi, J. W. Ochterski, R. L. Martin, K. Morokuma, O. Farkas, J. B. Foresman, D. J. Fox, Gaussian, Inc., Wallingford CT, 2016.

61. J.-D. Chai, M. Head-Gordon “Long-Range Corrected Hybrid Density Functionals with Damped Atom-Atom Dispersion Corrections.” *Phys. Chem. Chem. Phys.* **2008**, *10*, 6615–6620.

62. A. V. Marenich, C. J. Cramer, D. G. Truhlar, "Universal Solvation Model Based on Solute Electron Density and on a Continuum Model of the Solvent Defined by the Bulk Dielectric Constant and Atomic Surface Tensions", *J. Phys. Chem. B* **2009**, *113*, 6378–6396.

84. C. Y. Legault, CYLView, 1.0b; Université de Sherbrooke, Canada, 2009; http://www.cylview.org.

85. The PyMOL Molecular Graphics System, Version 3.0 Schrödinger, LLC.

47. F. Liu, R. S. Paton, S. Kim, Y. Liang, K. N. Houk, "Diels–Alder Reactivities of Strained and Unstrained Cycloalkenes with Normal and Inverse-Electron-Demand Dienes: Activation Barriers and Distortion/Interaction Analysis", *J. Am. Chem. Soc.* **2013**, *135*, 15642–15649.

66. W.-J. van Zeist, F. M. Bickelhaupt, "The Activation Strain Model of Chemical Reactivity", *Org. Biomol. Chem.* **2010**, *8*, 3118–3127.

67. I. Fernández, F. M. Bickelhaupt, "The Activation Strain Model and Molecular Orbital Theory: Understanding and Designing Chemical Reactions", *Chem. Soc. Rev.* **2014**, *43*, 4953–4967.

68. I. Fernández, F. M. Bickelhaupt, "Deeper Insight into the Diels–Alder Reaction through the Activation Strain Model", *Chem. Asian J.* **2016**, *11*, 3297–3304.

69. F. M. Bickelhaupt, K. N. Houk, "Analyzing Reaction Rates with the Distortion/Interaction–Activation Strain Model", *Angew. Chem. Int. Ed.* **2017**, *56*, 10070–10086.

70. P. Vermeeren, S. C. C. van der Lubbe, C. Fonseca Guerra, F. M. Bickelhaupt, T. A. Hamlin, "Understanding Chemical Reactivity Using the Activation Strain Model", *Nat. Protoc.* **2020**, *15*, 649–667.

72. E. R. Johnson, S. Keinan, P. Mori-Sánchez, J. Contreras-García, A. J. Cohen, W. Yang, "Revealing Noncovalent Interactions", *J. Am. Chem. Soc.* **2010**, *132*, 6498–6506.

73. J. Contreras-García, E. R. Johnson, S. Keinan, R. Chaudret, J.-P. Piquemal, D. N. Beratan, W. Yang, "NCIPLOT: A Program for Plotting Noncovalent Interaction Regions", *J. Chem. Theory Comput.* **2011**, *7*, 625–632.

86. K. Yamazaki, P. Gabriel, F. Di Carmine, J. Pedroni, M. Farizyan, T. A. Hamlin, D. J. “Dixon General Pyrrolidine Synthesis via Iridium-Catalyzed Reductive Azomethine Ylide Generation from Tertiary Amides and Lactams.” *ACS Catal.* **2021**, *11*, 7489–7497.

30. M. Ramirez, D. Svatunek, F. Liu, N. K. Garg, K. N. Houk, “Origins of Endo Selectivity in Diels-Alder Reactions of Cyclic Allene Dienophiles.” *Angew. Chem. Int. Ed Engl.* **2021**, *60*, 14989–14997.

**Geometries of transition states**

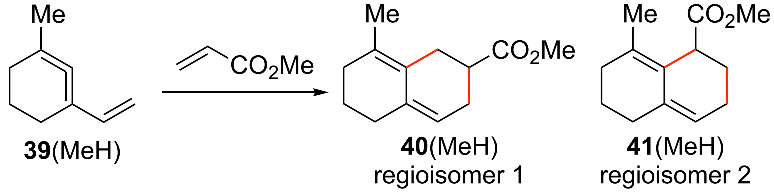


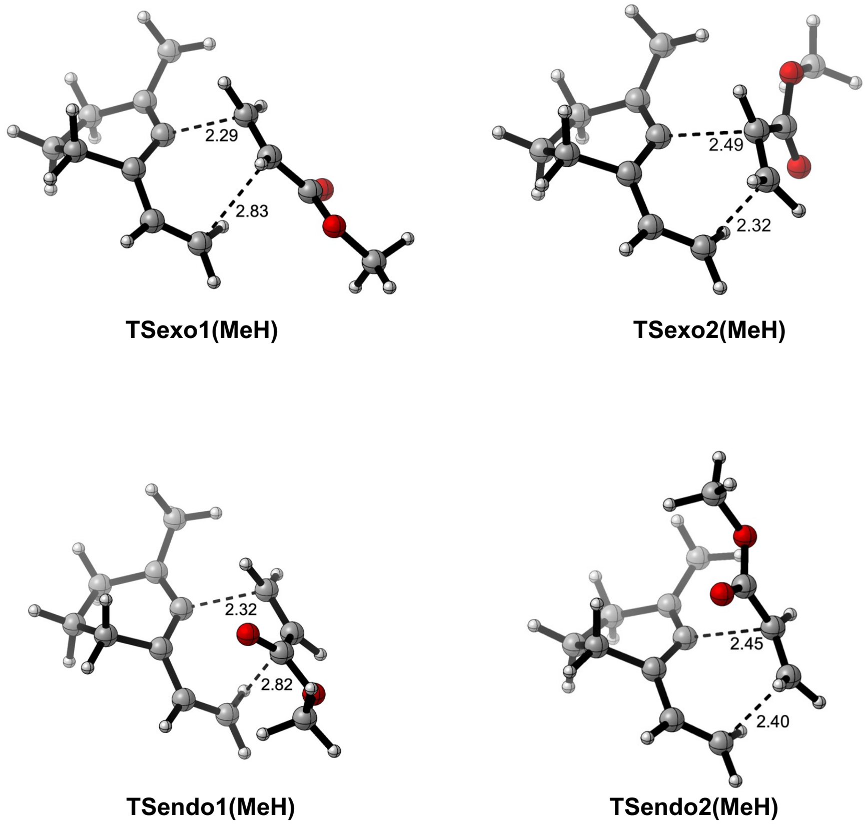


**Figure S1.**Transition states geometries for concerted cycloadditions of allene **39** and methyl acrylate.

Geometries were optimized using ωB97XD with the 6-31G(d) basis set combined with SMD solvation model in MeCN.

| (kcal/mol) | $\Delta G^{\ddagger}$ | $\Delta E^{\ddagger}$ | $\Delta E^{\ddagger}_{strain}$ | $\Delta E^{\ddagger}_{int}$ | $\Delta E^{\ddagger}_{strain,diene}$ | $\Delta E^{\ddagger}_{straine,dienophile}$ |
|---|---|---|---|---|---|---|
| **TSexo1** | 15.9 | 1.8 | 7.7 | -5.9 | 4.2 | 3.5 |
| **TSendo1** | 14.3 | 0.3 | 6.6 | -6.3 | 3.6 | 3.1 |
| **TSexo2** | 20.5 | 5.7 | 13.0 | -7.3 | 7.8 | 5.2 |
| **TSendo2** | 18.6 | 3.7 | 11.8 | -8.1 | 8.0 | 3.7 |

The single point energies for the transition state structures and optimized structure of the reactants were computed at the ωB97XD/6-311+G(d,p) basis set with SMD solvation model in MeCN.

The trend in Gibbs free energy barriers ($\Delta G^{\ddagger}$ in kcal/mol) is endo-1 (14.3) < exo-1 (15.9) << endo-2 (18.6) < exo-2 (20.5). Since the experiment produces the *endo*-product of regioisomer-1 as the major product and the *exo*-product of regioisomer-1 as a minor product without affording the regioisomer-2, this calculated trend could be in good agreement.

The activation strain analysis (ASA) at transition structures revealed that the electronic activation barriers ($\Delta E^{\ddagger}$) follow the same trend as $\Delta G^{\ddagger}$. The differences in reactivity can be traced back to the differences in the strain energy ($\Delta E^{\ddagger}_{\text{strain}}$). Bickelhaupt, Fernández, and Hamlin previously proposed that the asynchronicity of the Diels-Alder reaction is linked to the strain of the reactants. A higher degree of asynchronicity leads to a lower destabilizing strain energy than the synchronous TS. The difference in distance between the two forming carbon-carbon bonds in the calculated transition states is 0.54 Å and 0.50 Å for TSexo1 and TSendo1, and 0.17 Å and 0.05 Å for TSexo2 and TSendo2. Accordingly, TSexo1 and TSendo1 are found to be highly asynchronous compared to TSexo2 and TSendo2. These results suggest that a similar relationship between asynchronicity and strain energy is observed in our vinyl cyclic allene Diels-Alder reaction. In addition, Dixon and Hamlin reported a linear relationship between $\Delta E^{\ddagger}_{\text{strain}}$ and the degree of pyramidization at the reacting carbon atoms (calculated as 720° – $[\text{SoA}]_n$, SoA = sum of angles around a carbon atom in degrees), which arises from changes in hybridization during bond formation.[85] This suggests that as the Diels-Alder reaction becomes more asynchronous, the degree of pyramidization decreases, and when $\Delta\Delta E^{\ddagger}_{\text{int}}$ is small, $\Delta G^{\ddagger}$ decreases.

Consequently, the SoA analysis was performed for the vinyl cyclic allene Diels-Alder reaction to elucidate the relationship between $\Delta E^{\ddagger}_{\text{strain}}$ and structural changes during the reaction. The normalized SoA of 1) pyramidization at two reacting carbon atoms of a dienophile and terminal alkene carbon atom of diene, and 2) planarization at the reacting central *sp*-hybridized allene carbon were calculated. Due to the *sp*-hybridized character of the allene carbon, 180° was used as the maximum angle for calculating planarization. $\text{SoA}_1$ (the planarization of the central carbon of the allene) and $\text{SoA}_2$ (the pyramidalization of the terminal alkene carbon atom of the diene) were considered together to represent the distortion of the diene. Similarly, $\text{SoA}_3$ and $\text{SoA}_4$ (the pyramidalization of the two carbons of the dienophile) were considered together to represent the distortion of the dienophile. The relationship between these structural changes was plotted against the strain energy (Figure S2.). Modest linear relationships were observed for both diene ($[540°–\text{SoA}_1–\text{SoA}_2]/\Delta E^{\ddagger}_{\text{strain,diene}}$) and dienophile ($[720°–\text{SoA}_3–\text{SoA}_4]/\Delta E^{\ddagger}_{\text{strain,dienophile}}$), which is consistent with a previous report.[85] Although the interaction energy is also important for this reaction, especially for the difference between *endo*- and *exo*-TS, the SoA analysis suggests that the reaction producing regioisomer-1 benefits from a less destabilizing strain energy by the asynchronicity of the TS.

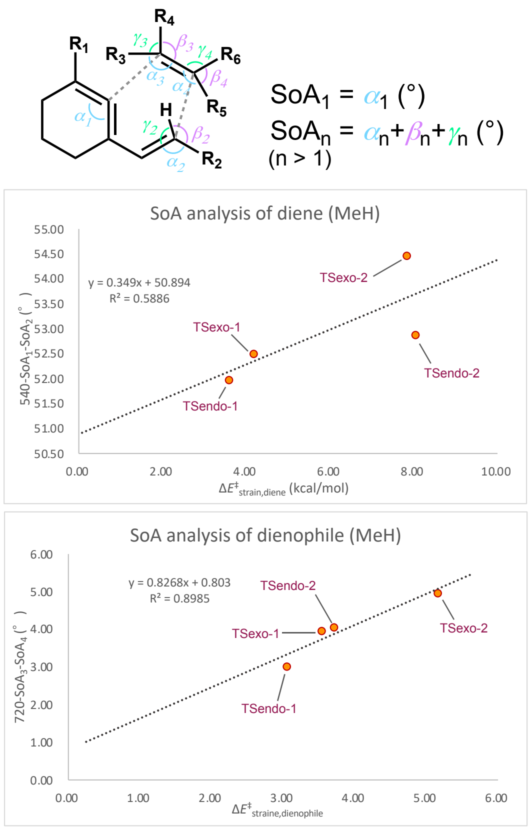


**Figure S2.** Correlation between the Sum of Angles (SoA in degrees) and the strain energy of the diene and dienophile.

In addition, we calculated the molecular orbital (MO) coefficients of the HOMO to rationalize the observed regioselectivity. The calculated isosurface and MO coefficients of the HOMO of vinyl cyclic allene **39** are shown in Figure S3.[30] The coordinates were extracted from the transition state structure **TSendo1**, in which the forming bond between the central carbon of the allene and the β-carbon of the acrylate is aligned along the y-axis. The MO structures and coefficients were obtained at the HF/6-31G(d,p) level of theory. Similar calculations performed using structures from **TSexo1**, **TSendo2**, and **TSexo2**, yielded nearly identical HOMO coefficients.

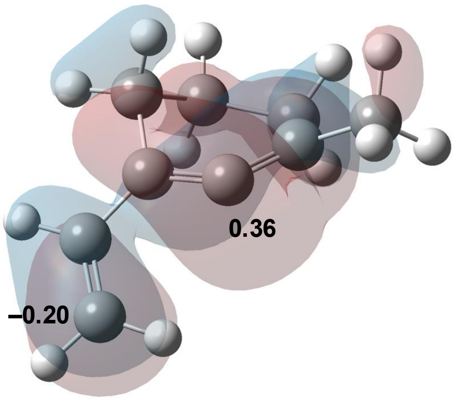


**Figure S3.** Calculated HOMO isosurface and MO coefficients of vinyl cyclic allene extracted from the transition state (**TSendo1**).

These results demonstrate that the HOMO of **39** has a larger coefficient at the central carbon of the allene compared to the terminal carbon. Consequently, Diels-Alder reactions with electron-deficient dienophiles such as acrylates are expected to occur with preferential bonding at the central carbon of the allene. This trend is consistent with the experimentally observed regioselectivity.

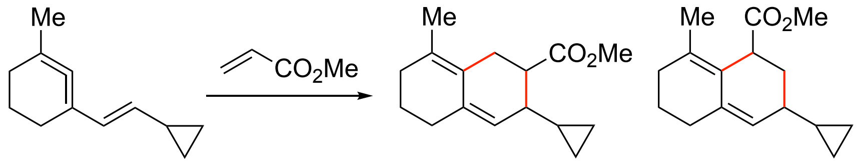


**42**(MeCP) **43**(MeCP) **S27**(MeCP)

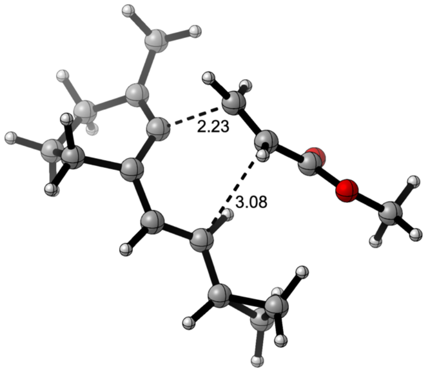


**TSexo1(MeCP)**

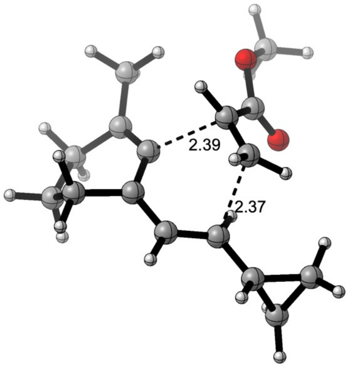


**TSexo2(MeCP)**

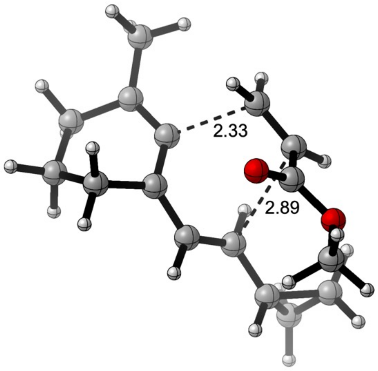


**TSendo1(MeCP)**

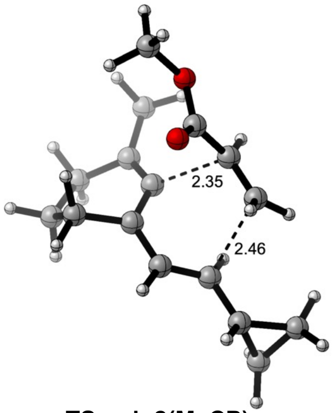


**TSendo2(MeCP)**

**Figure S4.** Transition states geometries for concerted cycloadditions of allene **42** and methyl acrylate.

Geometries were optimized using ωB97XD with the 6-31G(d) basis set combined with SMD solvation model in MeCN.

| (kcal/mol) | $\Delta G^{\ddagger}$ | $\Delta E^{\ddagger}$ | $\Delta E^{\ddagger}_{strain}$ | $\Delta E^{\ddagger}_{int}$ | $\Delta E^{\ddagger}_{strain,diene}$ | $\Delta E^{\ddagger}_{straine,dienophile}$ |
|---|---|---|---|---|---|---|
| **TSexo1** | 16.2 | 1.6 | 7.5 | -5.9 | 2.7 | 4.8 |
| **TSendo1** | 13.3 | -1.1 | 5.7 | -6.8 | 2.6 | 3.1 |
| **TSexo2** | 21.3 | 6.2 | 13.7 | -7.5 | 7.8 | 5.9 |
| **TSendo2** | 19.1 | 4.0 | 11.9 | -7.9 | 7.7 | 4.2 |

The single point energies for the transition state structures and optimized structure of the reactants were computed at the ωB97XD/6-311+G(d,p) basis set with SMD solvation model in MeCN.

**Energies and Cartesian Coordinates for Optimized Structures**

Cartesian coordinates (in Å), energies (in kcal mol$^{-1}$), and number of imaginary frequencies of all stationary points, computed at SMD(MeCN)/ωB97XD/6- 311+G(d,p)// SMD(MeCN)/ωB97XD/6-31G(d).

**TSexo1(MeH)**

***E*** = -656.51425

***G*** = -656.275075

$\boldsymbol{N}_{\mathbf{imag}}$ = 1, 275*i* cm$^{-1}$

| | | | |
|---|---|---|---|
| C | -3.48910 | 0.44813 | -0.87330 |
| C | -2.77241 | -1.46392 | 0.75149 |
| C | -1.37621 | -1.06758 | 0.32462 |
| C | -1.28683 | 0.27008 | 0.09613 |
| C | -2.26474 | 1.10985 | -0.24055 |
| C | -0.34740 | -2.02628 | 0.05094 |
| C | 0.79001 | -1.72629 | -0.61538 |
| C | 1.65089 | 0.35596 | 1.09904 |
| C | 2.70601 | 0.47491 | 0.09023 |
| C | -3.67702 | -1.03108 | -0.43486 |
| O | 2.67215 | 1.18909 | -0.89869 |
| H | -0.48191 | -3.02813 | 0.45692 |
| H | 0.90725 | -0.77789 | -1.12690 |
| H | 1.57174 | -2.46701 | -0.75934 |
| H | 1.80586 | -0.35475 | 1.90313 |
| H | -4.72621 | -1.19495 | -0.16664 |
| H | -3.45625 | -1.69581 | -1.27696 |
| C | 0.56958 | 1.18324 | 1.06769 |
| H | -0.06676 | 1.29116 | 1.93884 |

| | | | |
|---|---|---|---|
| H | -3.45312 | 0.51571 | -1.96710 |
| H | -4.35648 | 1.04461 | -0.56316 |
| H | -3.07958 | -0.95139 | 1.66895 |
| H | -2.86249 | -2.54304 | 0.91643 |
| C | -2.34716 | 2.58122 | 0.03176 |
| H | -2.58854 | 3.13192 | -0.88650 |
| H | -3.15886 | 2.78324 | 0.74272 |
| H | -1.42213 | 2.98667 | 0.44660 |
| H | 0.55156 | 1.98894 | 0.34066 |
| O | 3.76317 | -0.30717 | 0.38399 |
| C | 4.84014 | -0.26958 | -0.55106 |
| H | 5.59554 | -0.95212 | -0.16022 |
| H | 4.50937 | -0.60385 | -1.53852 |
| H | 5.25379 | 0.73940 | -0.63181 |

**TSendo1(MeH)**

***E*** = -656.5166392

***G*** = -656.2776942

$N_{imag}$ = 1, 241*i* cm$^{-1}$

| | | | |
|---|---|---|---|
| C | 3.59867 | -0.28391 | 0.08836 |
| C | 1.61786 | -1.65301 | -0.92254 |
| C | 0.79585 | -0.88511 | 0.08930 |
| C | 1.28504 | 0.36907 | 0.27219 |
| C | 2.52675 | 0.80653 | 0.07540 |

| | | | |
|---|---|---|---|
| C | -0.24860 | -1.49113 | 0.86226 |
| C | -0.82411 | -0.90299 | 1.93354 |
| C | -0.42388 | 1.93123 | 0.13387 |
| C | 3.05125 | -1.67972 | -0.32364 |
| H | 4.37432 | 0.03843 | -0.61797 |
| H | 4.08986 | -0.34558 | 1.06674 |
| H | -0.63188 | -2.44769 | 0.50951 |
| H | -0.42219 | 0.00738 | 2.36342 |
| H | -1.65171 | -1.37371 | 2.45680 |
| H | 0.10418 | 2.65139 | 0.75031 |
| H | 3.73669 | -2.13377 | -1.04731 |
| H | 3.03014 | -2.33909 | 0.55084 |
| C | -1.61295 | 1.42434 | 0.55389 |
| H | -2.02182 | 1.64639 | 1.53316 |
| C | -2.38427 | 0.55314 | -0.33110 |
| O | -2.01435 | 0.15580 | -1.42349 |
| H | 1.62336 | -1.15873 | -1.89946 |
| H | 1.25162 | -2.67618 | -1.06058 |
| C | 2.96516 | 2.19789 | -0.26900 |
| H | 3.75779 | 2.53464 | 0.41161 |
| H | 3.39006 | 2.21643 | -1.28101 |
| H | 2.14507 | 2.91791 | -0.22889 |
| H | -0.15717 | 1.89022 | -0.91667 |
| O | -3.57966 | 0.22209 | 0.19770 |
| C | -4.36826 | -0.67634 | -0.57883 |

| | | | |
|---|---|---|---|
| H | -3.84933 | -1.62997 | -0.71463 |
| H | -5.28770 | -0.83071 | -0.01284 |
| H | -4.60028 | -0.25042 | -1.55901 |

**TSexo2(MeH)**

***E*** = -656.5079599

***G*** = -656.2677149

$N_{imag}$ = 1, 406*i* $cm^{-1}$

| | | | |
|---|---|---|---|
| C | 2.17939 | -1.92686 | -0.63209 |
| C | 3.03265 | 0.16939 | 0.66023 |
| C | 1.73371 | 0.78542 | 0.18200 |
| C | 0.73862 | -0.14767 | 0.12025 |
| C | 0.85849 | -1.46622 | -0.00918 |
| C | 1.61580 | 2.13004 | -0.23899 |
| C | 0.49250 | 2.61368 | -0.85362 |
| C | -1.10298 | 2.37896 | 0.80781 |
| C | 3.35219 | -0.93692 | -0.37908 |
| H | 2.40421 | 2.82253 | 0.05365 |
| H | -0.19834 | 1.94716 | -1.35896 |
| H | 0.41976 | 3.66755 | -1.11005 |
| H | -0.60660 | 2.95105 | 1.58425 |
| H | 4.23294 | -1.50073 | -0.05317 |
| H | 3.62260 | -0.43946 | -1.31693 |
| C | -1.29064 | 1.03075 | 0.95429 |

| | | | |
|---|---|---|---|
| H | -1.03763 | 0.51404 | 1.87157 |
| H | 2.06552 | -2.10653 | -1.70780 |
| H | 2.41028 | -2.90230 | -0.18609 |
| H | 2.93369 | -0.26667 | 1.66000 |
| H | 3.84594 | 0.90322 | 0.68808 |
| C | -0.07139 | -2.53049 | 0.49146 |
| H | -0.43745 | -3.15126 | -0.33754 |
| H | 0.46739 | -3.20207 | 1.17193 |
| H | -0.93194 | -2.11194 | 1.01412 |
| C | -2.18550 | 0.34965 | 0.00277 |
| O | -2.44376 | 0.74257 | -1.12090 |
| O | -2.72087 | -0.76688 | 0.52237 |
| C | -3.52029 | -1.54947 | -0.36555 |
| H | -4.41029 | -0.99598 | -0.67635 |
| H | -2.94543 | -1.83883 | -1.24927 |
| H | -3.80927 | -2.43524 | 0.20047 |
| H | -1.70377 | 2.93833 | 0.09858 |

**TSendo2(MeH)**

***E*** = -656.5112834

***G*** = -656.2708194

$N_{imag}$ = 1, 360*i* cm$^{-1}$

| | | | |
|---|---|---|---|
| C | 2.62784 | -1.30488 | -0.45578 |
| C | 1.78631 | 0.14148 | 1.54907 |

| | | | |
|---|---|---|---|
| C | 1.06034 | 0.91717 | 0.47246 |
| C | 0.67398 | 0.09821 | -0.54641 |
| C | 1.19519 | -1.05849 | -0.93714 |
| C | 0.89704 | 2.32398 | 0.46322 |
| C | 0.43296 | 2.99437 | -0.63322 |
| C | -1.64973 | 0.74470 | -0.96105 |
| C | 2.99993 | -0.49466 | 0.81760 |
| H | 2.69064 | -2.38077 | -0.24525 |
| H | 3.35720 | -1.11185 | -1.25179 |
| H | 1.03831 | 2.85954 | 1.40113 |
| H | 0.49046 | 2.54421 | -1.61754 |
| H | 0.25726 | 4.06615 | -0.59842 |
| H | -1.58869 | 0.22164 | -1.90874 |
| H | 3.54393 | -1.14643 | 1.51001 |
| H | 3.68190 | 0.31923 | 0.54718 |
| C | -1.78304 | 2.10217 | -0.90766 |
| H | -1.83975 | 2.68905 | -1.81788 |
| H | 1.15208 | -0.63733 | 1.98593 |
| H | 2.13529 | 0.79088 | 2.35974 |
| C | 0.50529 | -2.17660 | -1.65892 |
| H | 1.11254 | -2.52164 | -2.50567 |
| H | 0.37466 | -3.03598 | -0.98808 |
| H | -0.47871 | -1.88024 | -2.02581 |
| C | -1.95640 | -0.05877 | 0.23745 |
| O | -2.12482 | -1.35727 | -0.06923 |

| | | | |
|---|---|---|---|
| O | -2.07405 | 0.37393 | 1.36853 |
| C | -2.31501 | -2.23951 | 1.03456 |
| H | -1.45183 | -2.20883 | 1.70586 |
| H | -3.21801 | -1.98096 | 1.59412 |
| H | -2.41533 | -3.23596 | 0.60315 |
| H | -2.09921 | 2.58099 | 0.01253 |

**TSexo1(MeCP)**

***E*** = -773.2182124

***G*** = -772.9200034

$N_{imag}$ = 1, 288*i* $cm^{-1}$

| | | | |
|---|---|---|---|
| C | -3.91270 | -0.04183 | 0.98555 |
| C | -3.08020 | 1.32867 | -1.06411 |
| C | -1.72313 | 0.84402 | -0.60748 |
| C | -1.78033 | -0.42586 | -0.10955 |
| C | -2.83718 | -0.99867 | 0.47197 |
| C | -0.60625 | 1.73417 | -0.55214 |
| C | 0.57486 | 1.47052 | 0.06321 |
| C | 1.00641 | -1.30952 | -1.18008 |
| C | 2.03078 | -1.49315 | -0.15952 |
| C | -3.94210 | 1.31161 | 0.22704 |
| O | 1.83551 | -1.81603 | 1.00256 |
| H | -0.72773 | 2.69886 | -1.04505 |
| H | 0.70265 | 0.53795 | 0.60631 |

| | | | |
|---|---|---|---|
| H | 1.29649 | -0.85604 | -2.12162 |
| H | -4.97743 | 1.55964 | -0.02973 |
| H | -3.57531 | 2.11129 | 0.87953 |
| C | -0.24783 | -1.80543 | -0.96556 |
| H | -0.93461 | -1.93389 | -1.79478 |
| H | -3.79802 | 0.13110 | 2.06246 |
| H | -4.87335 | -0.55884 | 0.86569 |
| H | -3.51442 | 0.66485 | -1.81825 |
| H | -3.04426 | 2.34192 | -1.47767 |
| C | -3.14285 | -2.46201 | 0.59718 |
| H | -3.39563 | -2.71621 | 1.63464 |
| H | -4.02321 | -2.71003 | -0.01023 |
| H | -2.32067 | -3.10349 | 0.27501 |
| H | -0.39871 | -2.44490 | -0.10299 |
| O | 3.27451 | -1.28164 | -0.64718 |
| C | 4.34108 | -1.42930 | 0.28955 |
| H | 5.25219 | -1.19238 | -0.26082 |
| H | 4.22253 | -0.74026 | 1.13053 |
| H | 4.38784 | -2.45454 | 0.66685 |
| C | 1.66837 | 2.45305 | 0.13048 |
| C | 2.68724 | 2.35391 | 1.24934 |
| C | 3.10393 | 2.03900 | -0.14985 |
| H | 1.40406 | 3.45532 | -0.19798 |
| H | 2.57129 | 1.52619 | 1.94420 |
| H | 3.02855 | 3.28268 | 1.69657 |

| | | | |
|---|---|---|---|
| H | 3.71742 | 2.76264 | -0.67852 |
| H | 3.27959 | 1.00451 | -0.42339 |

**TSendo1(MeCP)**

***E*** = -773.2226275

***G*** = -772.9246635

$N_{imag}$ = 1, 227*i* cm$^{-1}$

| | | | |
|---|---|---|---|
| C | 3.92552 | 0.82743 | 0.09752 |
| C | 2.16331 | 0.16234 | 1.90293 |
| C | 1.18806 | 0.30324 | 0.75514 |
| C | 1.74801 | -0.09300 | -0.42106 |
| C | 3.03889 | -0.08668 | -0.74905 |
| C | -0.07994 | 0.94500 | 0.91208 |
| C | -0.87863 | 1.31558 | -0.11999 |
| C | 0.34403 | -1.61498 | -1.49379 |
| C | 3.34918 | 1.08878 | 1.51661 |
| H | 4.90009 | 0.32820 | 0.17567 |
| H | 4.11642 | 1.77929 | -0.41279 |
| H | -0.43659 | 1.09550 | 1.93104 |
| H | -0.50827 | 1.22403 | -1.13857 |
| H | 0.77691 | -1.40450 | -2.46624 |
| H | 4.14964 | 0.97764 | 2.25604 |
| H | 2.99420 | 2.12290 | 1.58613 |
| C | -0.96588 | -1.34006 | -1.26096 |

| | | | |
|---|---|---|---|
| H | -1.57888 | -0.81208 | -1.98271 |
| C | -1.57955 | -1.72129 | 0.00653 |
| O | -1.00163 | -2.24620 | 0.94505 |
| H | 2.50914 | -0.87008 | 2.01657 |
| H | 1.73223 | 0.48520 | 2.85683 |
| C | 3.72437 | -0.95179 | -1.76480 |
| H | 4.30785 | -0.34105 | -2.46625 |
| H | 4.43546 | -1.62464 | -1.26783 |
| H | 3.02165 | -1.55979 | -2.33898 |
| H | 0.87400 | -2.31904 | -0.86191 |
| O | -2.89538 | -1.42190 | 0.03783 |
| C | -3.54614 | -1.62928 | 1.28674 |
| H | -3.07932 | -1.02615 | 2.07158 |
| H | -4.57950 | -1.31218 | 1.14052 |
| H | -3.51698 | -2.68296 | 1.57897 |
| C | -2.19975 | 1.93439 | 0.05030 |
| C | -2.62432 | 3.05033 | -0.89250 |
| C | -3.27156 | 1.70773 | -0.99486 |
| H | -2.54425 | 2.01954 | 1.07805 |
| H | -1.91965 | 3.33956 | -1.66742 |
| H | -3.19054 | 3.87051 | -0.46106 |
| H | -4.29012 | 1.59214 | -0.63690 |
| H | -3.01108 | 1.07629 | -1.83889 |

**TSexo2(MeCP)**

***E*** = -773.2109012

***G*** = -772.9118722

$N_{imag}$ = 1, 403*i* cm$^{-1}$

| | | | |
|---|---|---|---|
| C | -2.63154 | -1.98209 | -1.02295 |
| C | -1.05558 | -2.98024 | 0.78846 |
| C | -0.22787 | -1.74282 | 0.51463 |
| C | -1.01957 | -0.66453 | 0.21401 |
| C | -2.24827 | -0.68787 | -0.30033 |
| C | 1.17951 | -1.75045 | 0.47716 |
| C | 1.92357 | -0.68778 | 0.01892 |
| C | 1.25973 | 0.90539 | 1.64058 |
| C | -1.84219 | -3.22408 | -0.52488 |
| H | 1.69777 | -2.60275 | 0.91756 |
| H | 1.44307 | 0.06012 | -0.60795 |
| H | 1.50136 | 0.35261 | 2.54243 |
| H | -2.53672 | -4.06011 | -0.38756 |
| H | -1.12006 | -3.53343 | -1.28867 |
| C | -0.04301 | 1.20484 | 1.33536 |
| H | -0.84700 | 1.04471 | 2.04304 |
| H | -2.51532 | -1.87413 | -2.10840 |
| H | -3.70522 | -2.12909 | -0.84972 |
| H | -1.74946 | -2.83134 | 1.62296 |
| H | -0.42892 | -3.84945 | 1.01761 |
| C | -3.32628 | 0.34535 | -0.15313 |

| | | | |
|---|---|---|---|
| H | -3.63624 | 0.72963 | -1.13465 |
| H | -4.21686 | -0.10885 | 0.30001 |
| H | -3.00988 | 1.18916 | 0.46057 |
| C | -0.27789 | 2.16687 | 0.24113 |
| O | 0.47651 | 2.36168 | -0.69519 |
| O | -1.42825 | 2.84010 | 0.39676 |
| C | -1.79031 | 3.71833 | -0.67017 |
| H | -1.06045 | 4.52552 | -0.77296 |
| H | -1.86185 | 3.17016 | -1.61331 |
| H | -2.76411 | 4.12623 | -0.39861 |
| H | 2.06973 | 1.44768 | 1.16829 |
| C | 3.40088 | -0.70734 | -0.01842 |
| C | 4.09162 | -0.54803 | -1.36345 |
| C | 4.16623 | 0.55208 | -0.35124 |
| H | 3.87637 | -1.39530 | 0.67646 |
| H | 3.45769 | -0.3971 | -2.23289 |
| H | 4.97561 | -1.15318 | -1.54175 |
| H | 5.10727 | 0.71990 | 0.16404 |
| H | 3.58280 | 1.44901 | -0.54301 |

**TSendo2(MeCP)**

***E*** = -773.214462

***G*** = -772.915389

$\boldsymbol{N_{imag}}$ = 1, 346*i* cm$^{-1}$

| | | | |
|---|---|---|---|
| C | -1.77622 | 2.73847 | 0.63746 |
| C | -0.85004 | 1.80459 | -1.61437 |
| C | 0.05059 | 0.98981 | -0.71461 |
| C | -0.58650 | 0.64291 | 0.44556 |
| C | -1.57316 | 1.28282 | 1.06531 |
| C | 1.40827 | 0.72159 | -0.98649 |
| C | 2.26976 | 0.20258 | -0.05120 |
| C | -0.14483 | -1.65333 | 0.72181 |
| C | -1.20439 | 3.05901 | -0.77090 |
| H | -2.86181 | 2.90501 | 0.64505 |
| H | -1.36262 | 3.43383 | 1.37844 |
| H | 1.76193 | 0.85627 | -2.00902 |
| H | 1.99702 | 0.26438 | 0.99919 |
| H | -0.45080 | -1.58264 | 1.75967 |
| H | -1.92387 | 3.67792 | -1.31835 |
| H | -0.28931 | 3.65315 | -0.66865 |
| C | 1.15015 | -1.94115 | 0.39249 |
| H | 1.89760 | -2.07799 | 1.16493 |
| H | -1.75912 | 1.25536 | -1.88234 |
| H | -0.34724 | 2.10491 | -2.54055 |
| C | -2.57546 | 0.70927 | 2.02276 |
| H | -2.67547 | 1.35218 | 2.90694 |
| H | -3.56597 | 0.65876 | 1.55125 |
| H | -2.30554 | -0.29691 | 2.34748 |
| C | -1.21086 | -1.88285 | -0.27517 |

| | | | |
|---|---|---|---|
| O | -2.42077 | -1.95576 | 0.30634 |
| O | -1.04387 | -2.01796 | -1.47240 |
| C | -3.53269 | -2.07010 | -0.57899 |
| H | -3.57955 | -1.20941 | -1.25240 |
| H | -3.47253 | -2.98854 | -1.16914 |
| H | -4.41716 | -2.09270 | 0.05835 |
| H | 1.39957 | -2.27725 | -0.60789 |
| C | 3.68581 | -0.09277 | -0.34748 |
| C | 4.76348 | 0.57066 | 0.49601 |
| C | 4.52168 | -0.90101 | 0.61797 |
| H | 3.92128 | -0.21022 | -1.40251 |
| H | 4.43318 | 1.21007 | 1.31004 |
| H | 5.65662 | 0.91145 | -0.01937 |
| H | 5.25380 | -1.58281 | 0.19553 |
| H | 4.03372 | -1.25808 | 1.52089 |

**Vinyl cyclic allene (MeH) 39**

***E*** = -350.0551978

***G*** = -349.9050558

$N_{imag}$ = 0

| | | | |
|---|---|---|---|
| C | 1.65656 | 0.67377 | -0.71980 |
| C | -0.33062 | 1.39179 | 0.83682 |
| C | -0.94984 | 0.08726 | 0.36104 |
| C | 0.00036 | -0.82732 | 0.13458 |

| | | | |
|---|---|---|---|
| C | 1.29652 | -0.68254 | -0.10690 |
| C | -2.33673 | 0.00510 | -0.08532 |
| C | -2.98532 | -1.14026 | -0.33042 |
| C | 0.66161 | 1.79425 | -0.29387 |
| H | 2.66814 | 0.91430 | -0.36875 |
| H | 1.71871 | 0.62215 | -1.81281 |
| H | -2.85878 | 0.95415 | -0.20502 |
| H | -2.51155 | -2.10512 | -0.16085 |
| H | 1.23030 | 2.67371 | 0.02712 |
| H | 0.06441 | 2.10207 | -1.15917 |
| H | 0.20212 | 1.26257 | 1.78351 |
| H | -1.07661 | 2.18196 | 0.97490 |
| C | 2.40058 | -1.61866 | 0.28231 |
| H | 2.97864 | -1.93179 | -0.59706 |
| H | 3.10250 | -1.11569 | 0.95957 |
| H | 2.01405 | -2.51390 | 0.77738 |
| H | -4.01068 | -1.14476 | -0.68931 |

**Vinyl cyclic allene (MeCP) 42**

***E*** = -466.7589184

***G*** = -466.5504854

$N_{imag}$ = 0

| | | | |
|---|---|---|---|
| C | 3.00488 | -0.10884 | 0.88952 |
| C | 1.59504 | -1.60846 | -0.73261 |
| C | 0.53330 | -0.54533 | -0.51520 |
| C | 1.09243 | 0.66320 | -0.34835 |

| | | | |
|---|---|---|---|
| C | 2.31541 | 0.97942 | 0.06229 |
| C | -0.85028 | -0.89352 | -0.22947 |
| C | -1.85114 | 0.00193 | -0.16530 |
| C | 2.48378 | -1.53389 | 0.54380 |
| H | 4.07757 | -0.03695 | 0.66723 |
| H | 2.90664 | 0.07594 | 1.96578 |
| H | -1.06693 | -1.95082 | -0.07596 |
| H | -1.63439 | 1.04851 | -0.38812 |
| H | 3.33804 | -2.20899 | 0.42371 |
| H | 1.88986 | -1.92106 | 1.37918 |
| H | 2.19380 | -1.40489 | -1.62524 |
| H | 1.16961 | -2.61298 | -0.83230 |
| C | 3.10243 | 2.20097 | -0.31045 |
| H | 3.40344 | 2.76462 | 0.58272 |
| H | 4.02659 | 1.91546 | -0.82942 |
| H | 2.52691 | 2.86738 | -0.95936 |
| C | -3.24856 | -0.32017 | 0.16646 |
| C | -4.08287 | 0.70287 | 0.91480 |
| C | -4.37068 | 0.42985 | -0.52719 |
| H | -3.44419 | -1.36302 | 0.40420 |
| H | -3.60325 | 1.64381 | 1.17065 |
| H | -4.77522 | 0.32595 | 1.66171 |
| H | -5.26303 | -0.13570 | -0.77873 |
| H | -4.08791 | 1.18472 | -1.25587 |

**Methyl acrylate**

***E*** = -306.4619038

***G*** = -306.3953838

$\boldsymbol{N_{imag}}$ = 0

| | | | |
|---|---|---|---|
| C | 0.10606 | -1.98124 | 1.93265 |
| H | -0.14836 | -1.94296 | 2.98765 |
| C | 1.36494 | -1.82961 | 1.52442 |
| H | 2.18071 | -1.66219 | 2.22105 |
| C | 1.78431 | -1.83915 | 0.10420 |
| O | 2.92148 | -1.62737 | -0.25923 |
| H | -0.70230 | -2.14032 | 1.22842 |
| O | 0.77577 | -2.10594 | -0.74084 |
| C | 1.10227 | -1.99601 | -2.12165 |
| H | 1.89022 | -2.70330 | -2.39240 |
| H | 0.18197 | -2.22260 | -2.66019 |
| H | 1.43778 | -0.98027 | -2.34818 |

**Crystallographic Data**

| | |
|---|---|
| Empirical formula | $C_{30}H_{33}NO_5$ |
| Formula weight | 487.57 |
| Temperature/K | 140 |
| Crystal system | monoclinic |
| Space group | $P2_1/c$ |
| a/Å | 17.4594(8) |
| b/Å | 6.5351(3) |
| c/Å | 23.4664(11) |
| $\alpha$/° | 90 |
| $\beta$/° | 106.654(5) |
| $\gamma$/° | 90 |
| $V$/Å$^3$ | 2565.2(2) |
| $Z$ | 4 |
| $r_{calc}$ g/cm$^3$ | 1.262 |
| $m$/mm$^{-1}$ | 0.085 |
| $F$(000) | 1040.0 |
| Crystal size/mm$^3$ | 0.27 × 0.16 × 0.12 |
| Radiation | Mo Ka (λ = 0.71073) |
| 2$q$ range for data collection/° | 4.87 to 59.404 |

Index ranges −23 ≤ h ≤ 20, −8 ≤ k ≤ 8, −32 ≤ l ≤ 31

Reflections collected 23862

Independent reflections 6060 [$R_{int}$ = 0.0491, $R_{sigma}$ = 0.0412]

Data/restraints/parameters 6060/0/327

Goodness−of−fit on $F^2$ 1.105

Final $R$ indexes [$I$>=2σ ($I$)] $R_1$ = 0.1099, $wR_2$ = 0.2910

Final $R$ indexes [all data] $R_1$ = 0.1230, $wR_2$ = 0.2968

Largest diff. peak/hole / e Å$^{-3}$ 0.40/−0.37

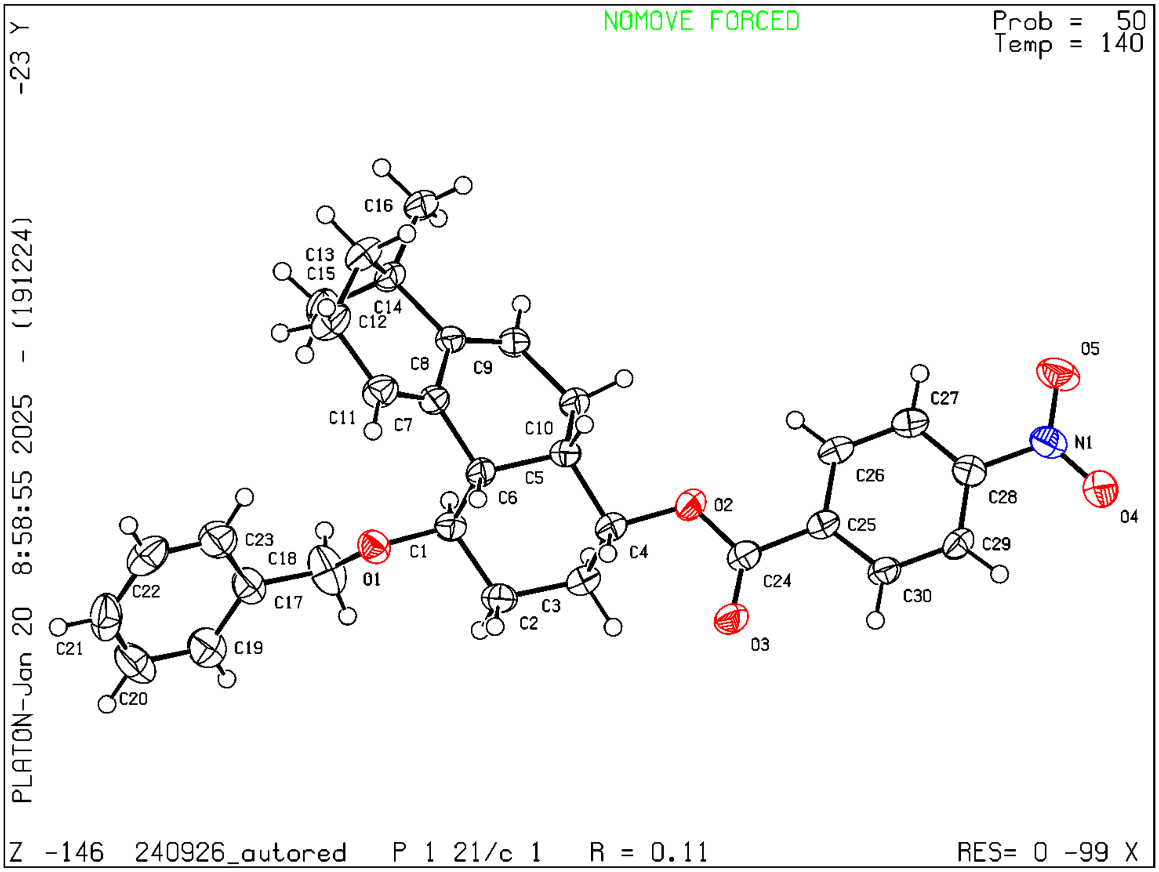

# $^{1}H$ and $^{13}C$ NMR Spectra of New Compounds

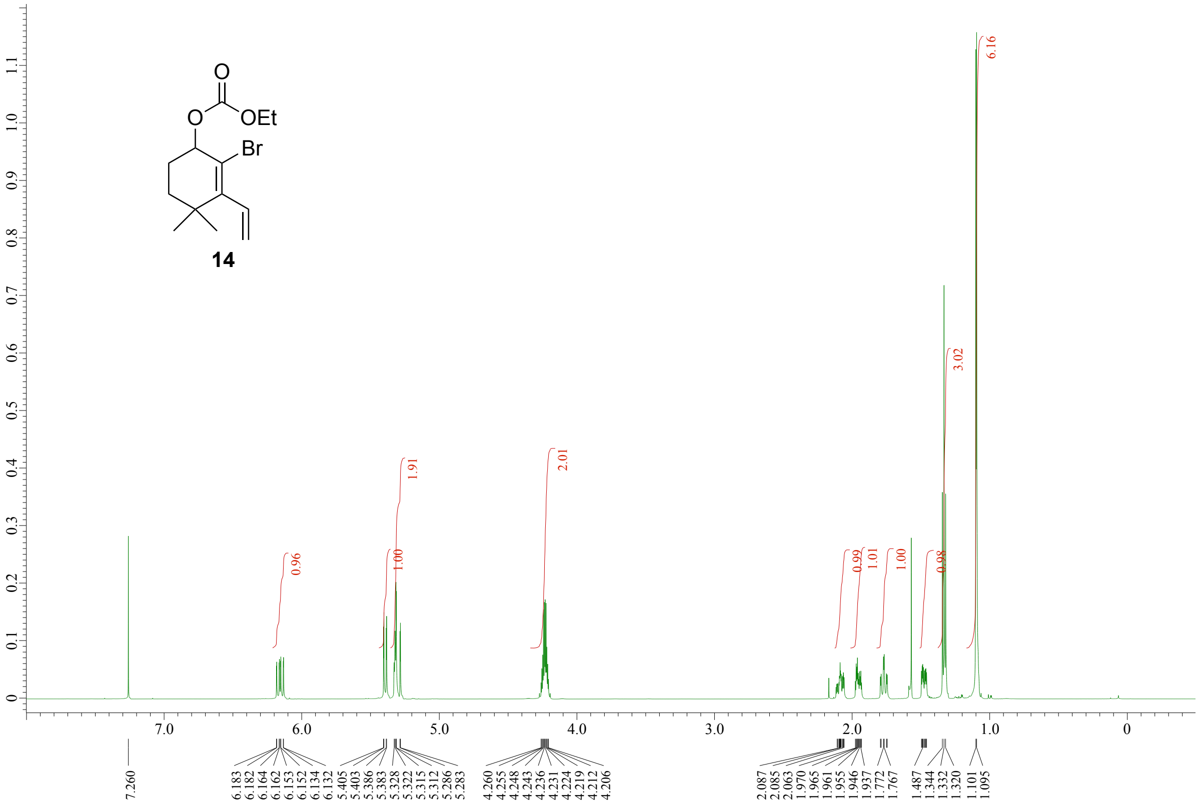

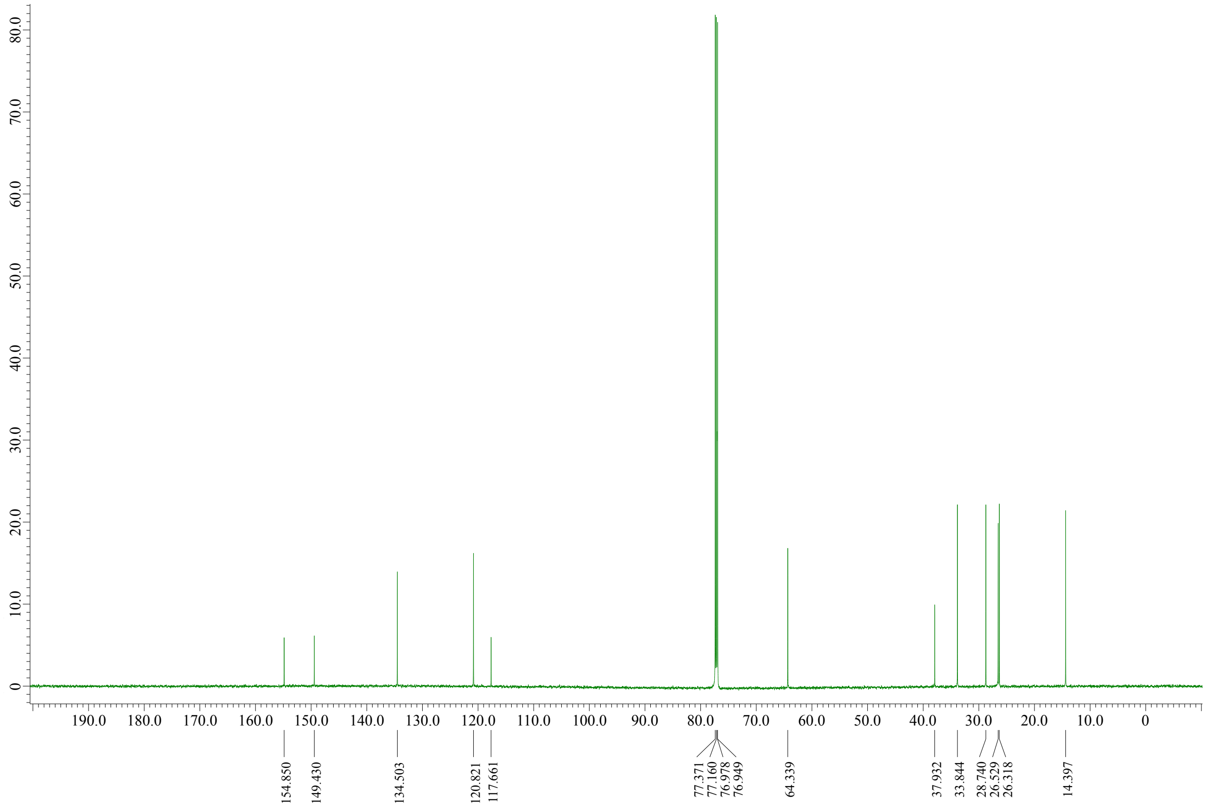

**Figure S5.** $^{1}H$ and $^{13}C$ NMR Spectra of **14** ($CDCl_3$)

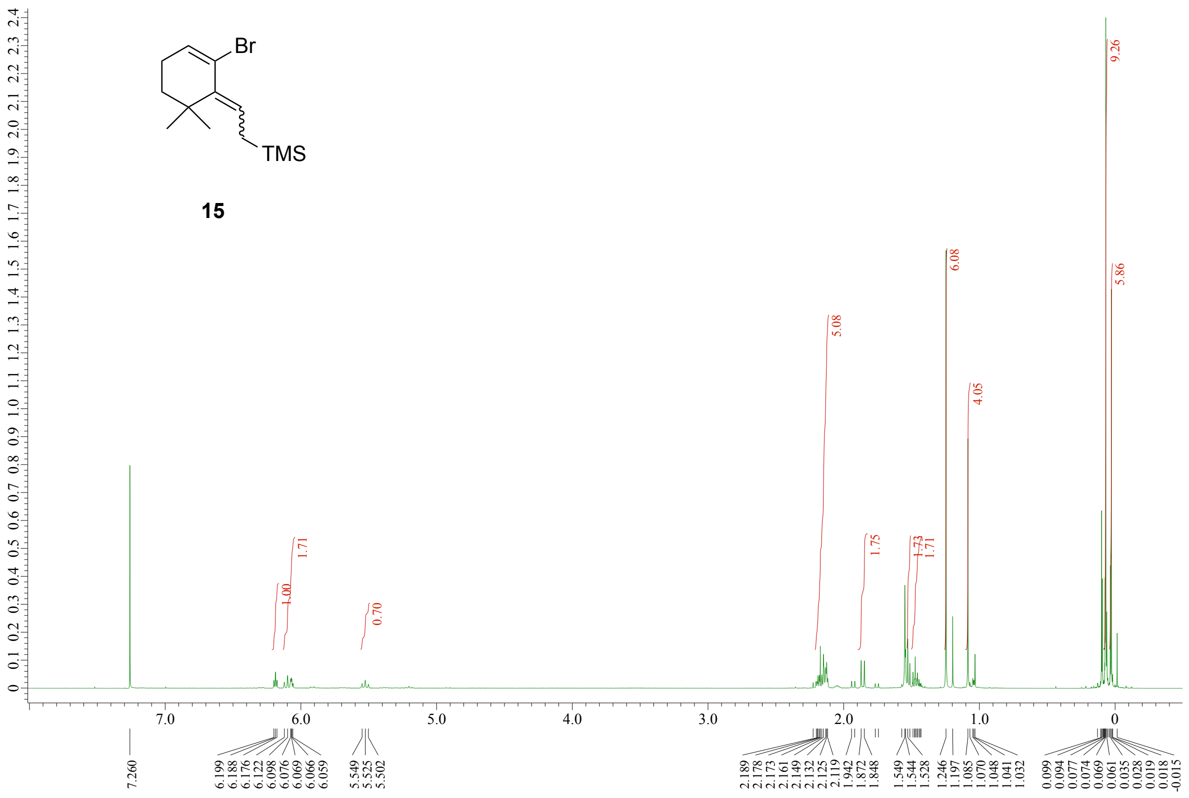

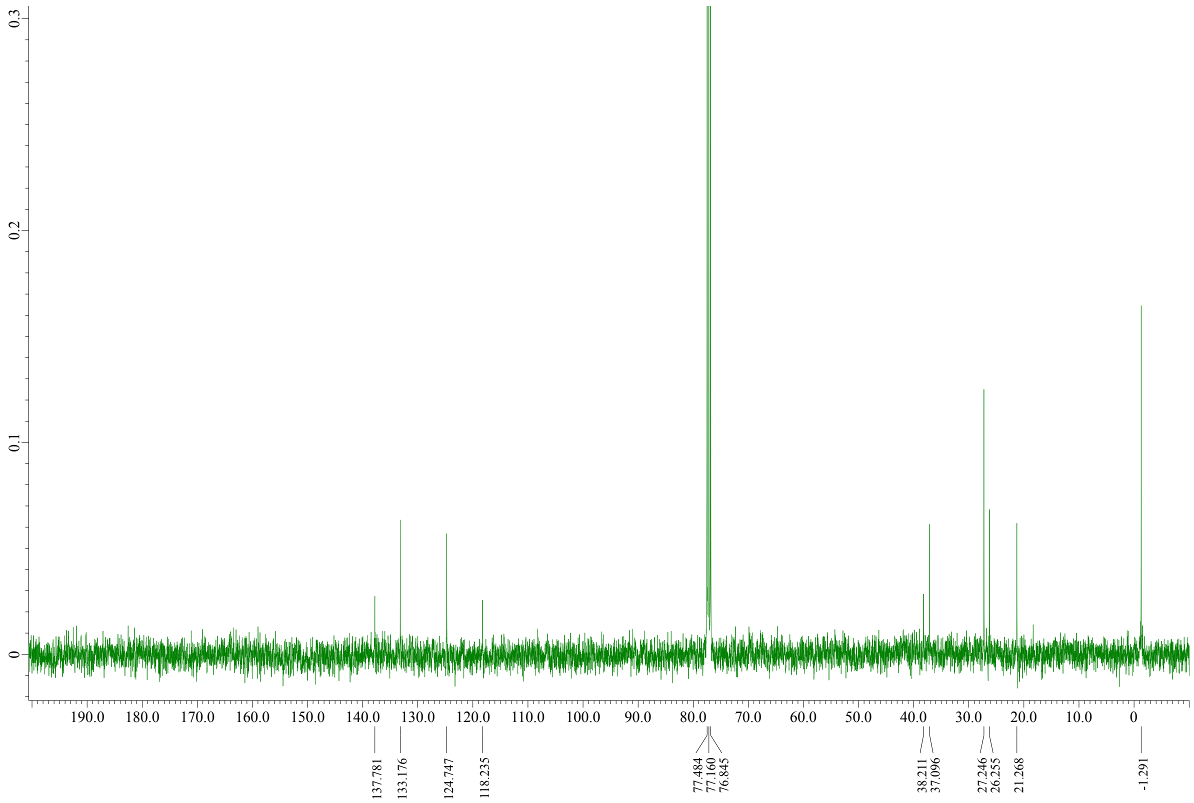

**Figure S6.** $^{1}H$ and $^{13}C$ NMR Spectra of **15** ($CDCl_3$)

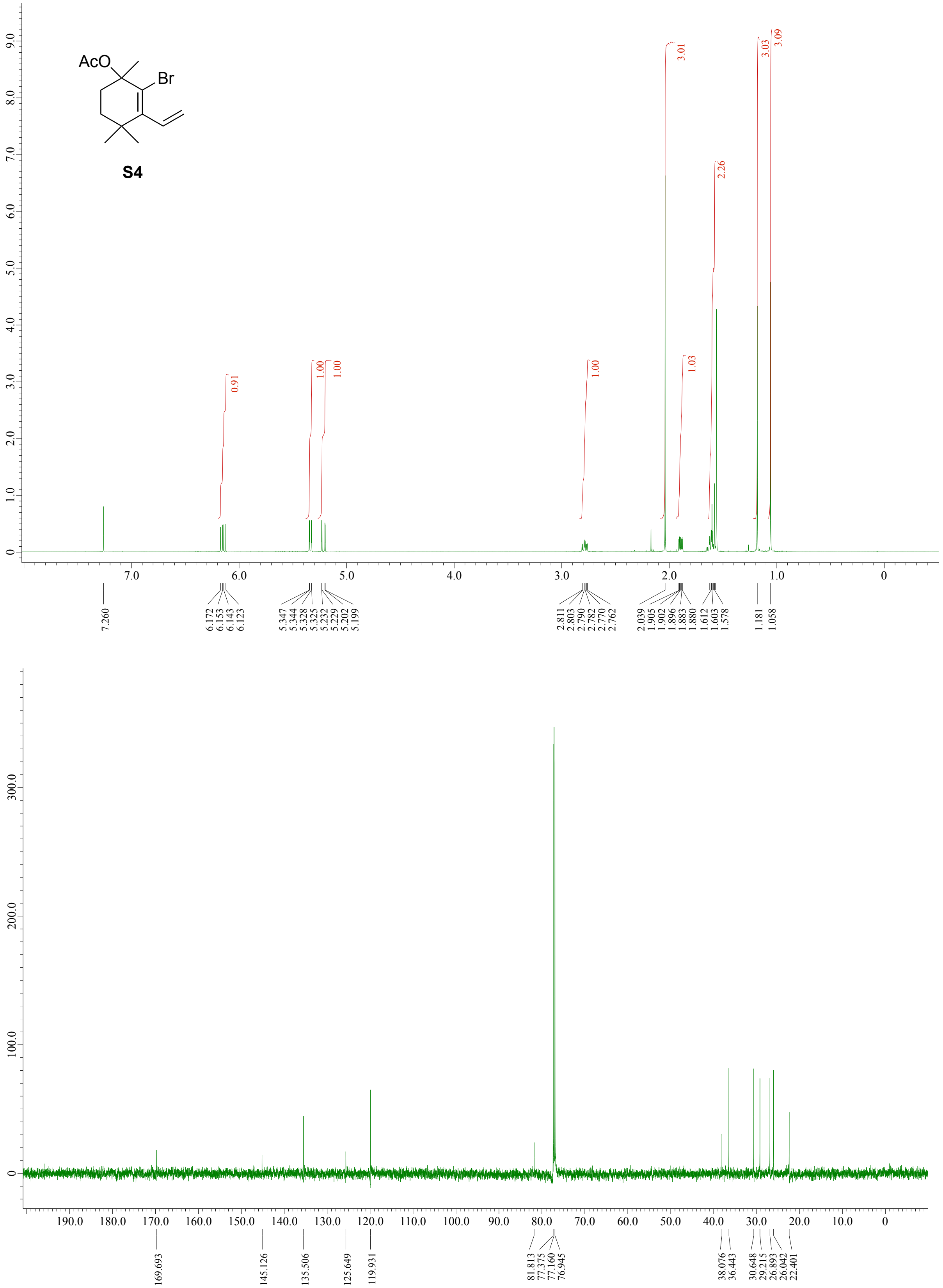


**Figure S7.** $^{1}H$ and $^{13}C$ NMR Spectra of **S4** ($CDCl_3$)

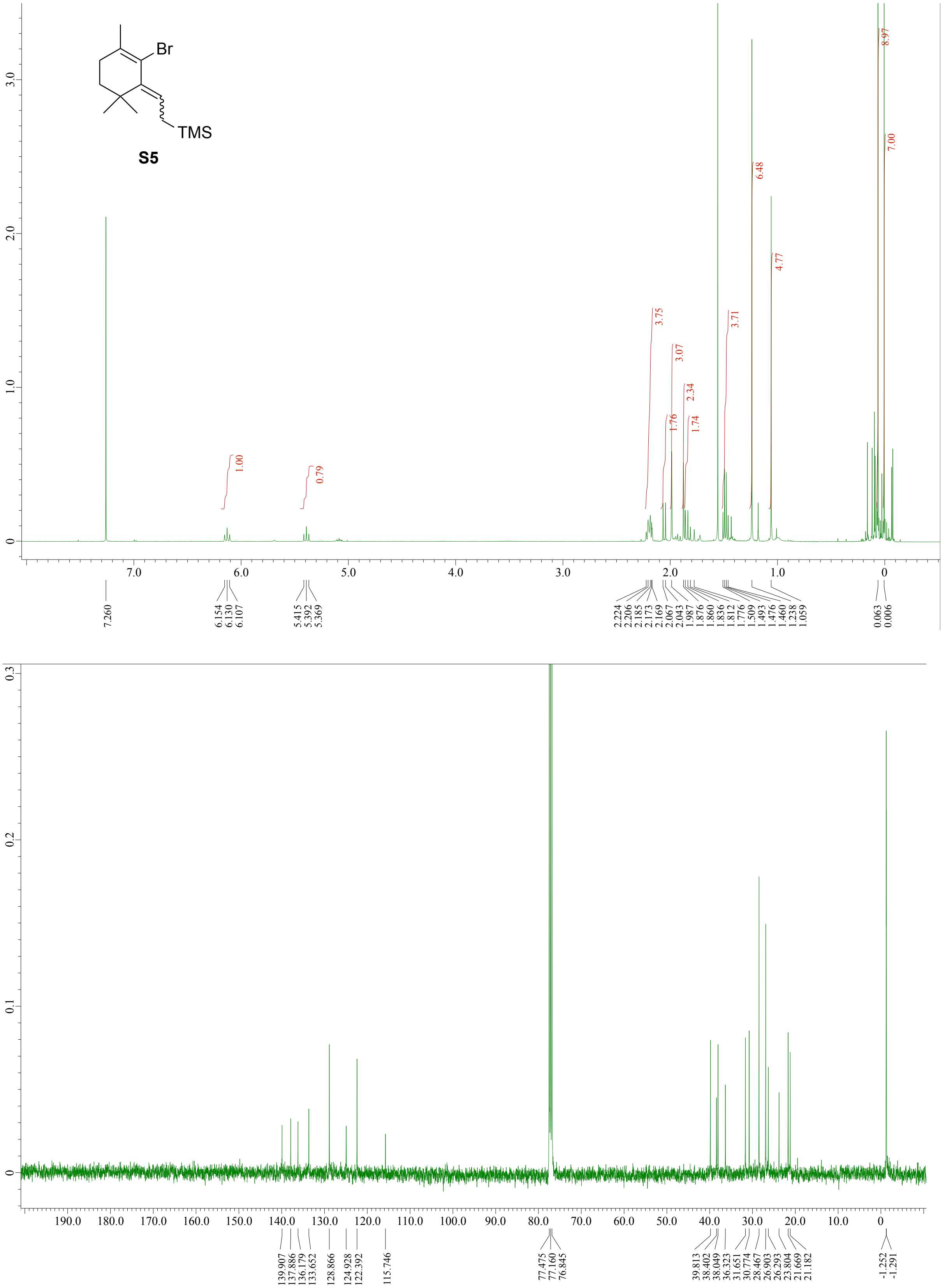


**Figure S8.** $^{1}H$ and $^{13}C$ NMR Spectra of **S5** ($CDCl_3$)

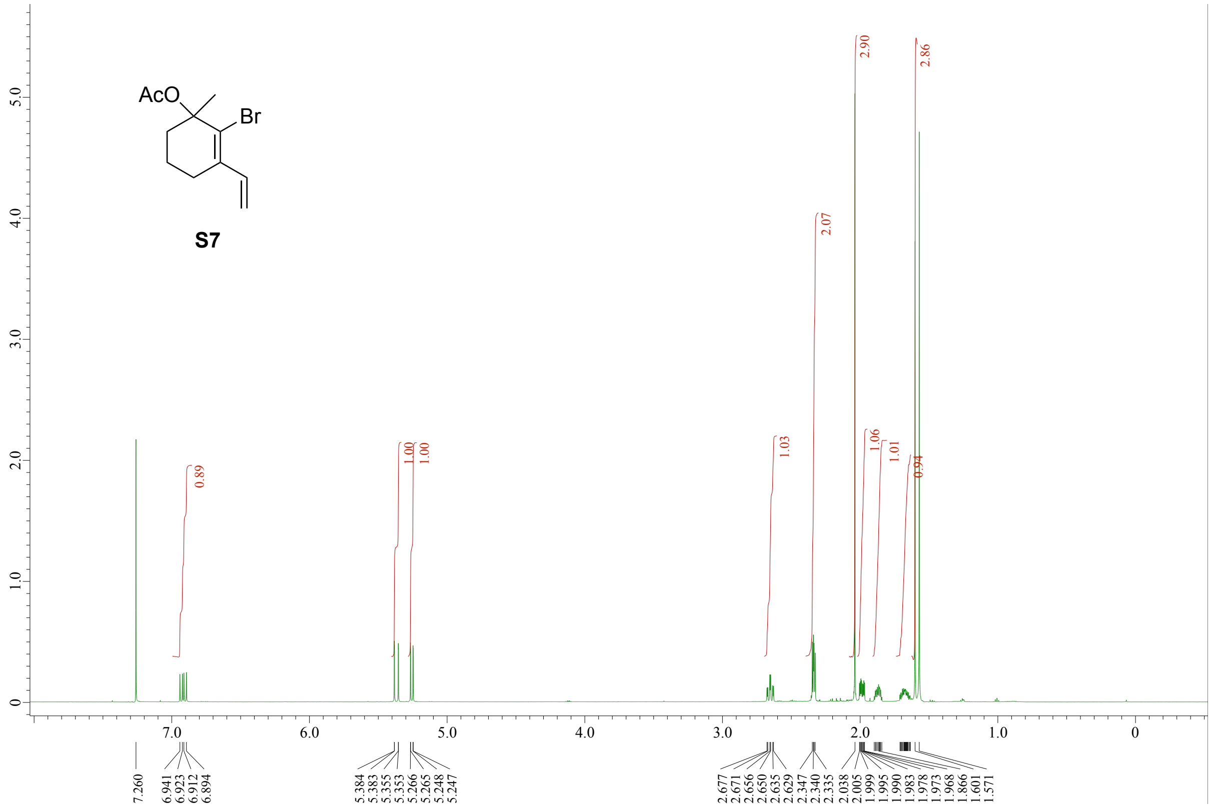


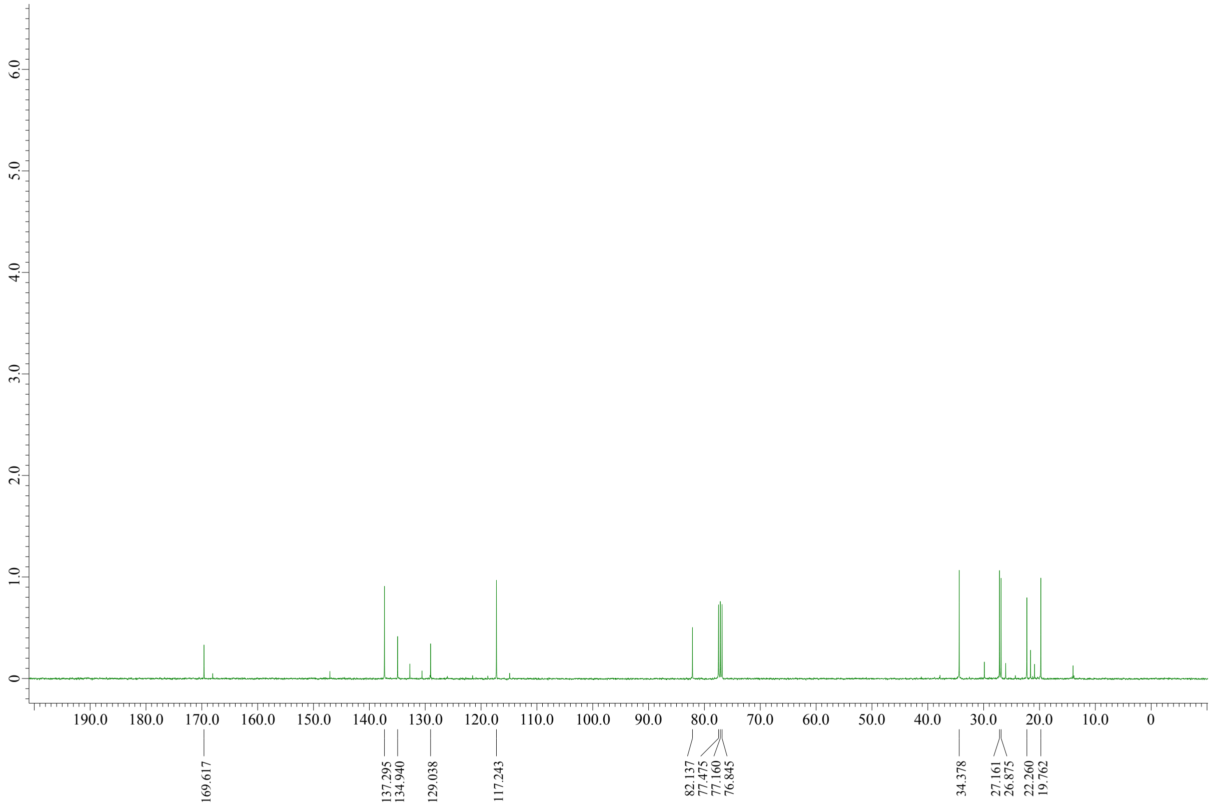


**Figure S9.** $^{1}H$ and $^{13}C$ NMR Spectra of **S7** ($CDCl_3$)

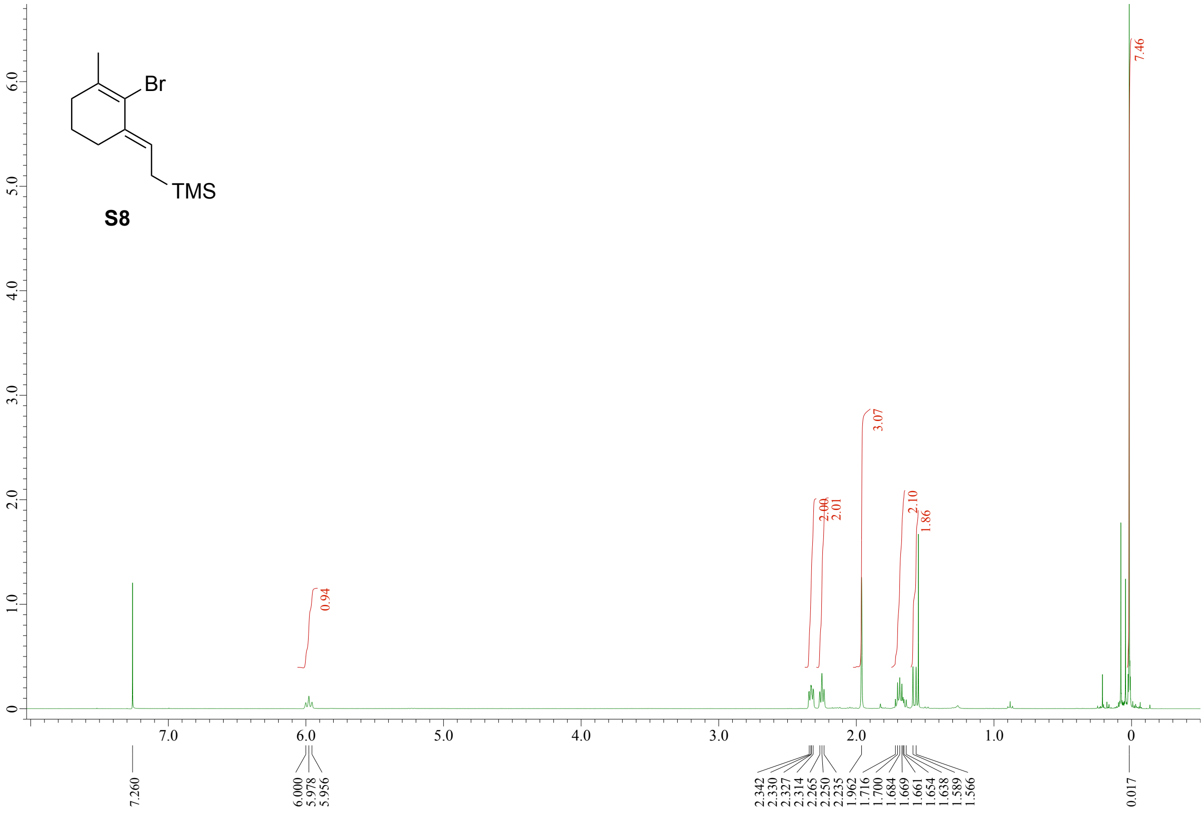


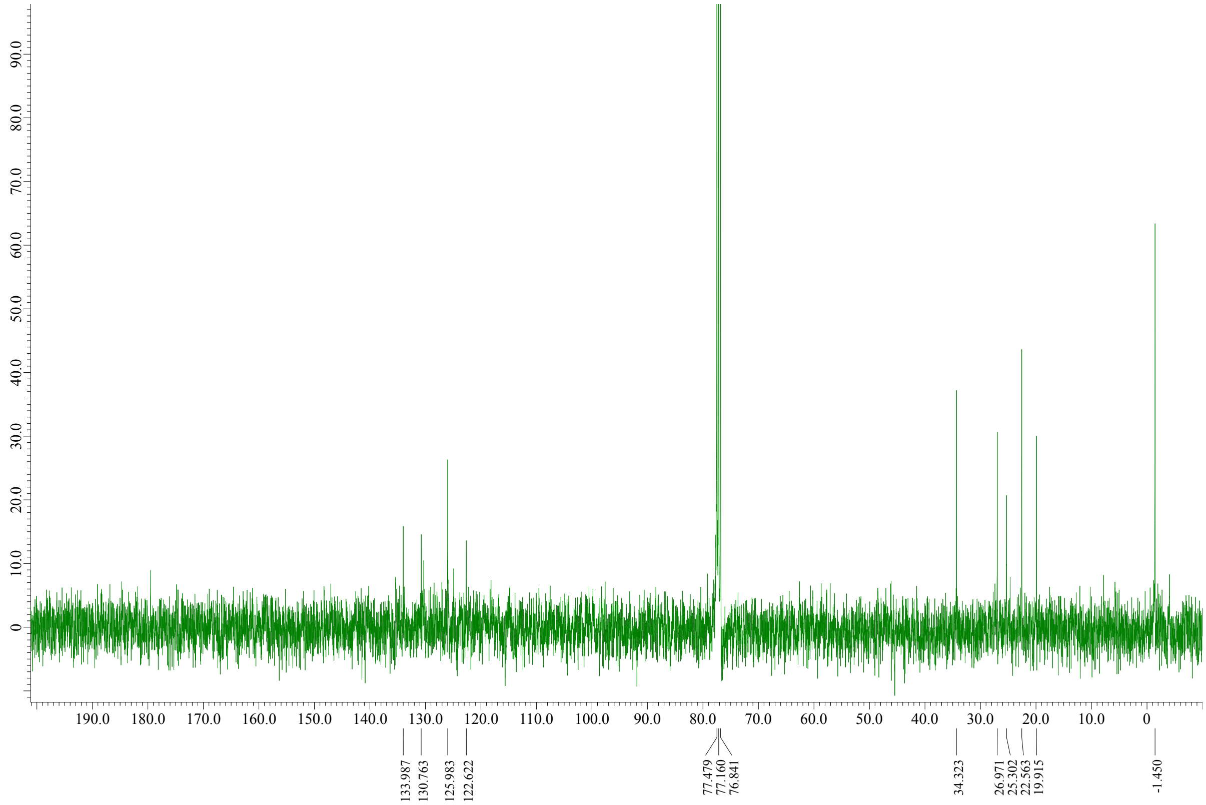


**Figure S10.** $^{1}H$ and $^{13}C$ NMR Spectra of **S8** ($CDCl_3$)

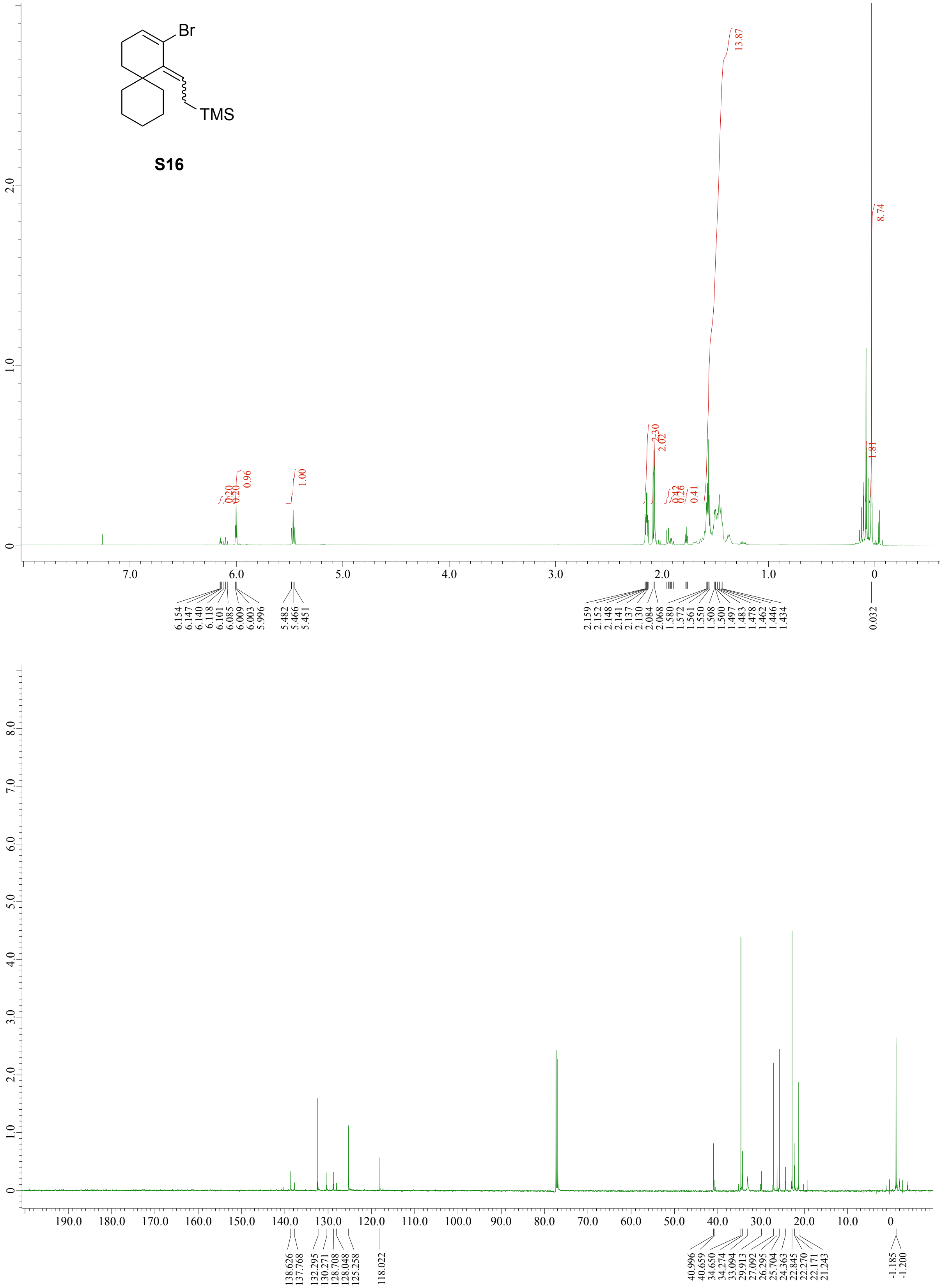

**Figure S11.** $^{1}$H and $^{13}$C NMR Spectra of **S16** ($CDCl_3$)

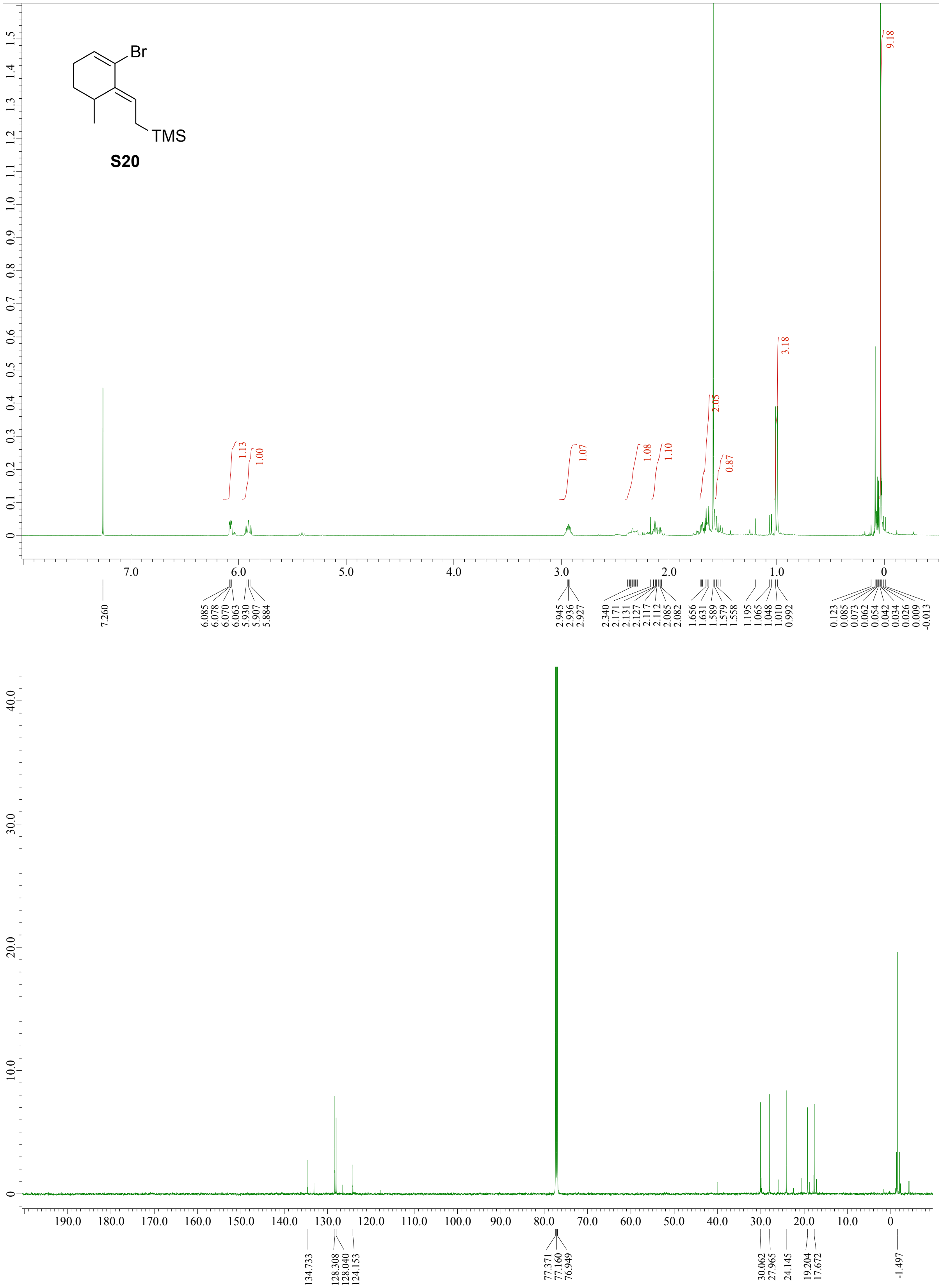


**Figure S12.** $^{1}H$ and $^{13}C$ NMR Spectra of **S20** ($CDCl_3$)

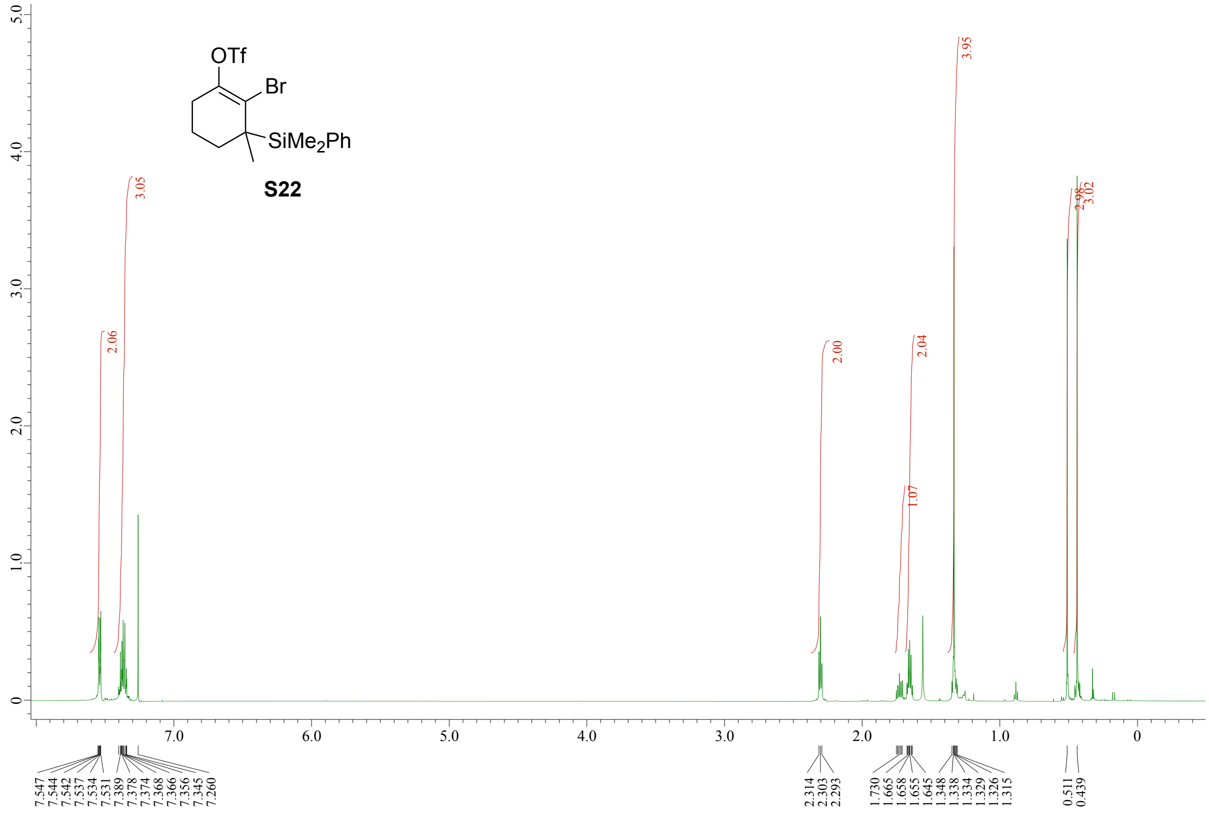


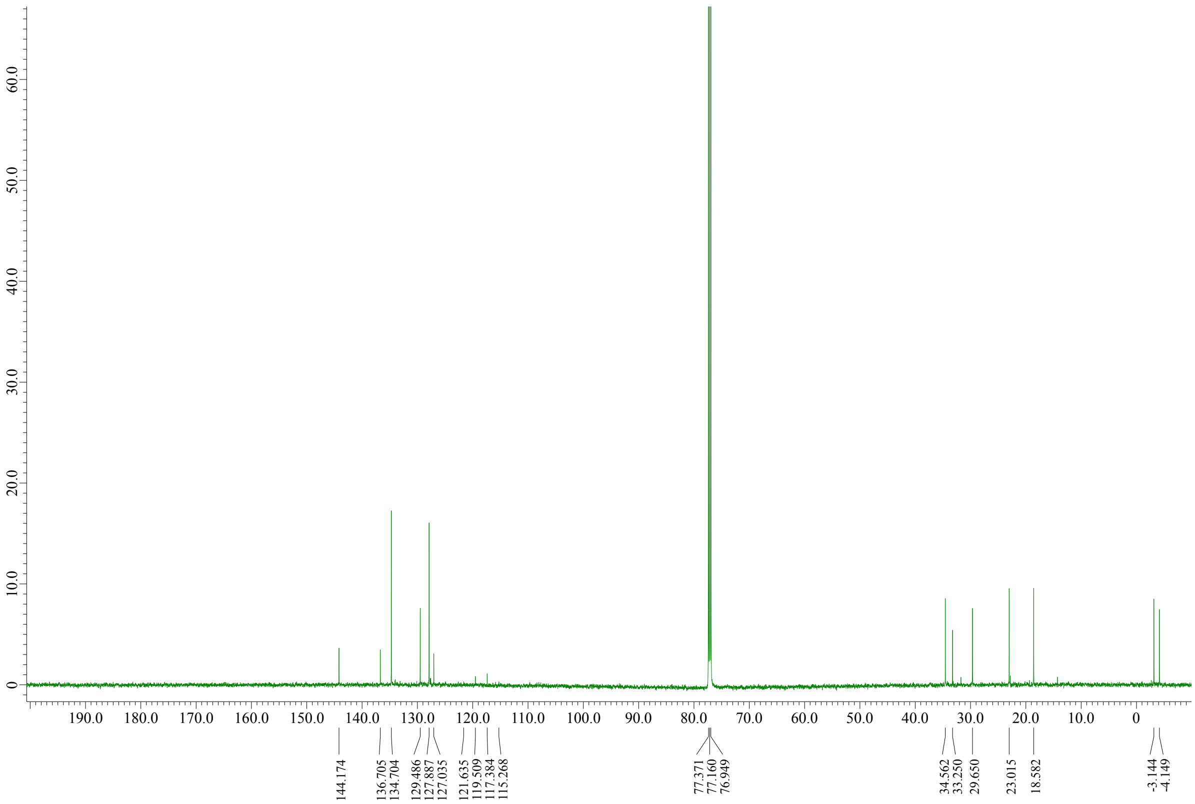


**Figure S13.** $^{1}H$ and $^{13}C$ NMR Spectra of **S22** ($CDCl_3$)

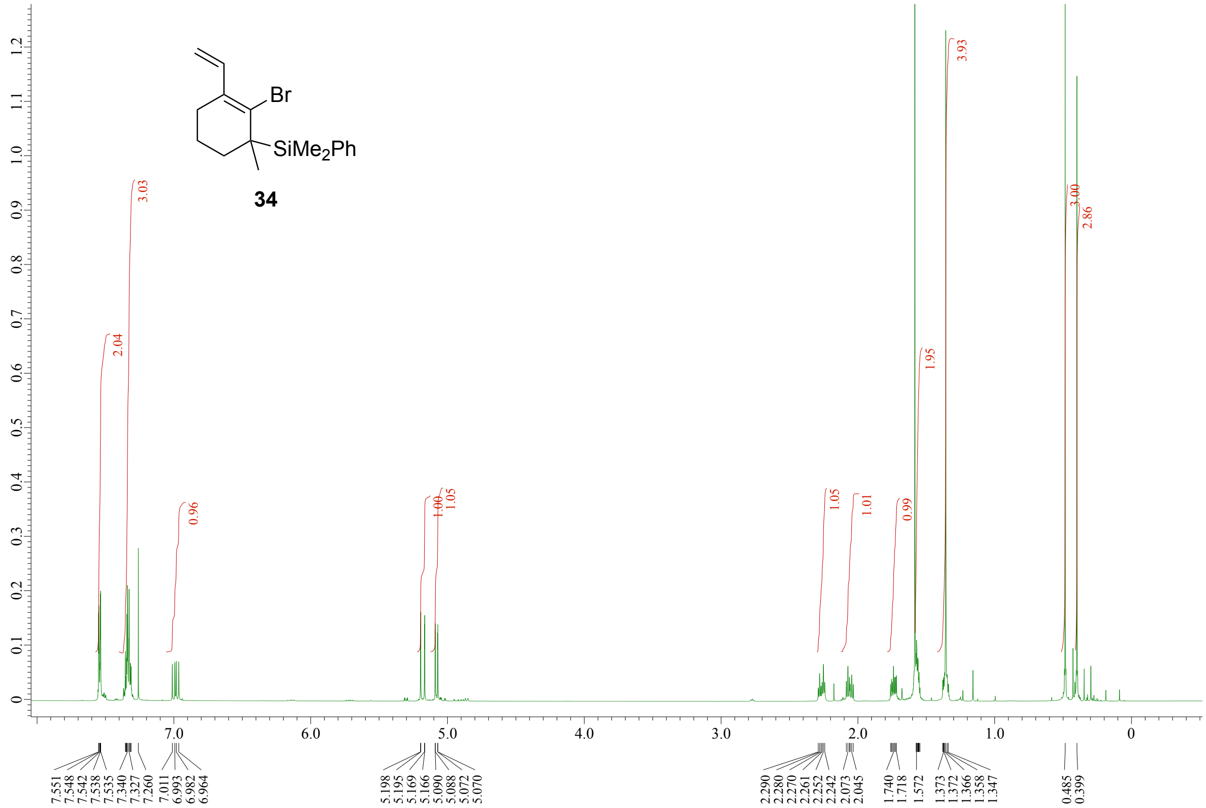


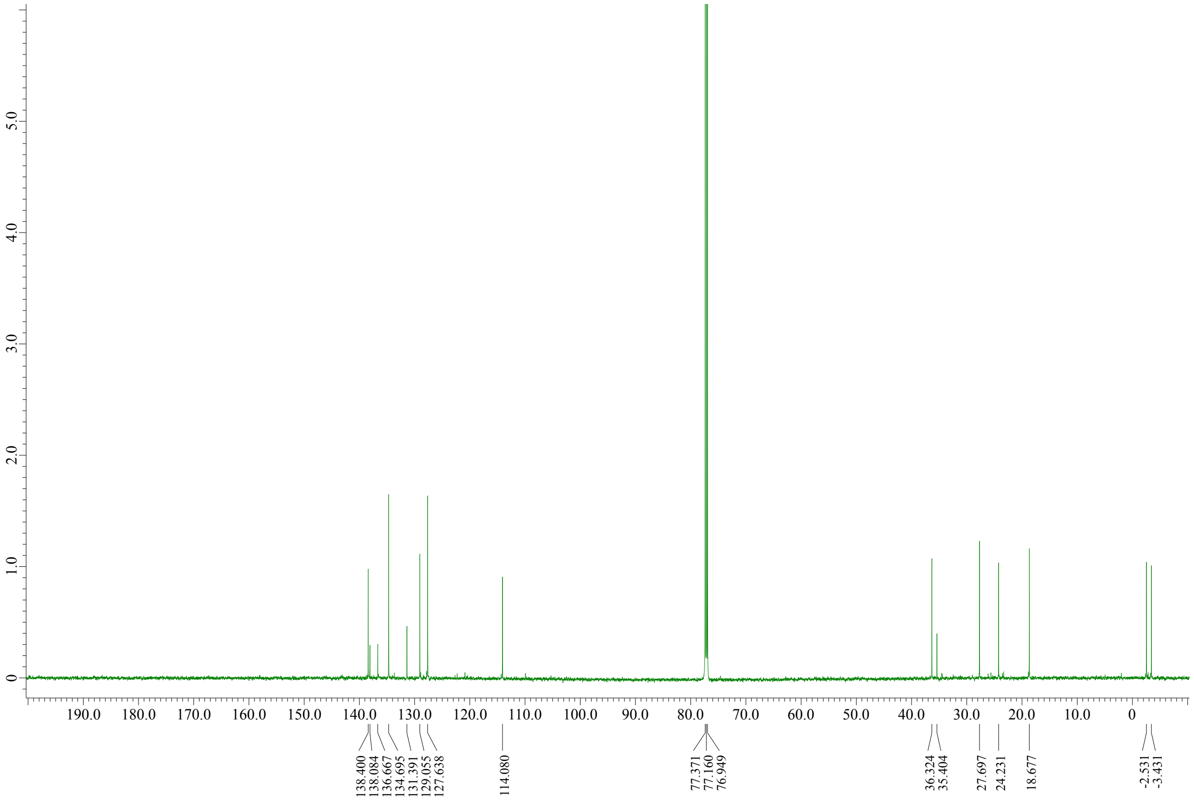


**Figure S14.** $^{1}H$ and $^{13}C$ NMR Spectra of **34** ($CDCl_3$)

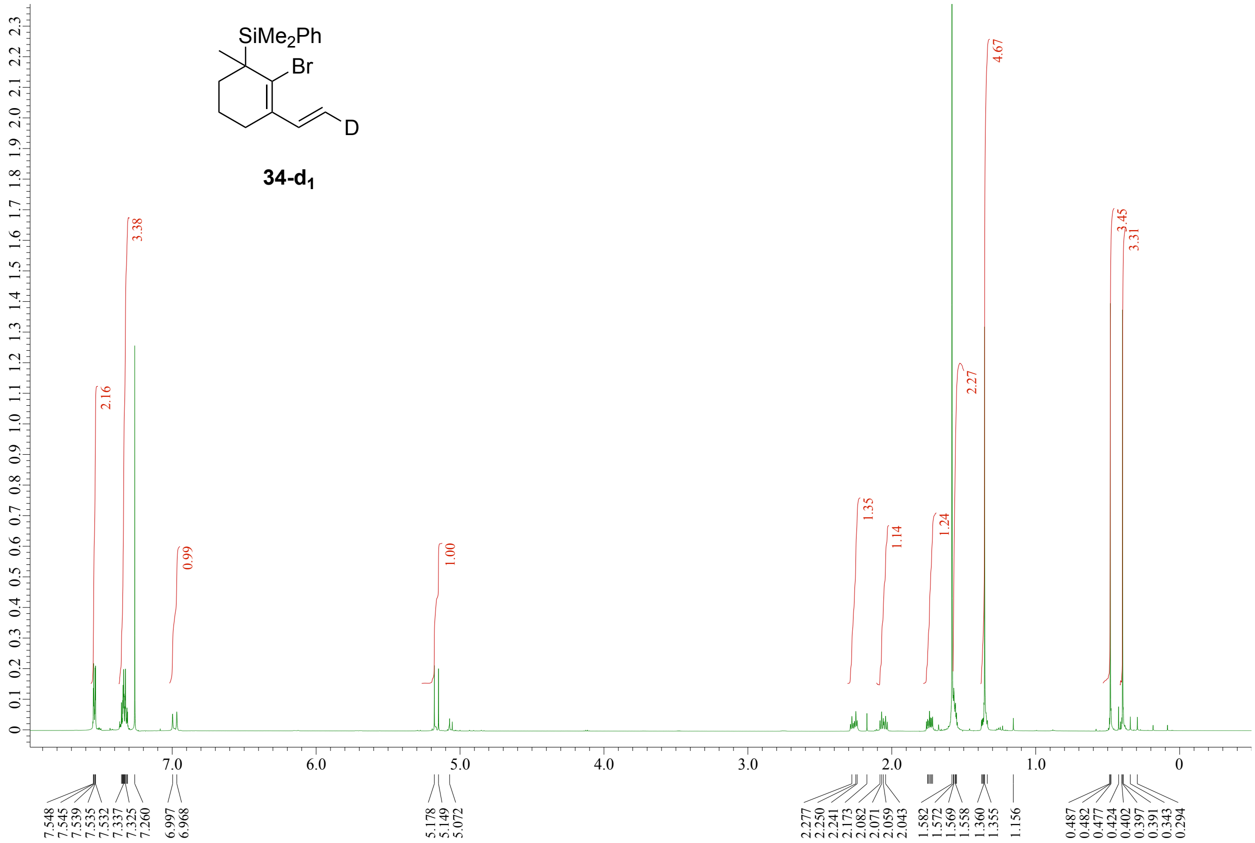


**Figure S15.** $^{1}$H Spectra of **34-d$_1$** ($CDCl_3$)

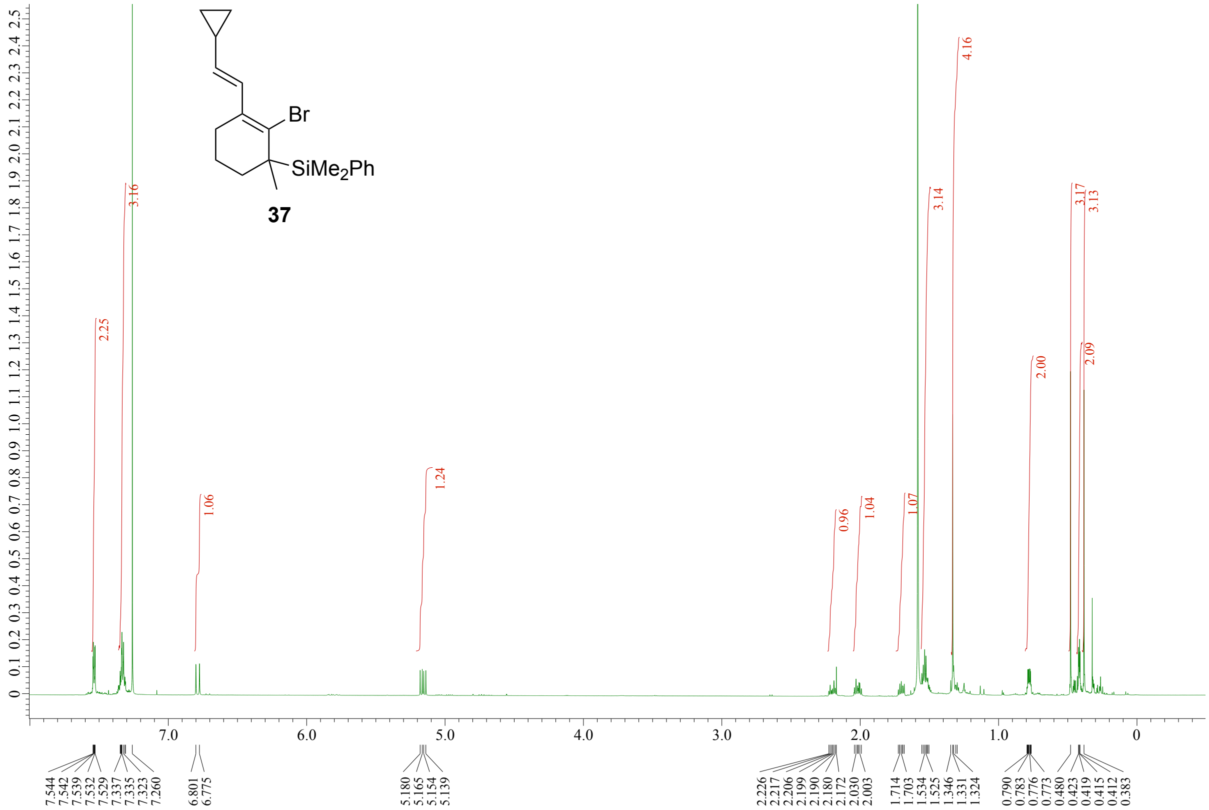


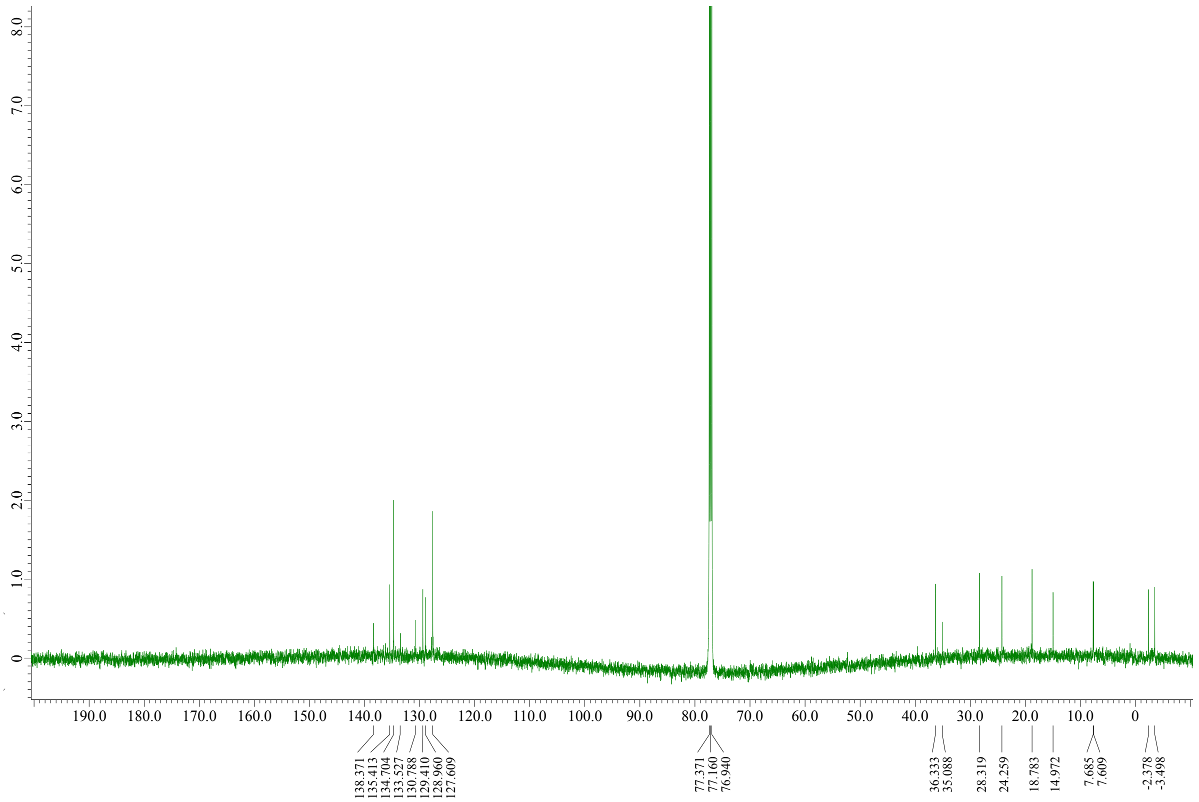


**Figure S16.** $^{1}$H and $^{13}$C NMR Spectra of **37** ($CDCl_3$)

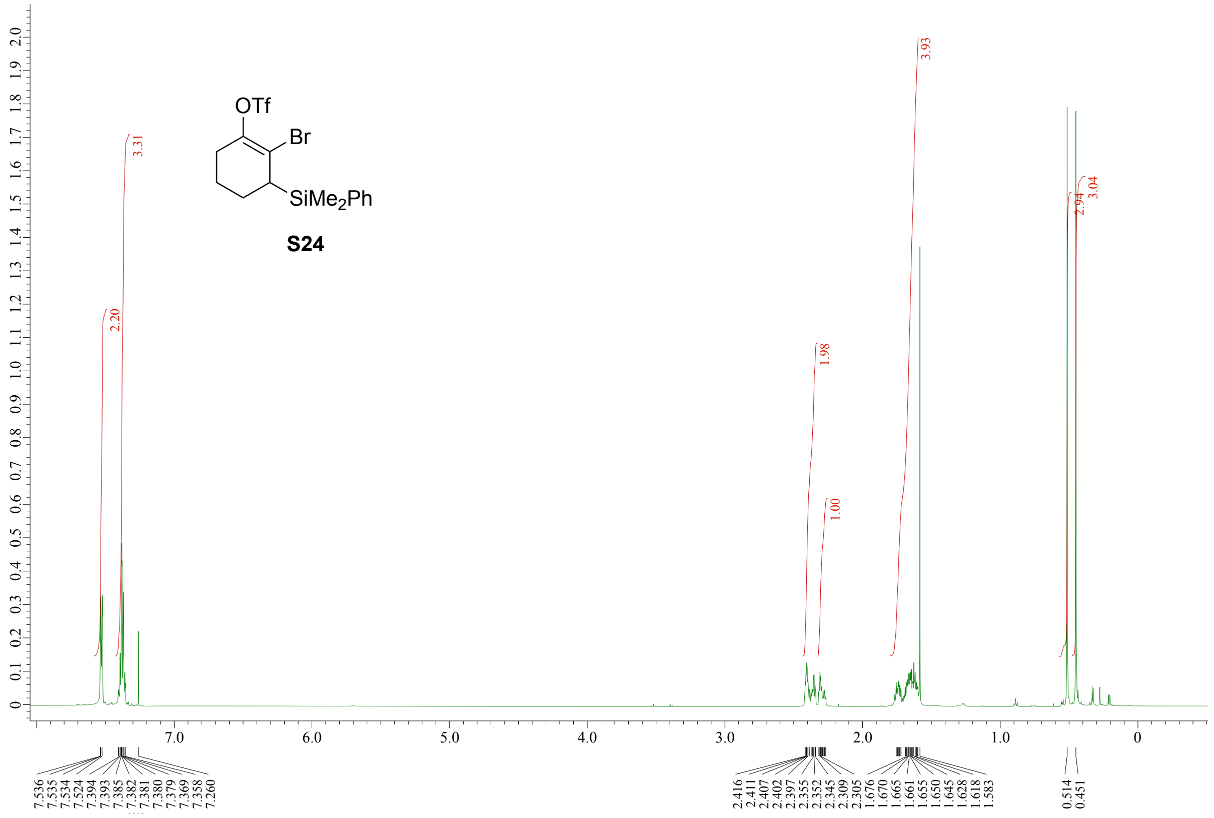

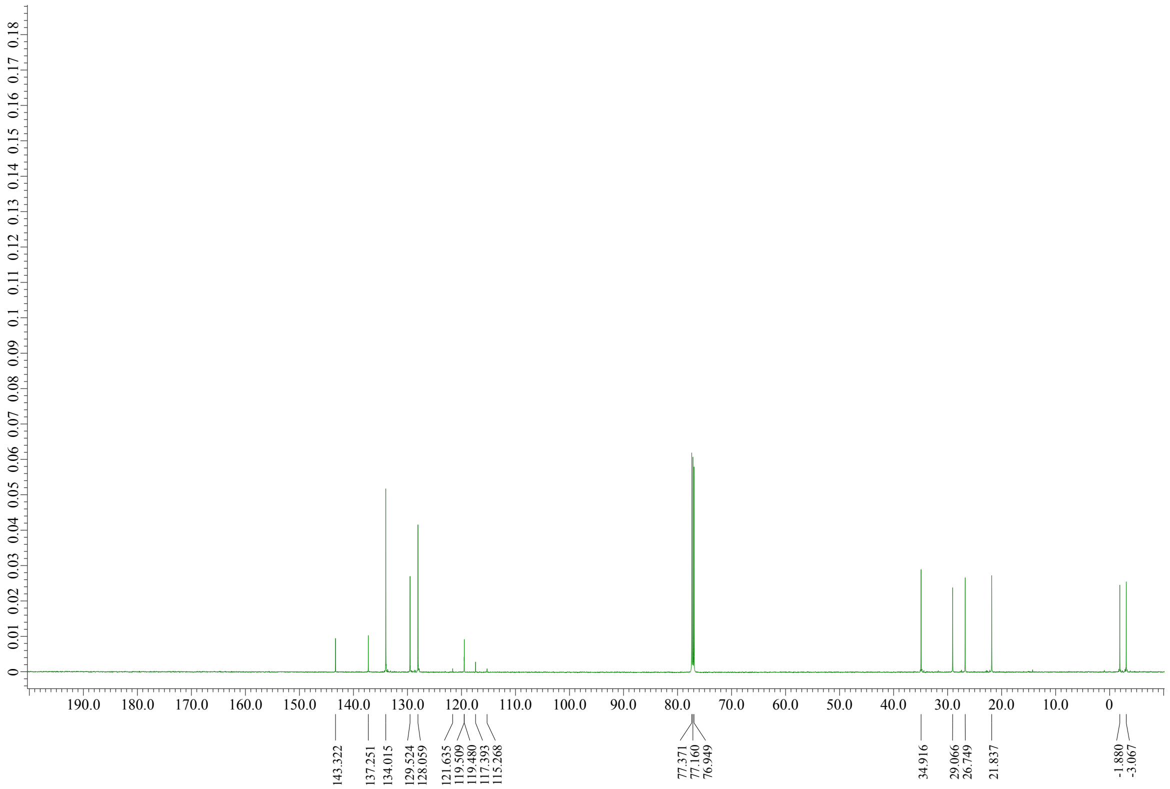

**Figure S17.** $^{1}H$ and $^{13}C$ NMR Spectra of **S24** ($CDCl_3$)

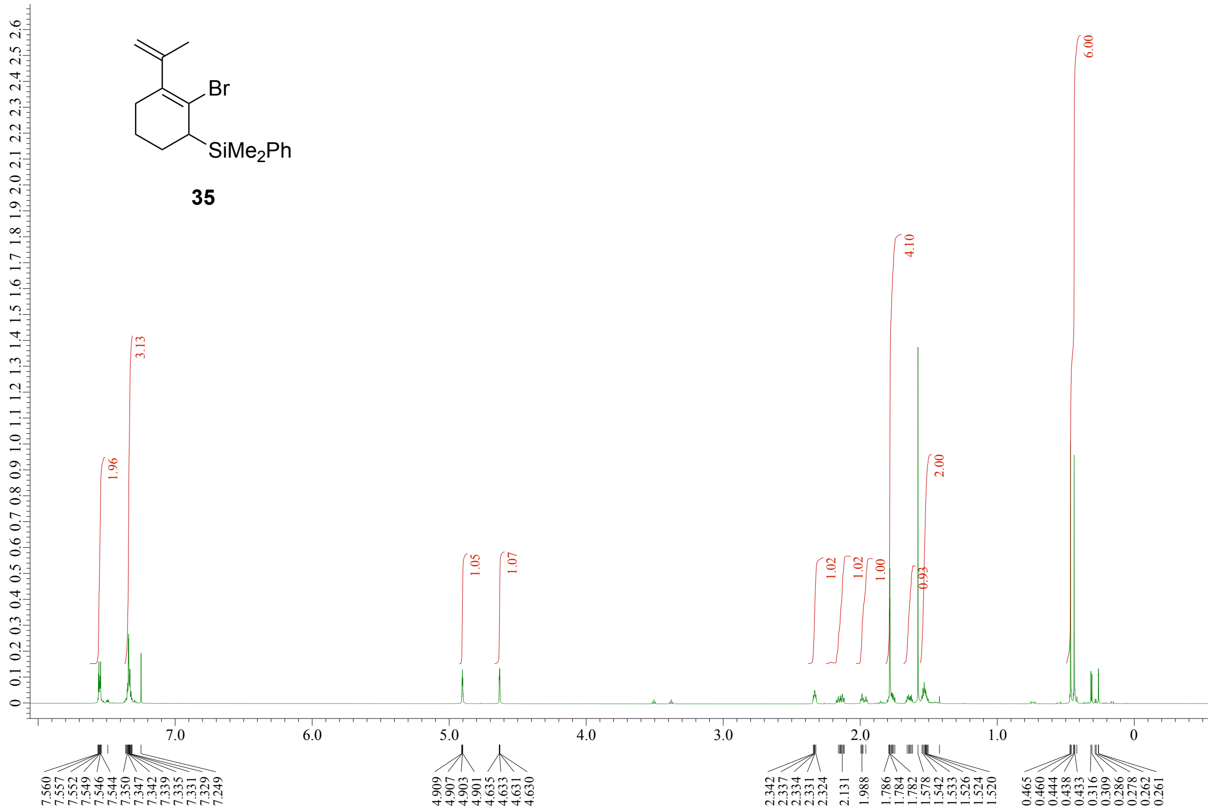


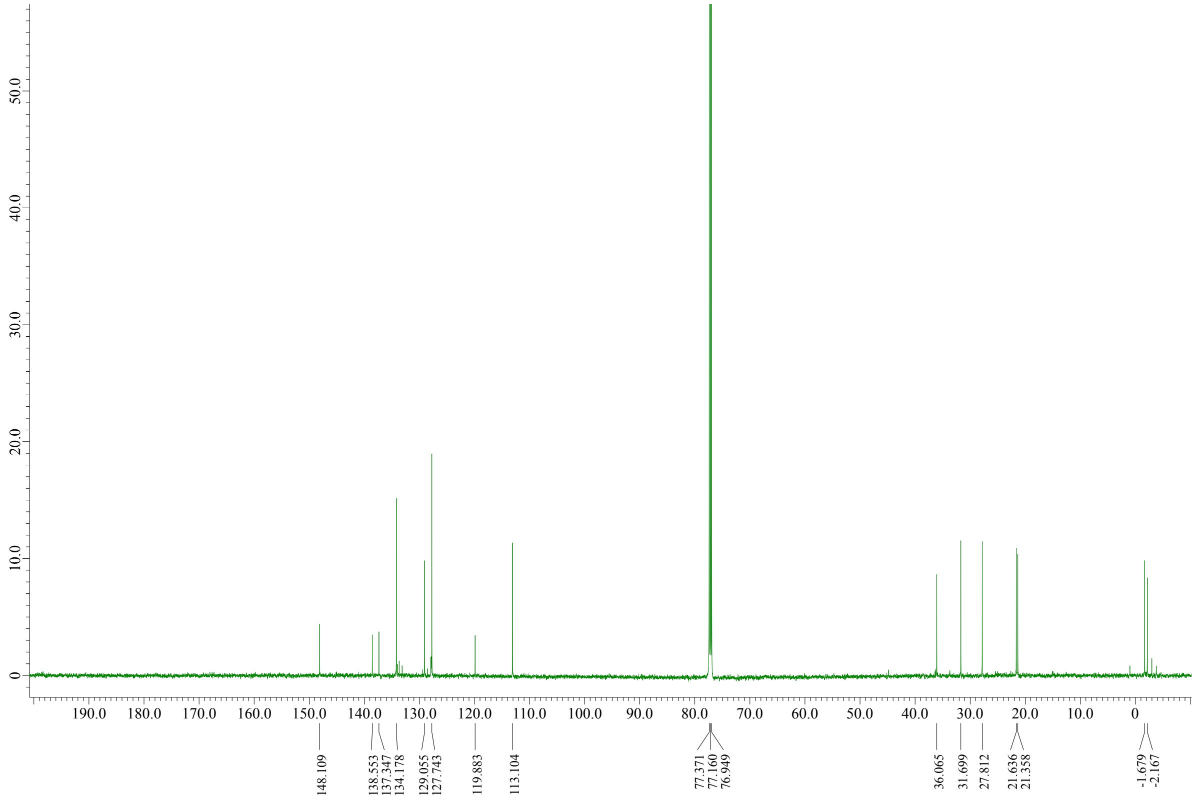


**Figure S18.** $^{1}H$ and $^{13}C$ NMR Spectra of **35** ($CDCl_3$)

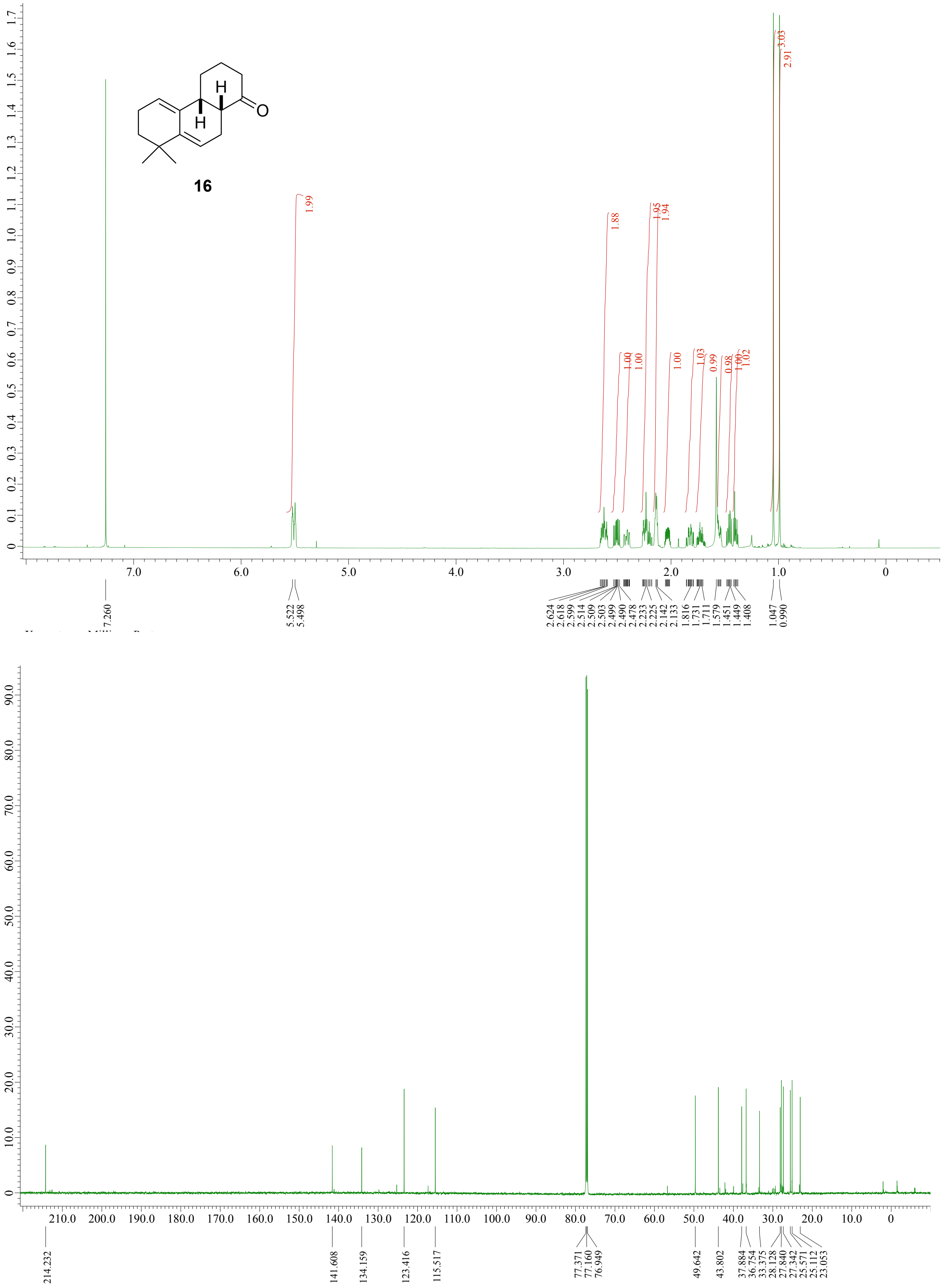


**Figure S19.** $^{1}H$ and $^{13}C$ NMR Spectra of **16** ($CDCl_3$)

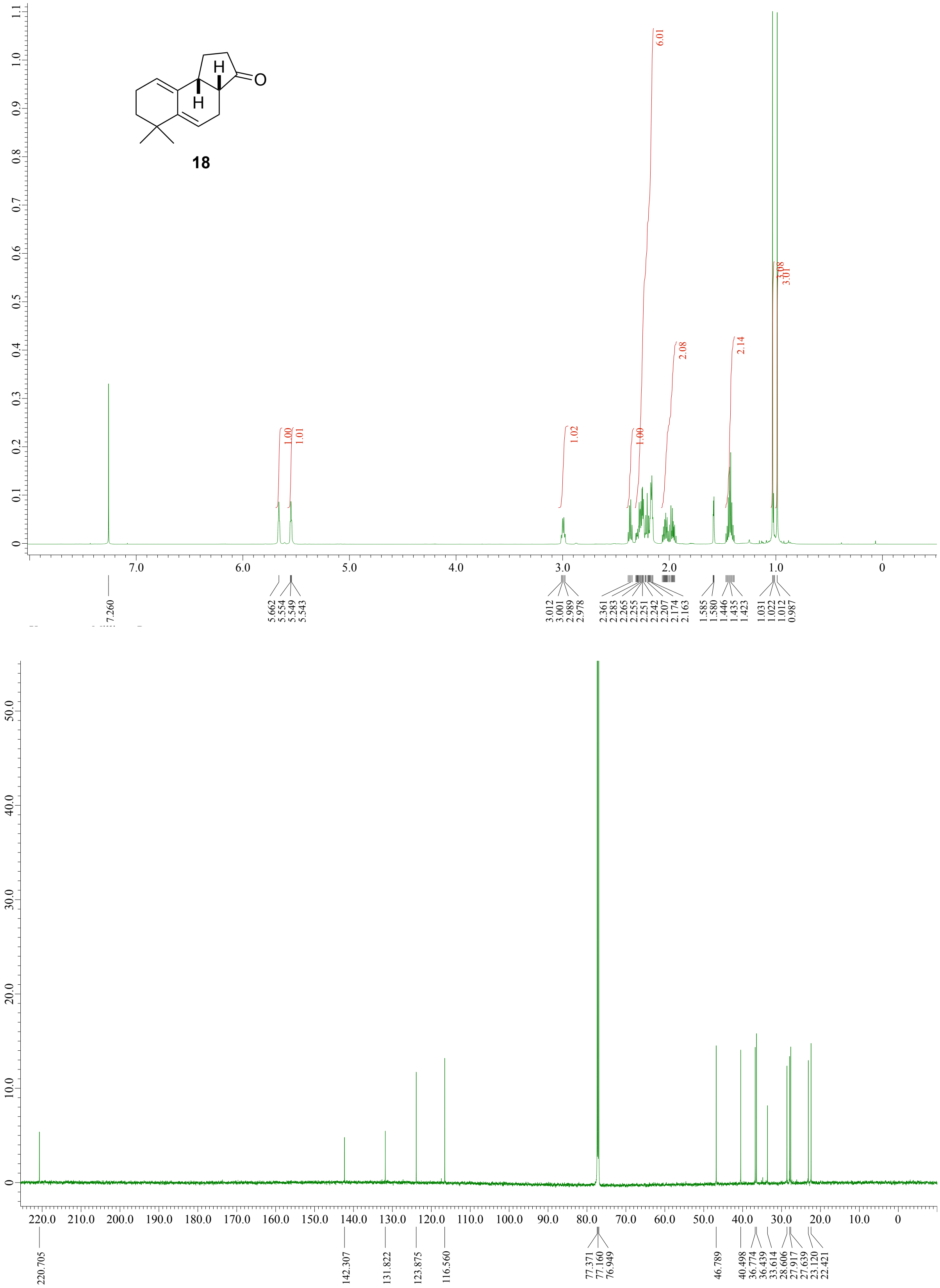


**Figure S20.** $^{1}H$ and $^{13}C$ NMR Spectra of **18** ($CDCl_3$)

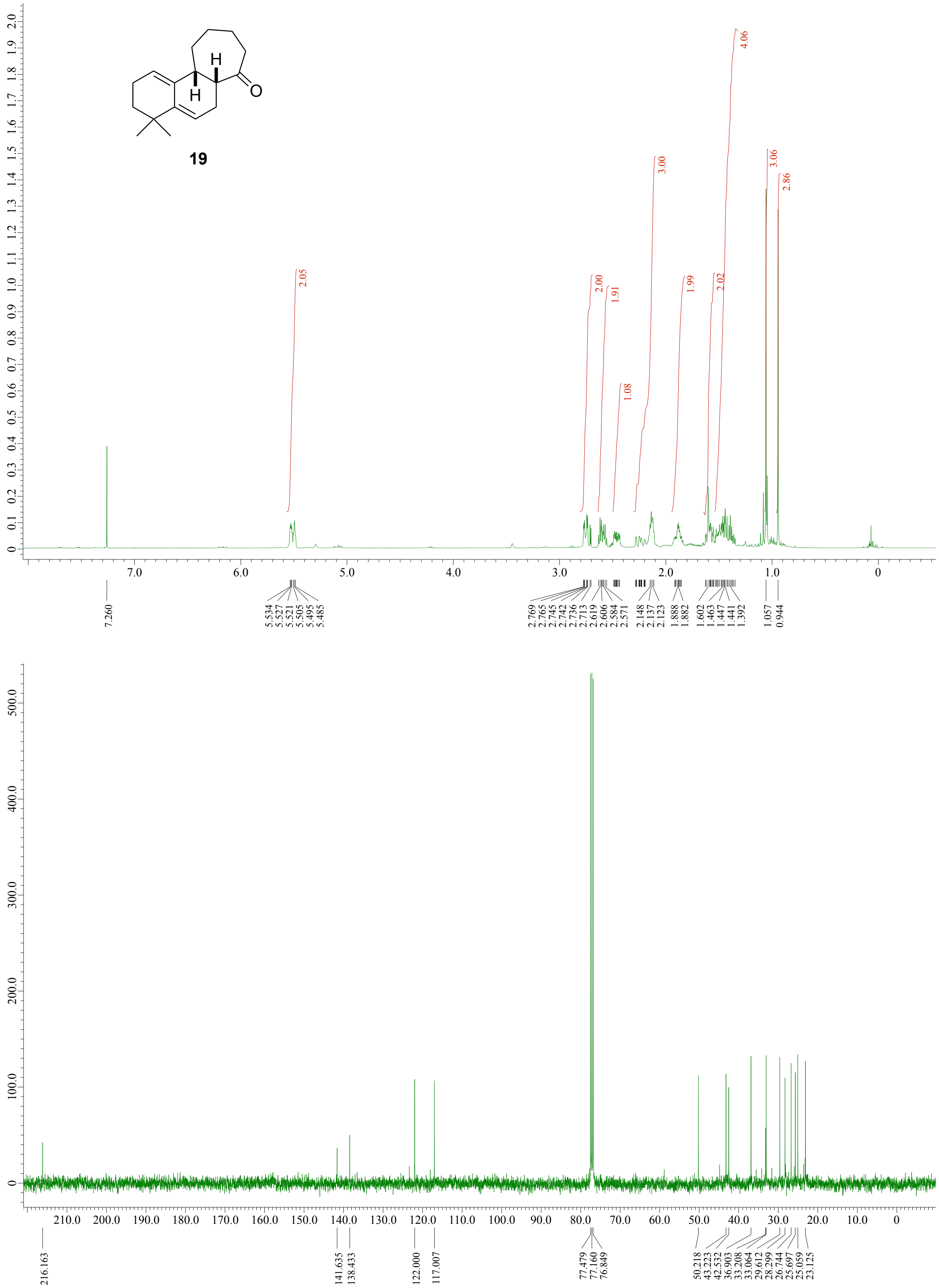


**Figure S21.** $^{1}H$ and $^{13}C$ NMR Spectra of **19** ($CDCl_3$)

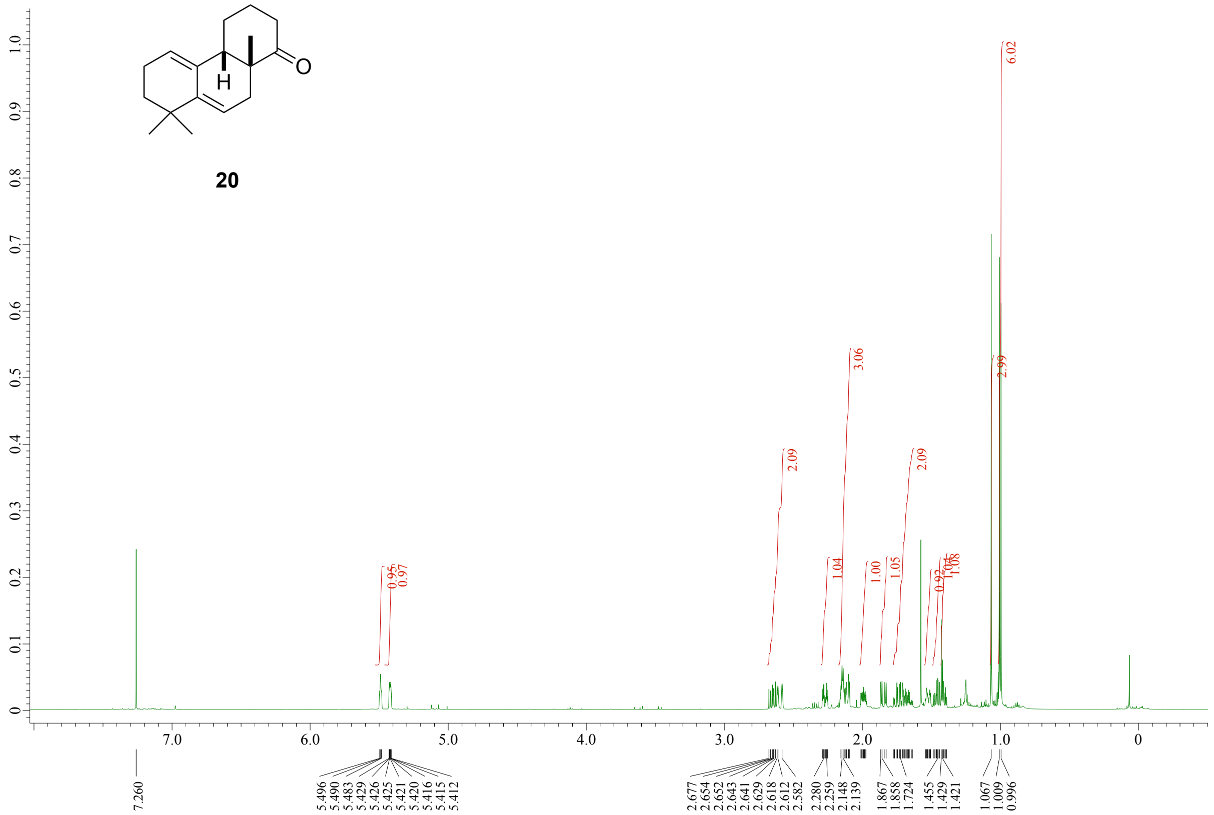


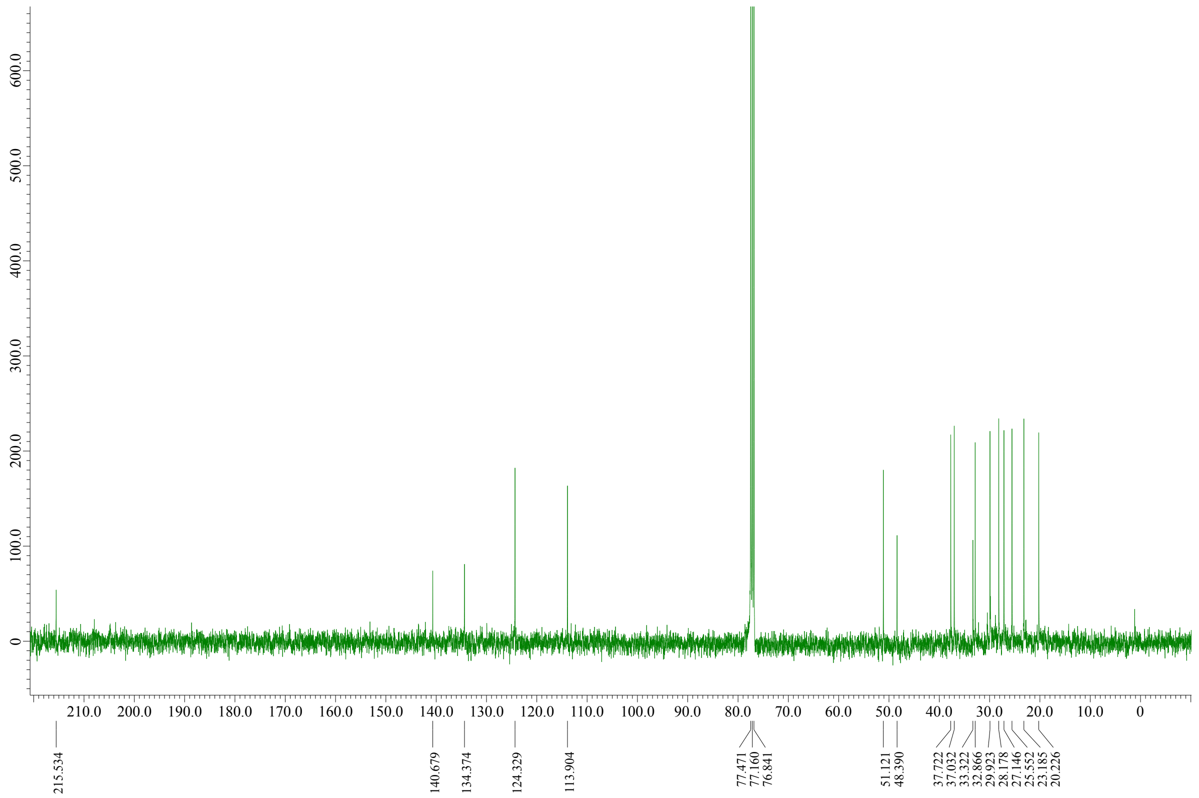


**Figure S22.** $^{1}H$ and $^{13}C$ NMR Spectra of **20** ($CDCl_3$)

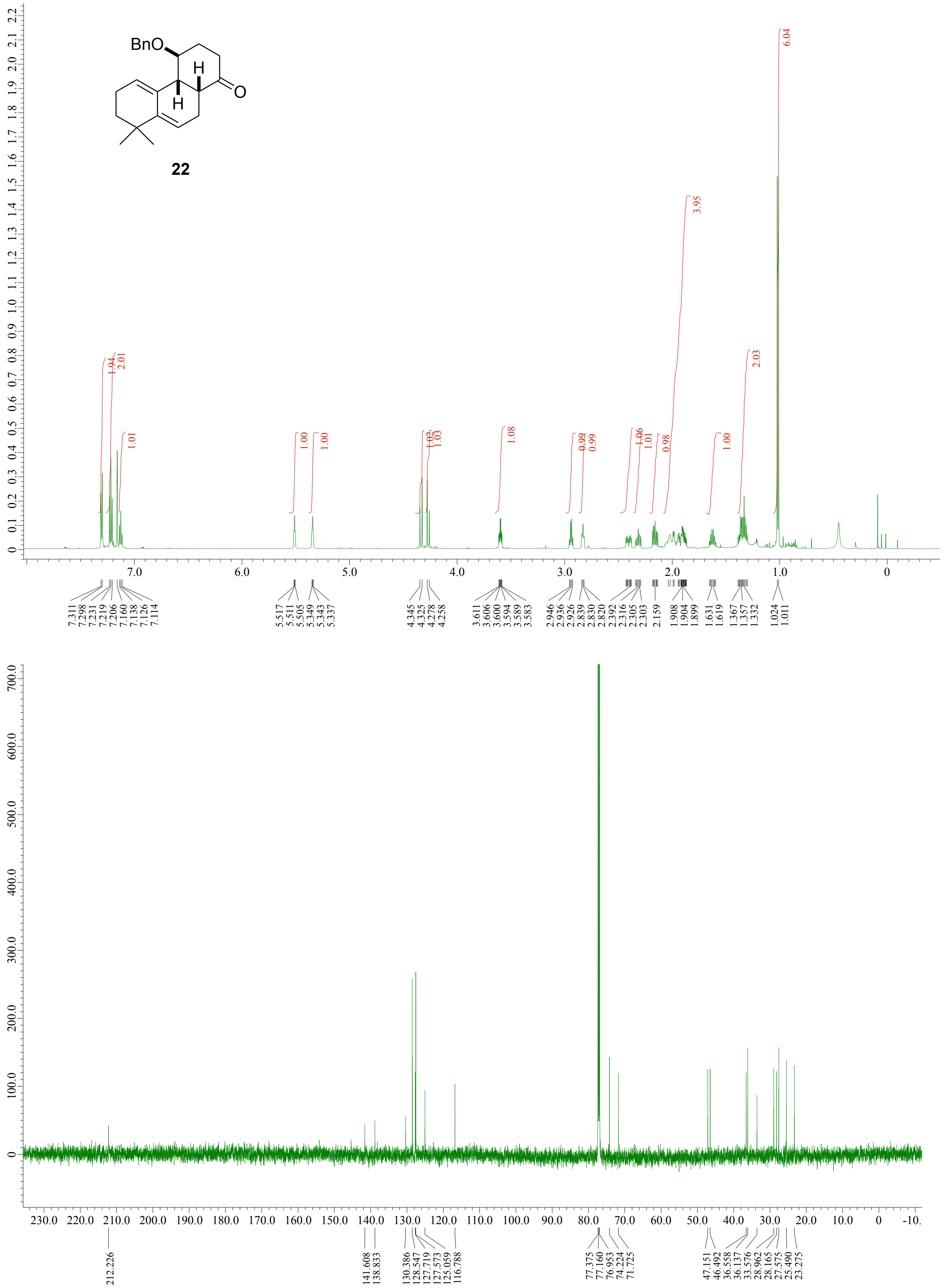

**Figure S23.** $^{1}H$ and $^{13}C$ NMR Spectra of **22** ($CDCl_3$)

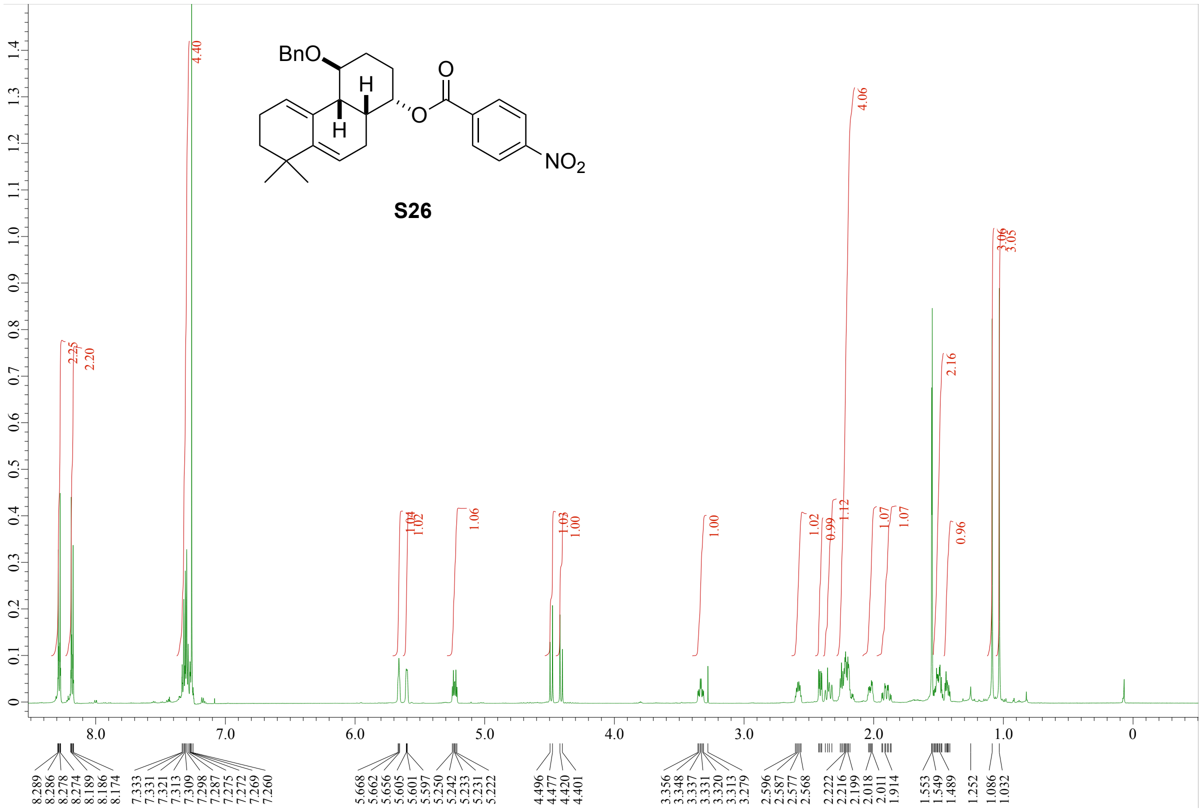

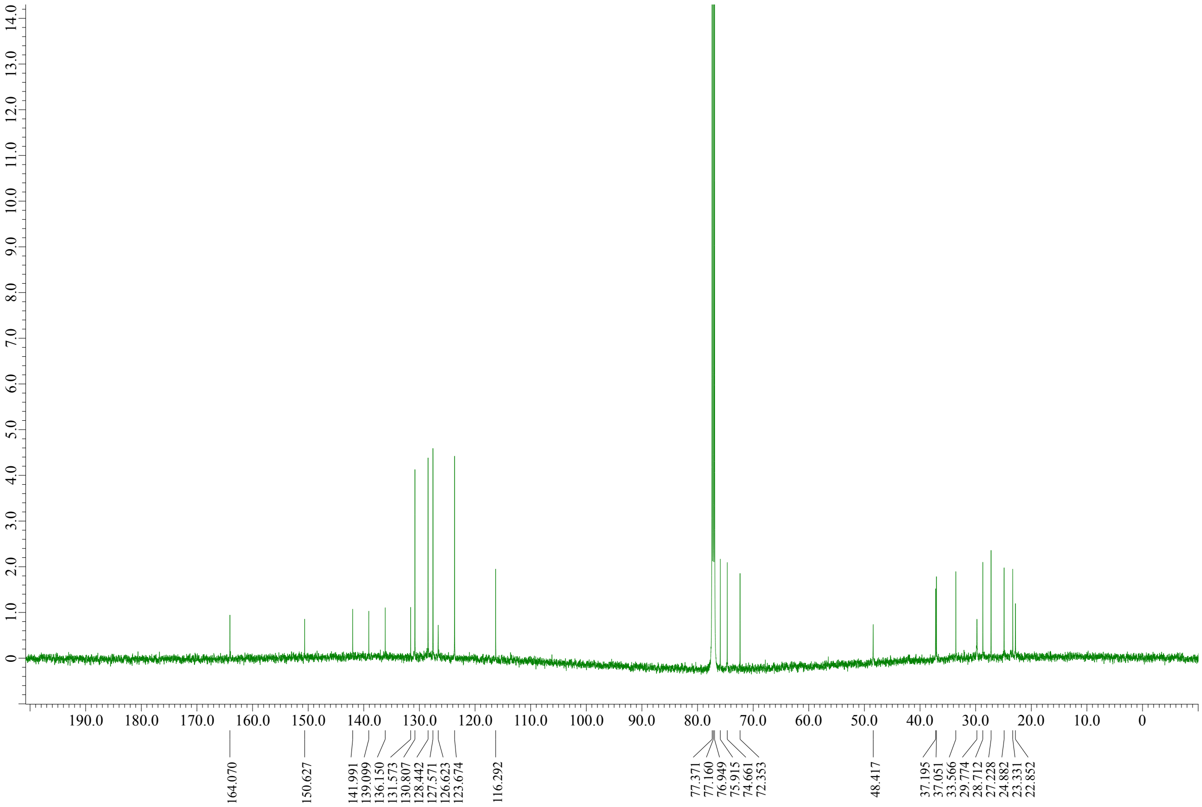

**Figure S24.** $^{1}H$ and $^{13}C$ NMR Spectra of **S26** ($CDCl_3$)

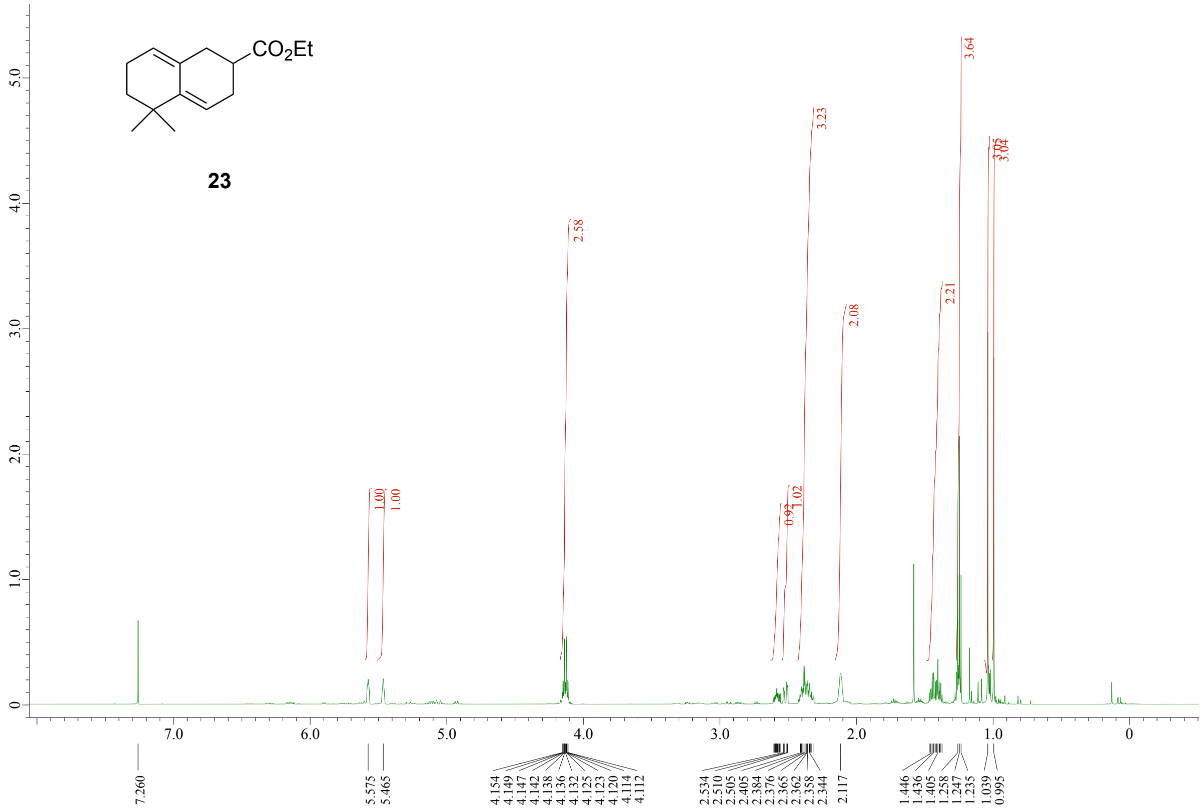


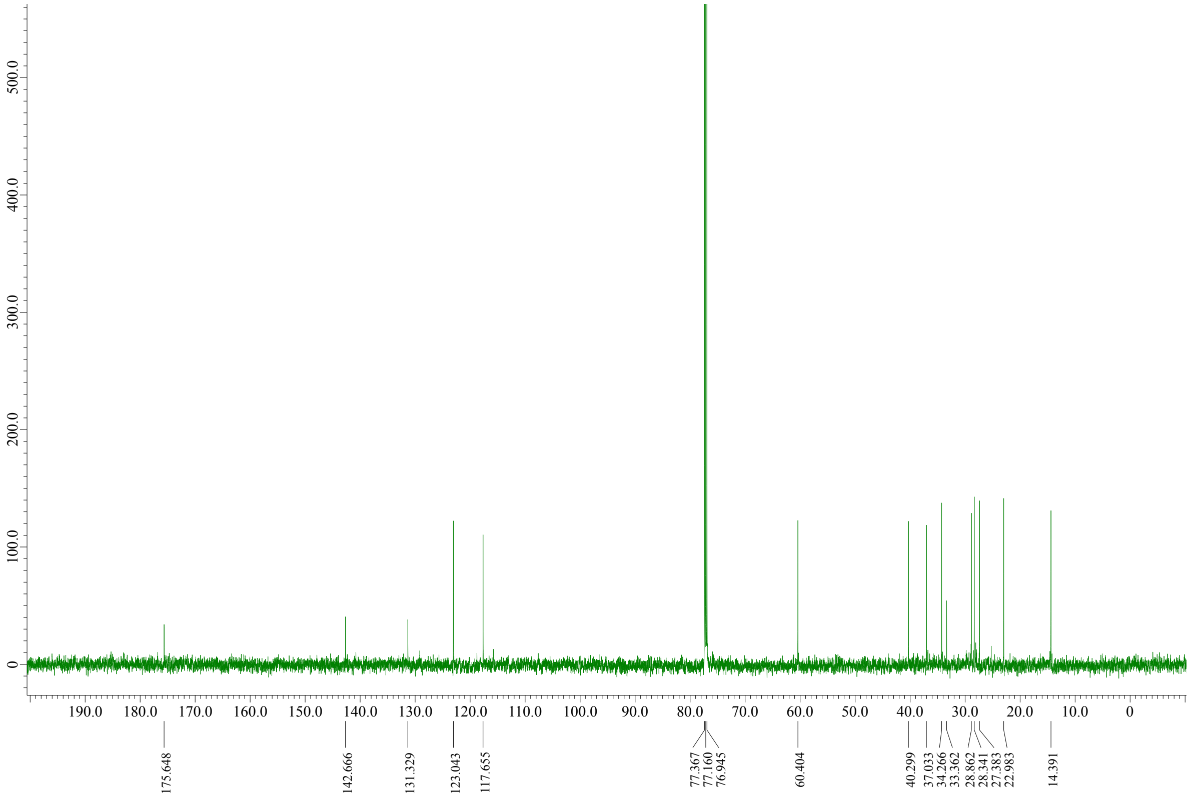


**Figure S25.** $^{1}$H and $^{13}$C NMR Spectra of **23** ($CDCl_3$)

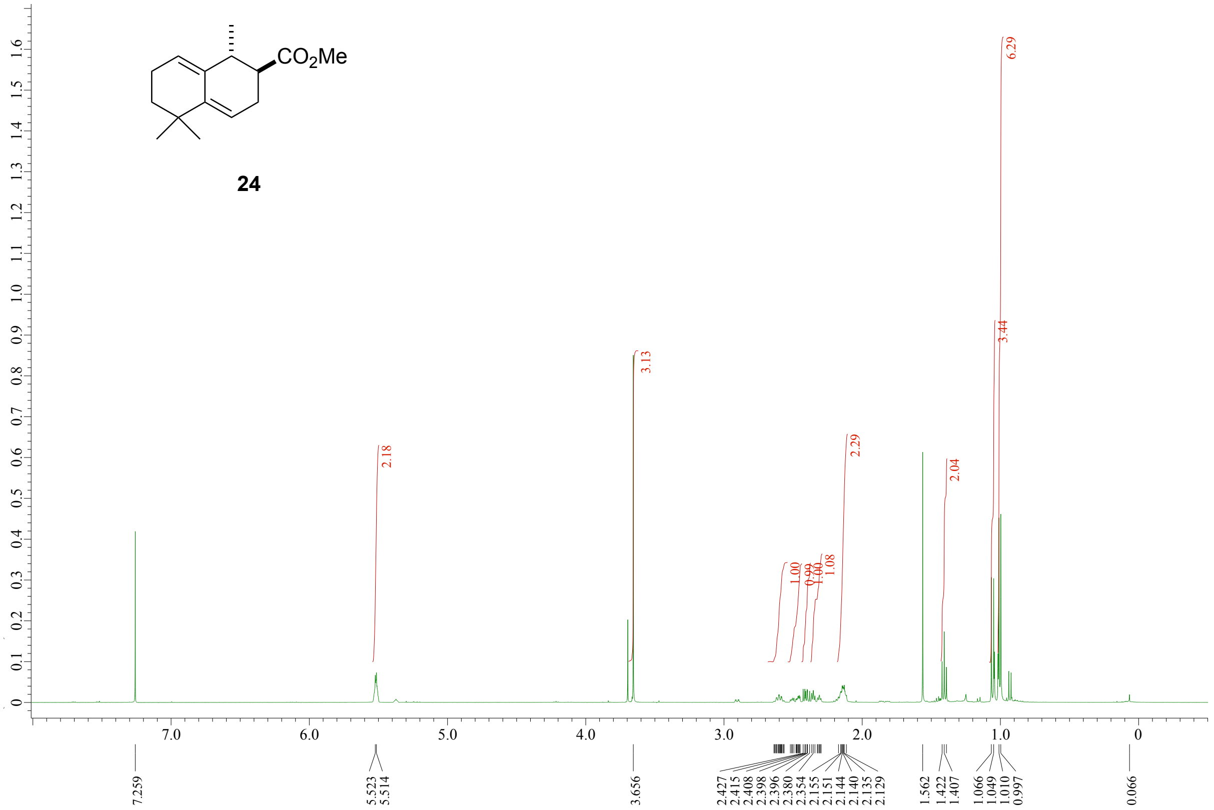


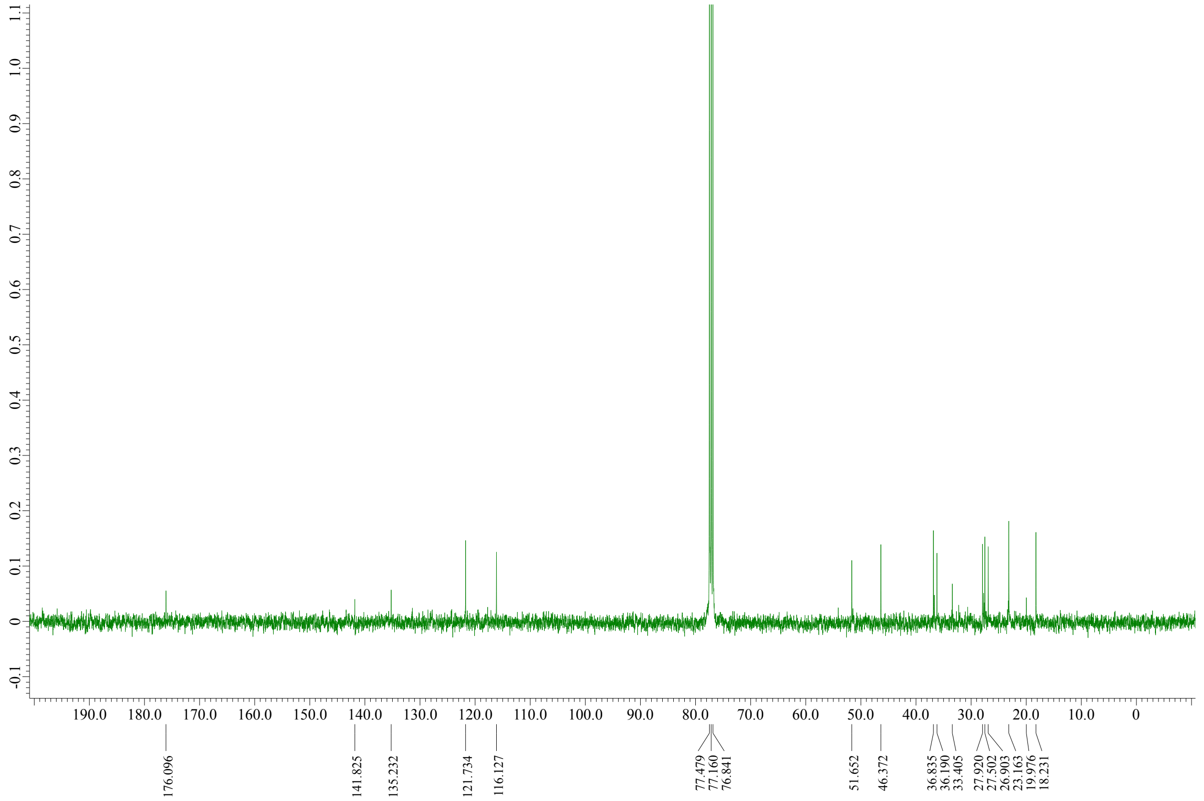


**Figure S26.** $^{1}H$ and $^{13}C$ NMR Spectra of **24** ($CDCl_3$)

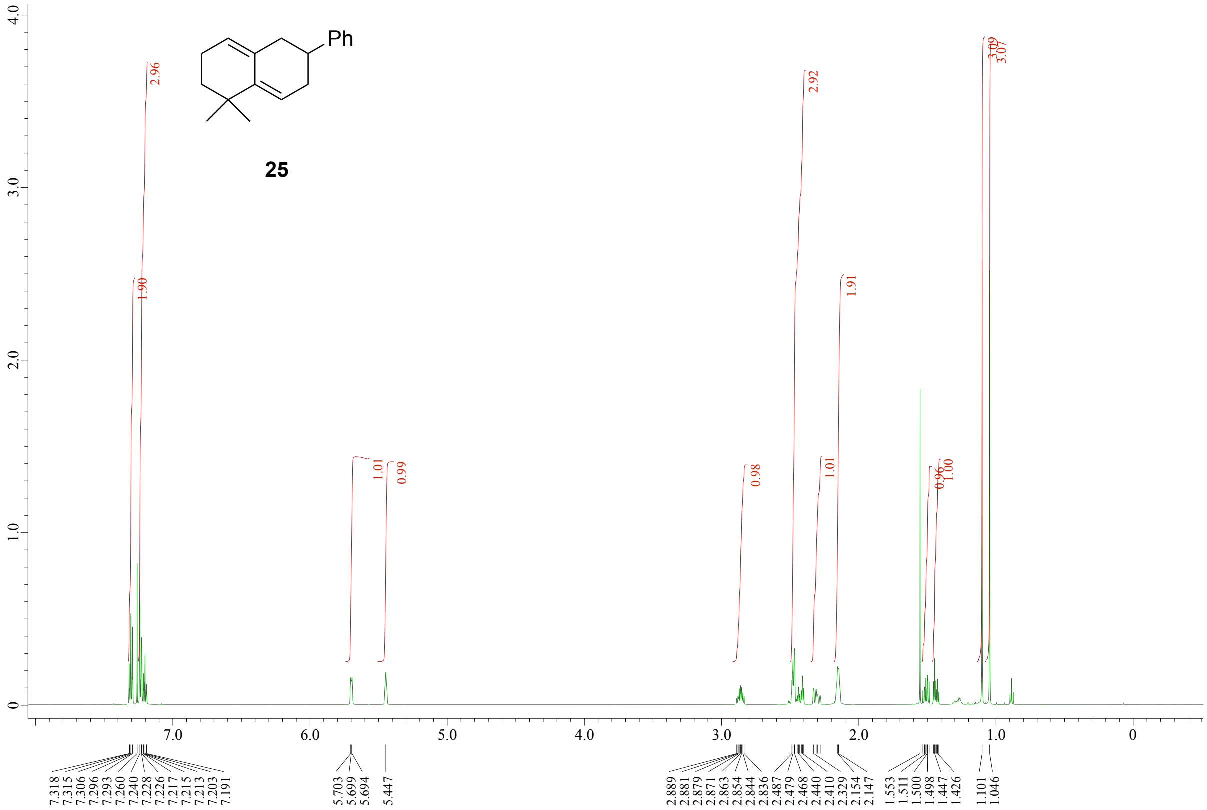

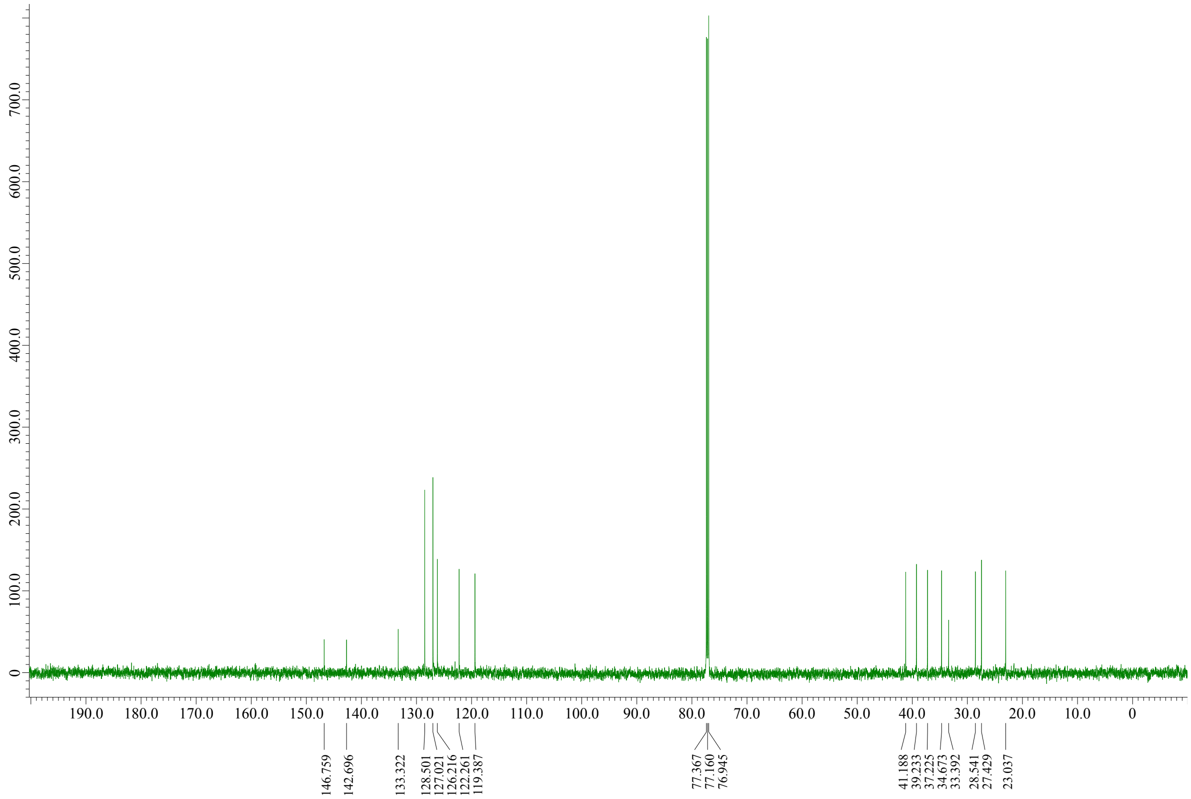

**Figure S27.** $^{1}H$ and $^{13}C$ NMR Spectra of **25** ($CDCl_3$)

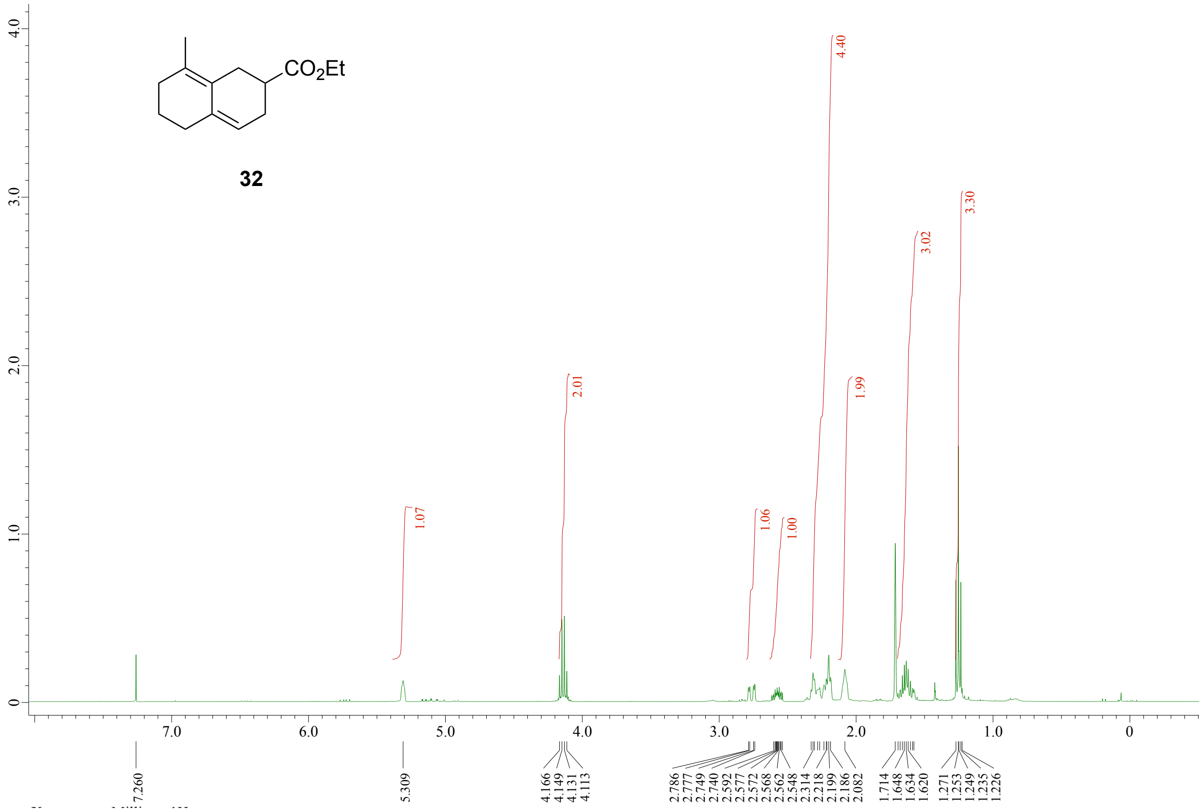

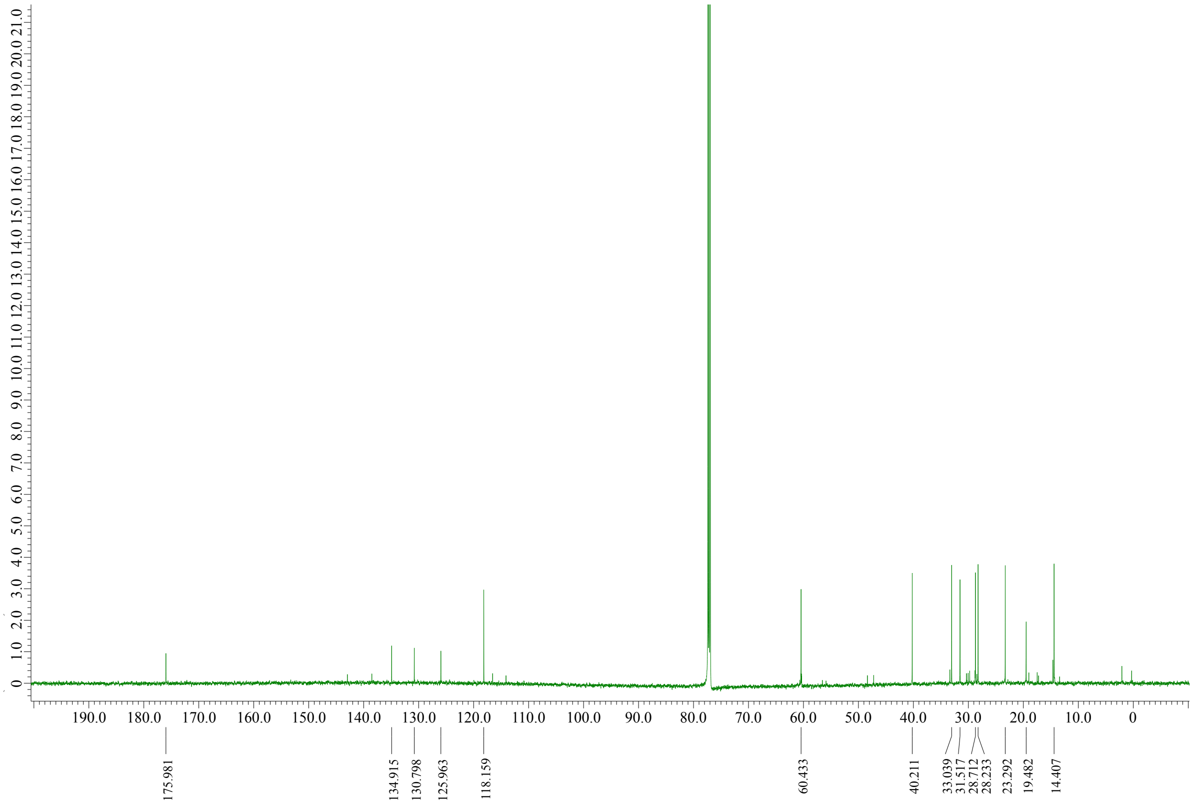

**Figure S28.** $^{1}H$ and $^{13}C$ NMR Spectra of **32** ($CDCl_3$)

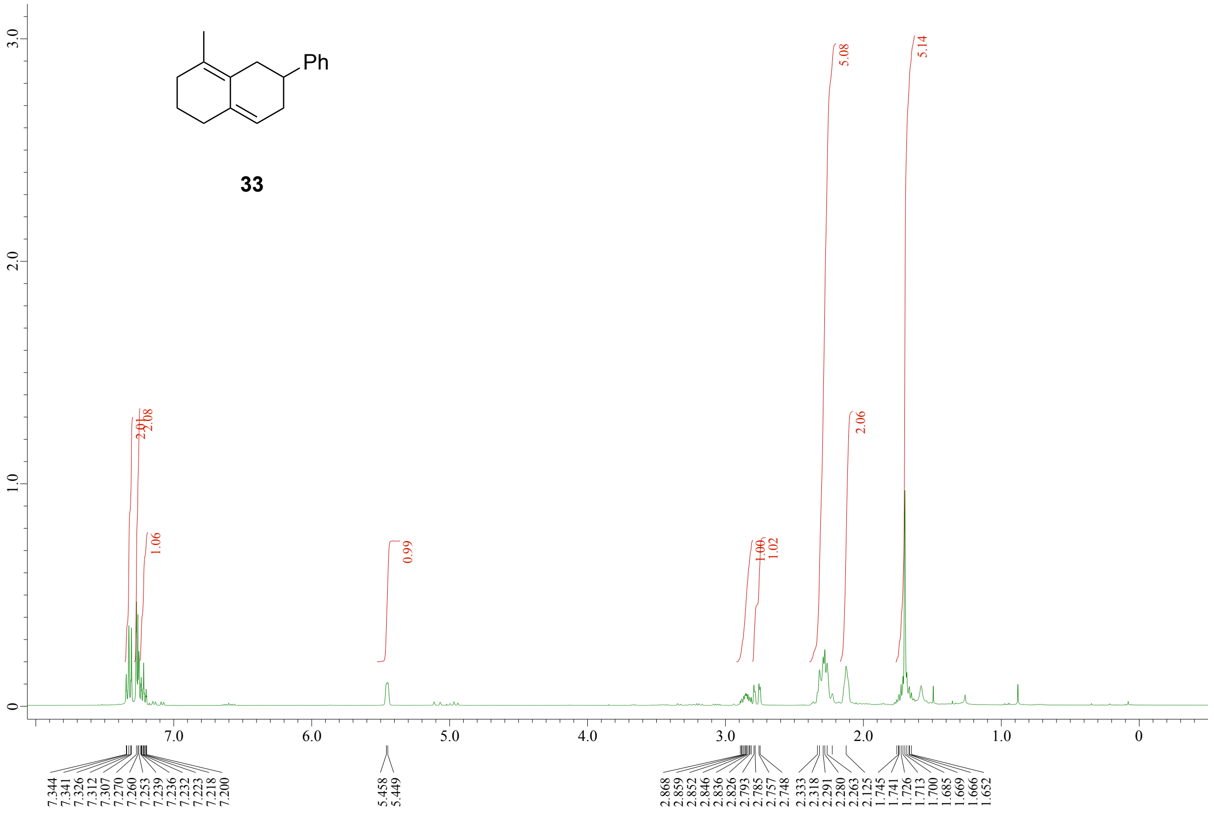


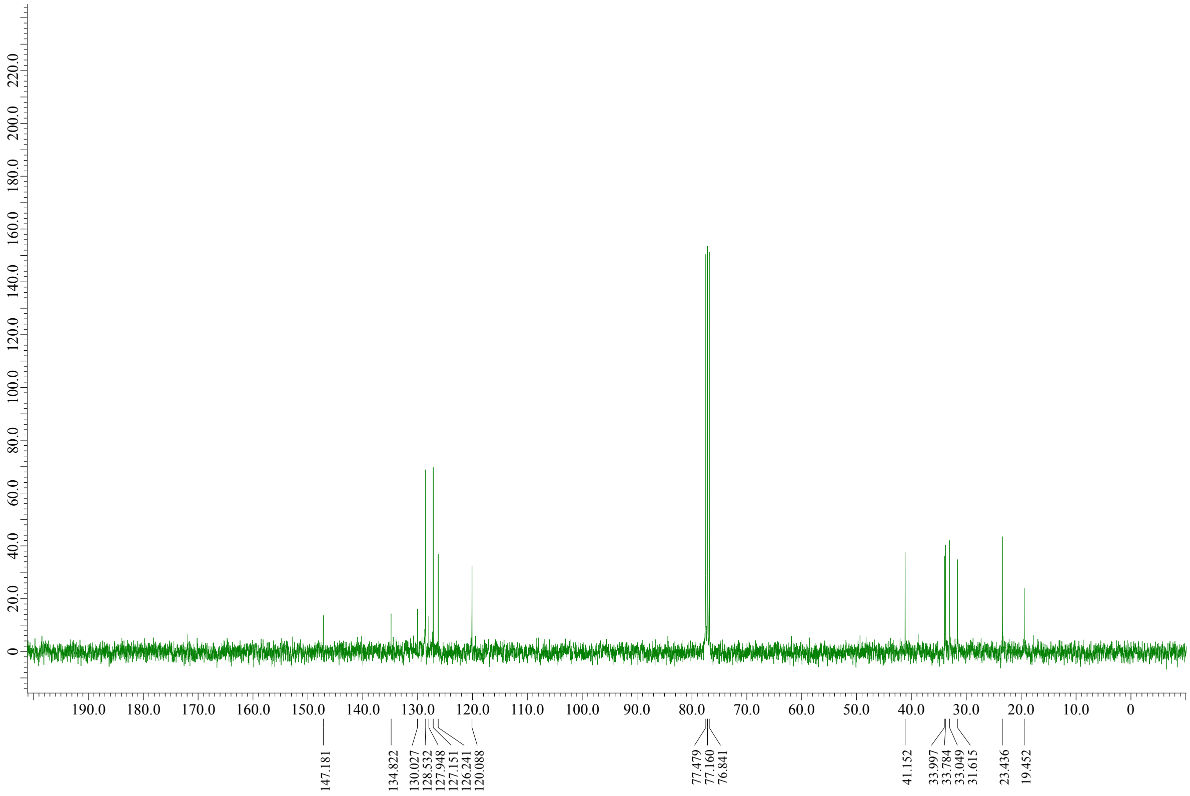

**Figure S29.** $^{1}H$ and $^{13}C$ NMR Spectra of **33** ($CDCl_3$)

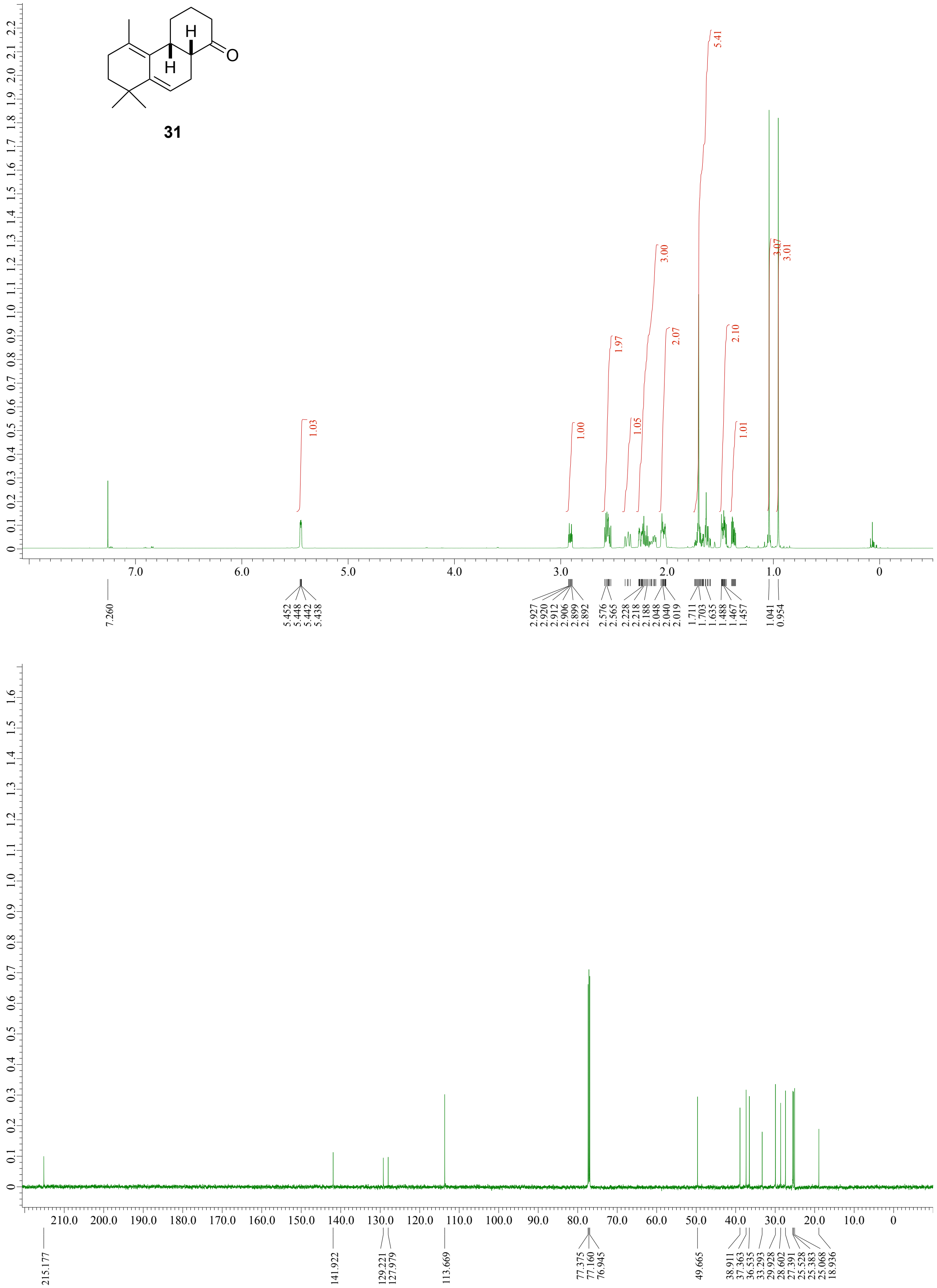


**Figure S30.** $^{1}H$ and $^{13}C$ NMR Spectra of **31** ($CDCl_3$)

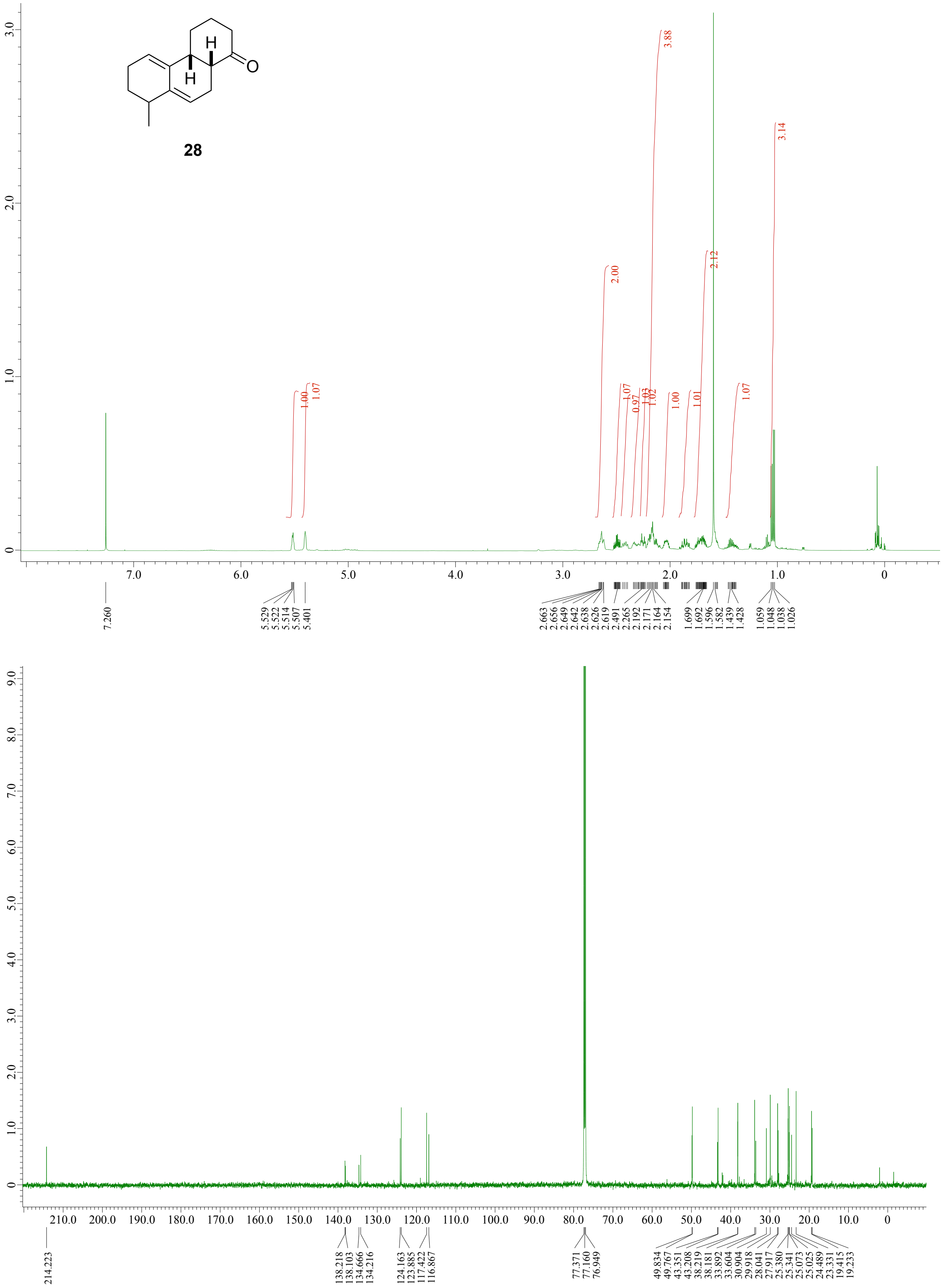

**Figure S31.** $^{1}H$ and $^{13}C$ NMR Spectra of **28** ($CDCl_3$)

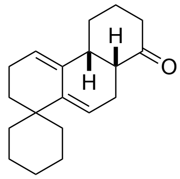

**29**

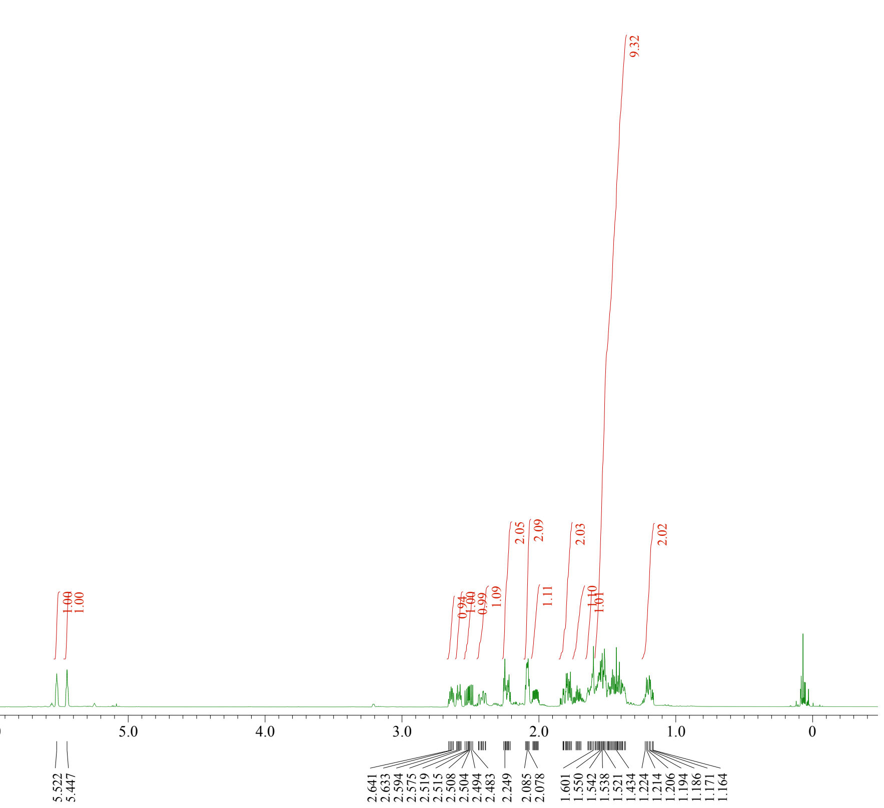

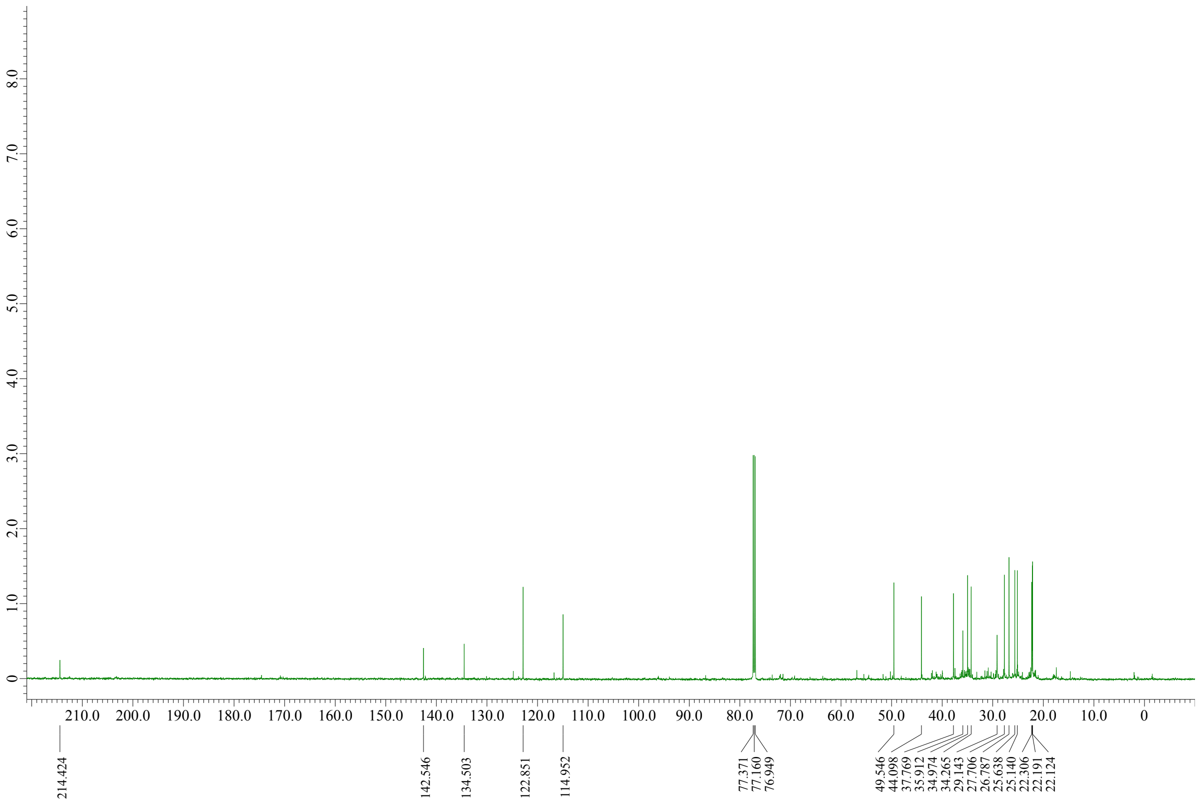

**Figure S32.** $^{1}H$ and $^{13}C$ NMR Spectra of **29** ($CDCl_3$)

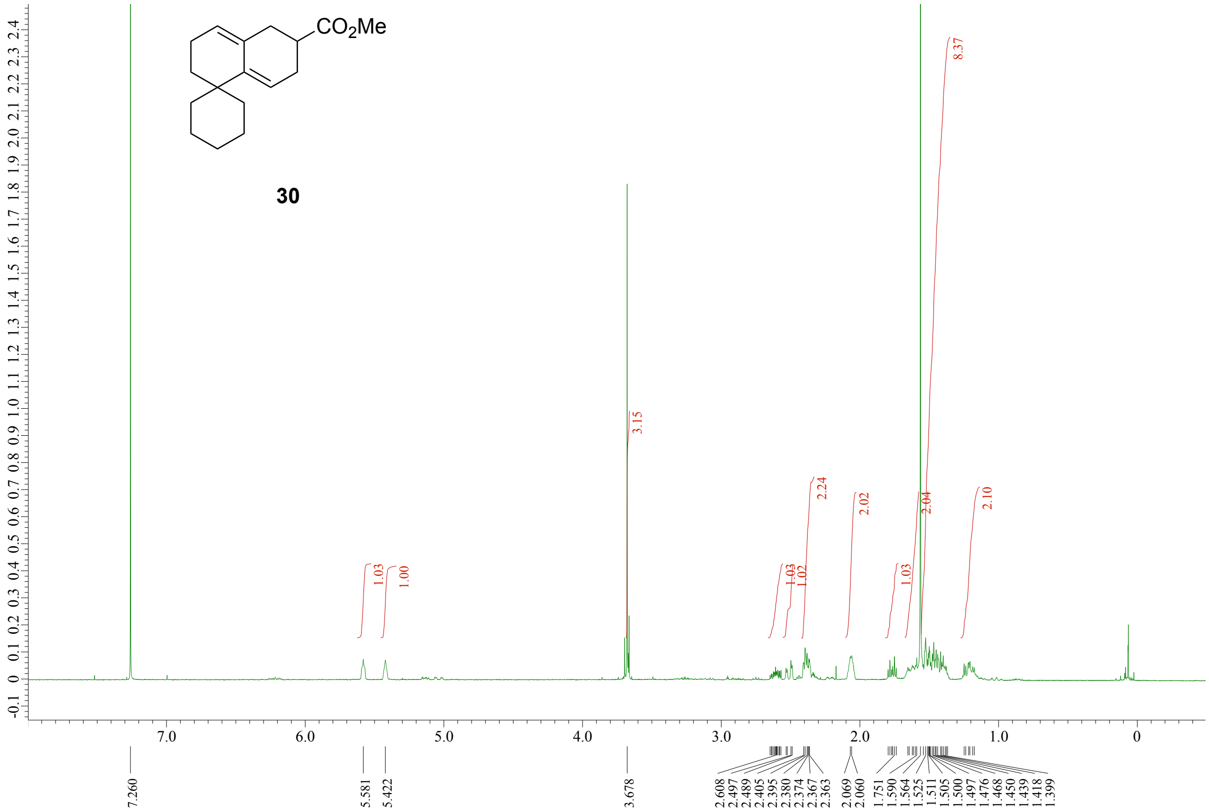

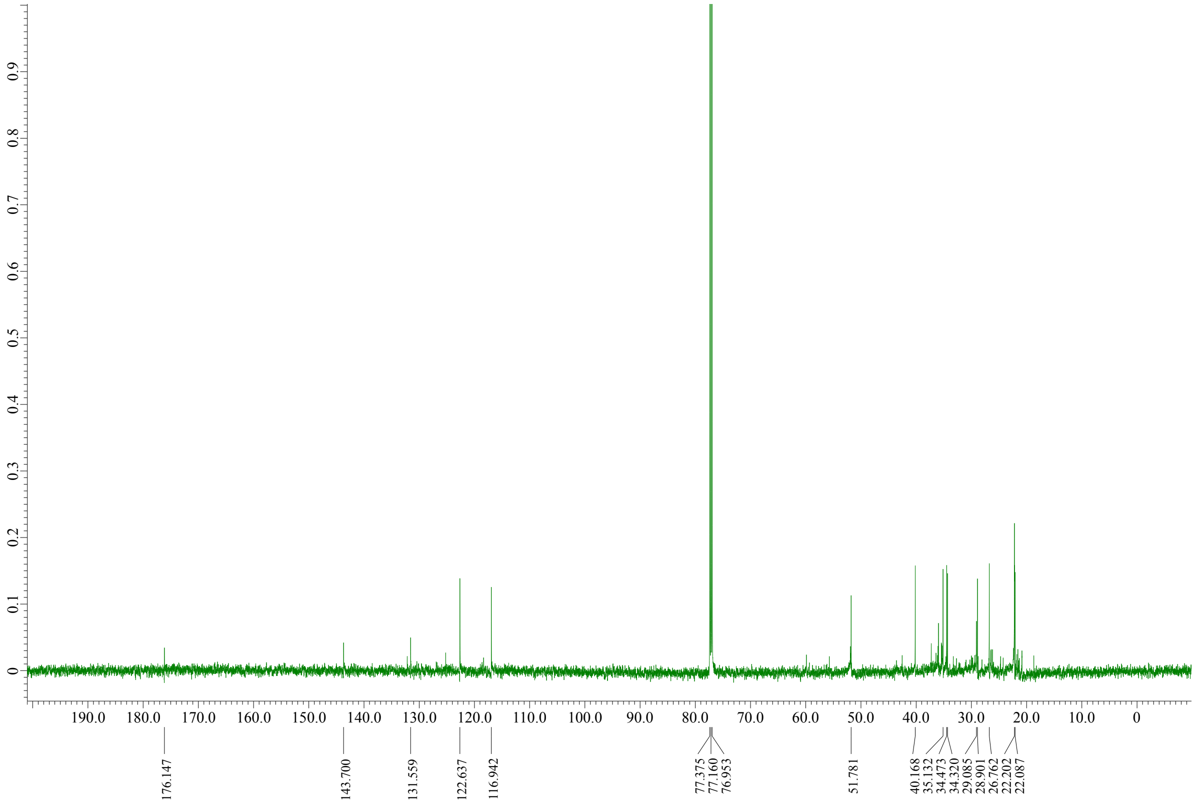

**Figure S33.** $^1$H and $^{13}$C NMR Spectra of **30** ($CDCl_3$)

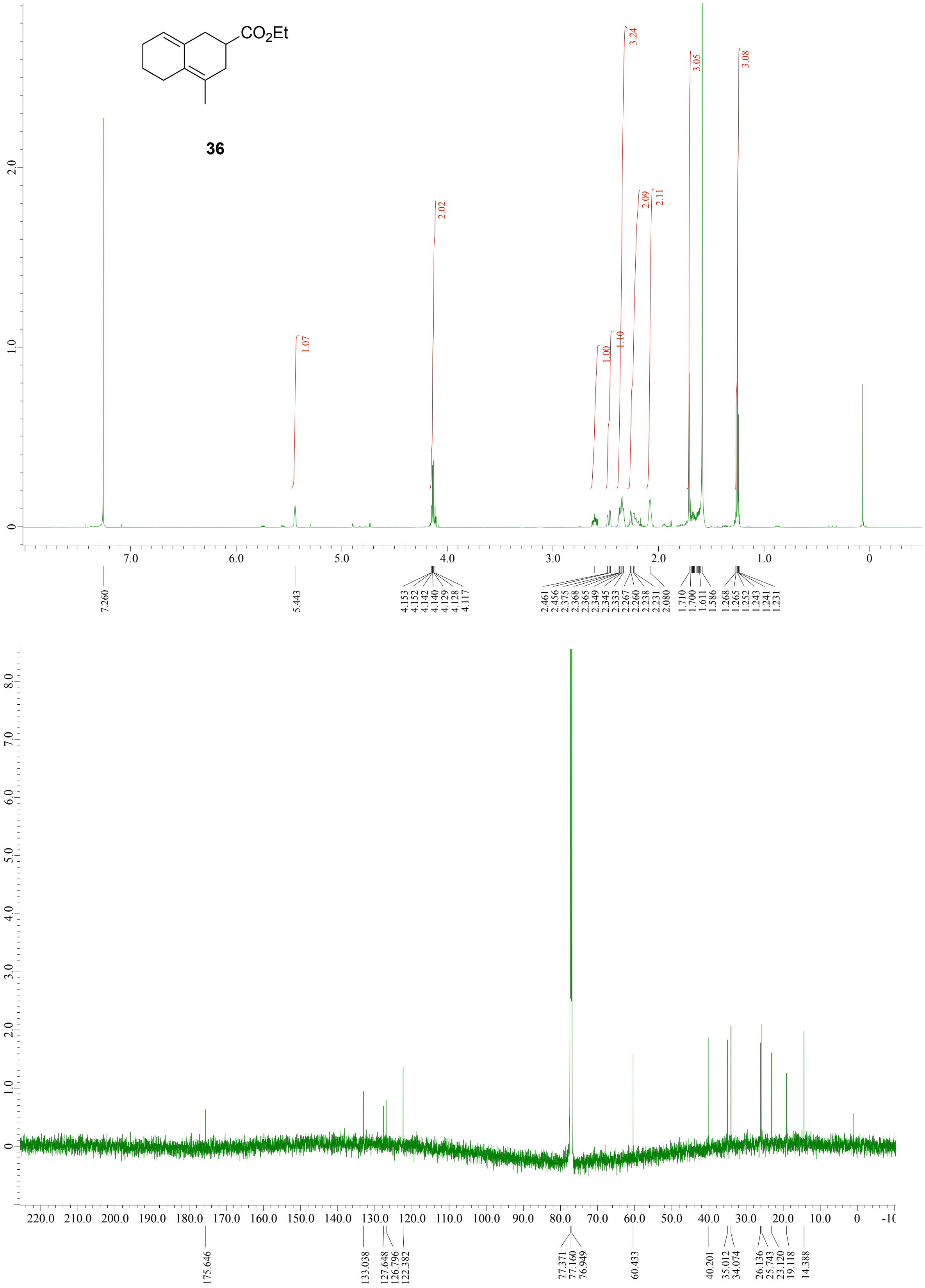


**Figure S34.** $^{1}H$ and $^{13}C$ NMR Spectra of **36** ($CDCl_3$)

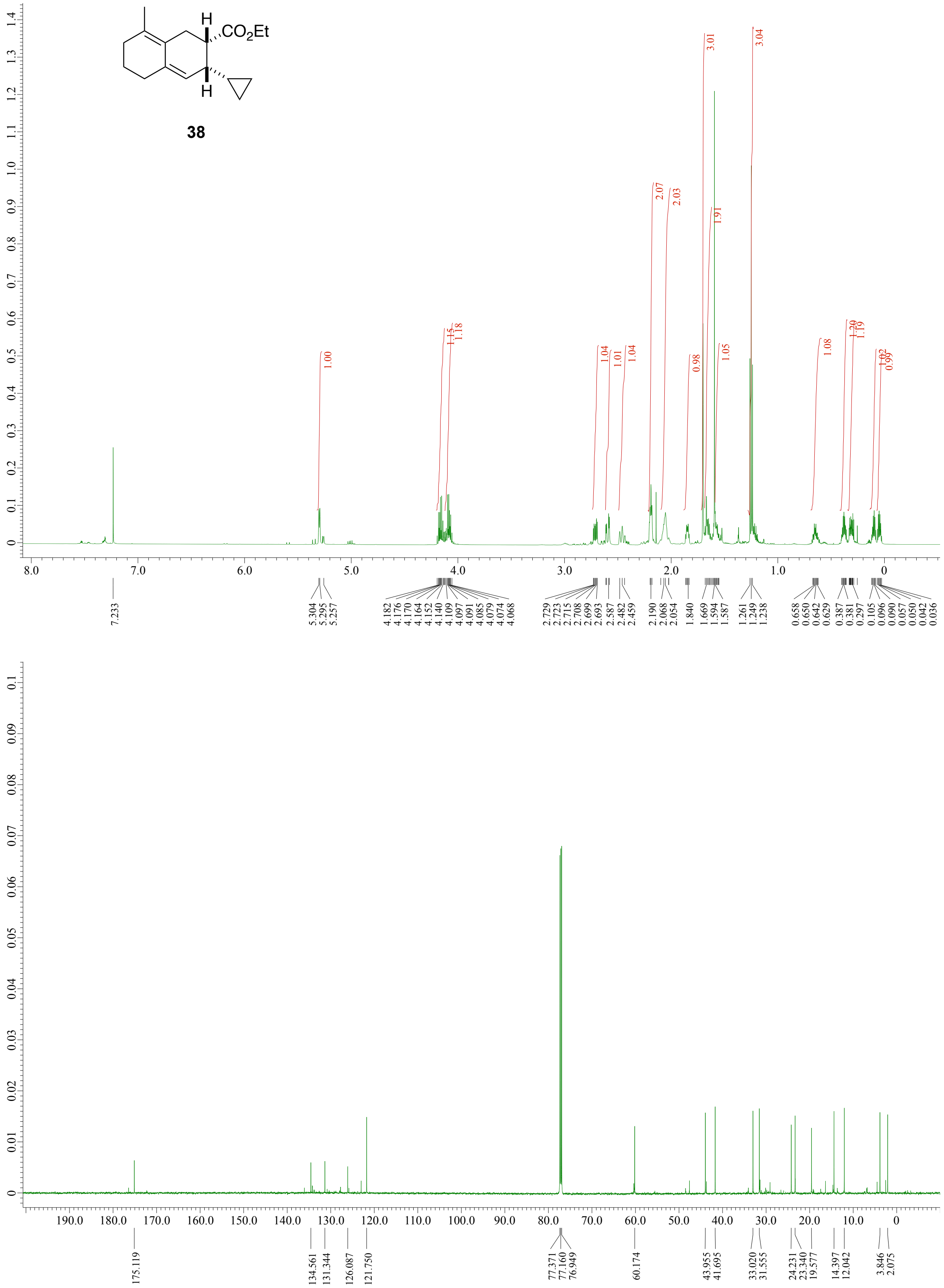


**Figure S35.** $^1$H and $^{13}$C NMR Spectra of **38** ($CDCl_3$)

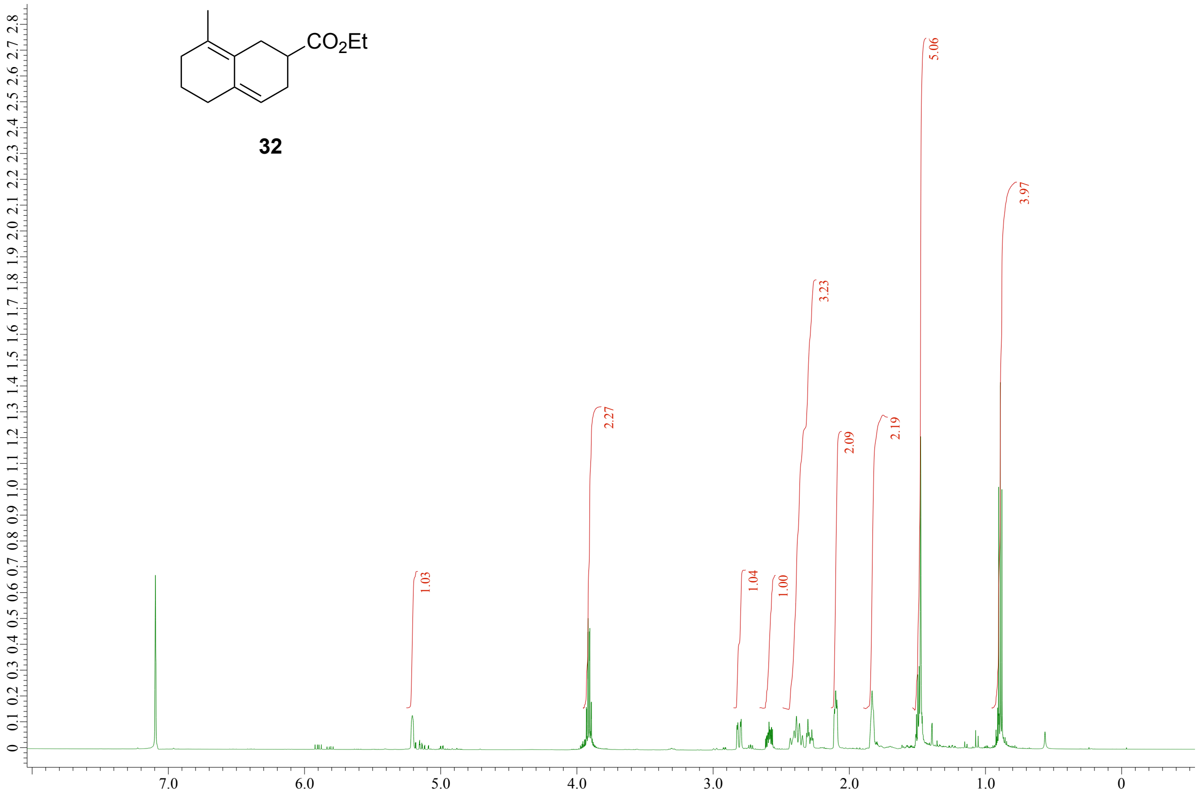


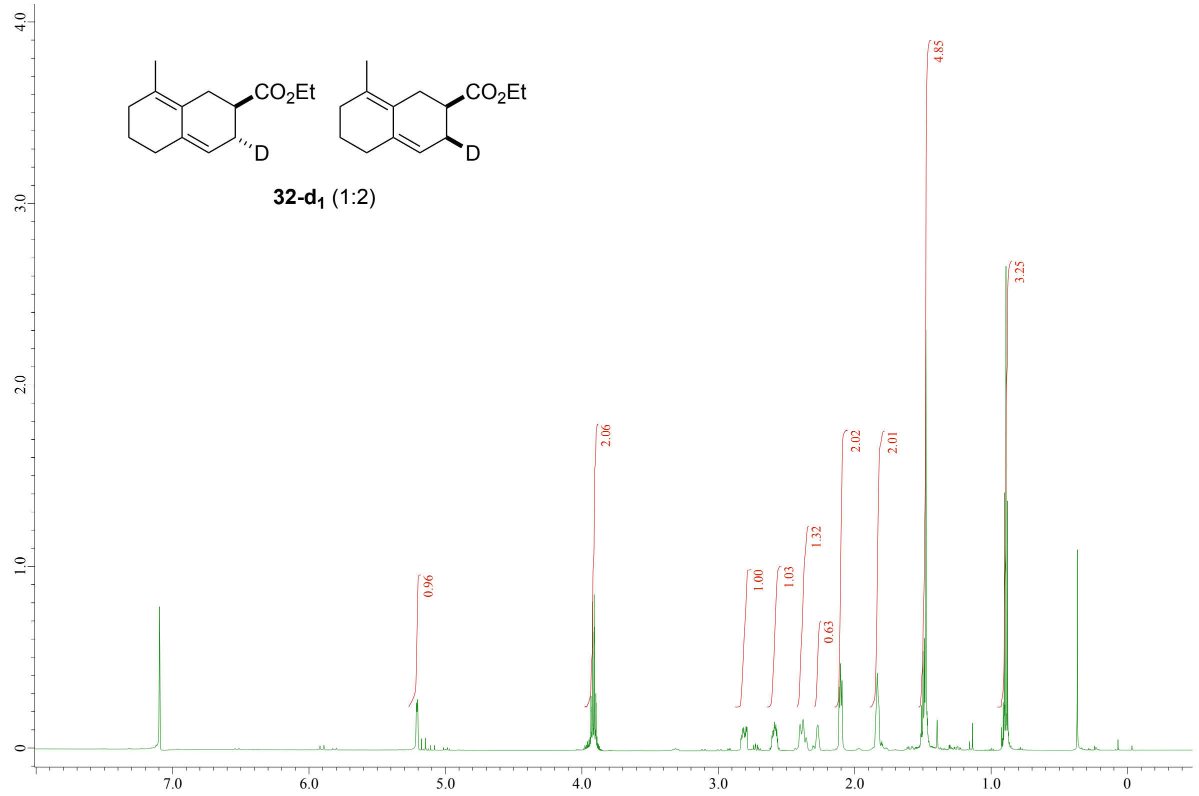


**Figure S36.** $^{1}$H NMR Spectra of **32** and **32-d$_1$** ($C_6D_6$)

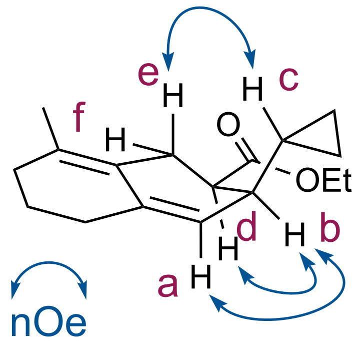


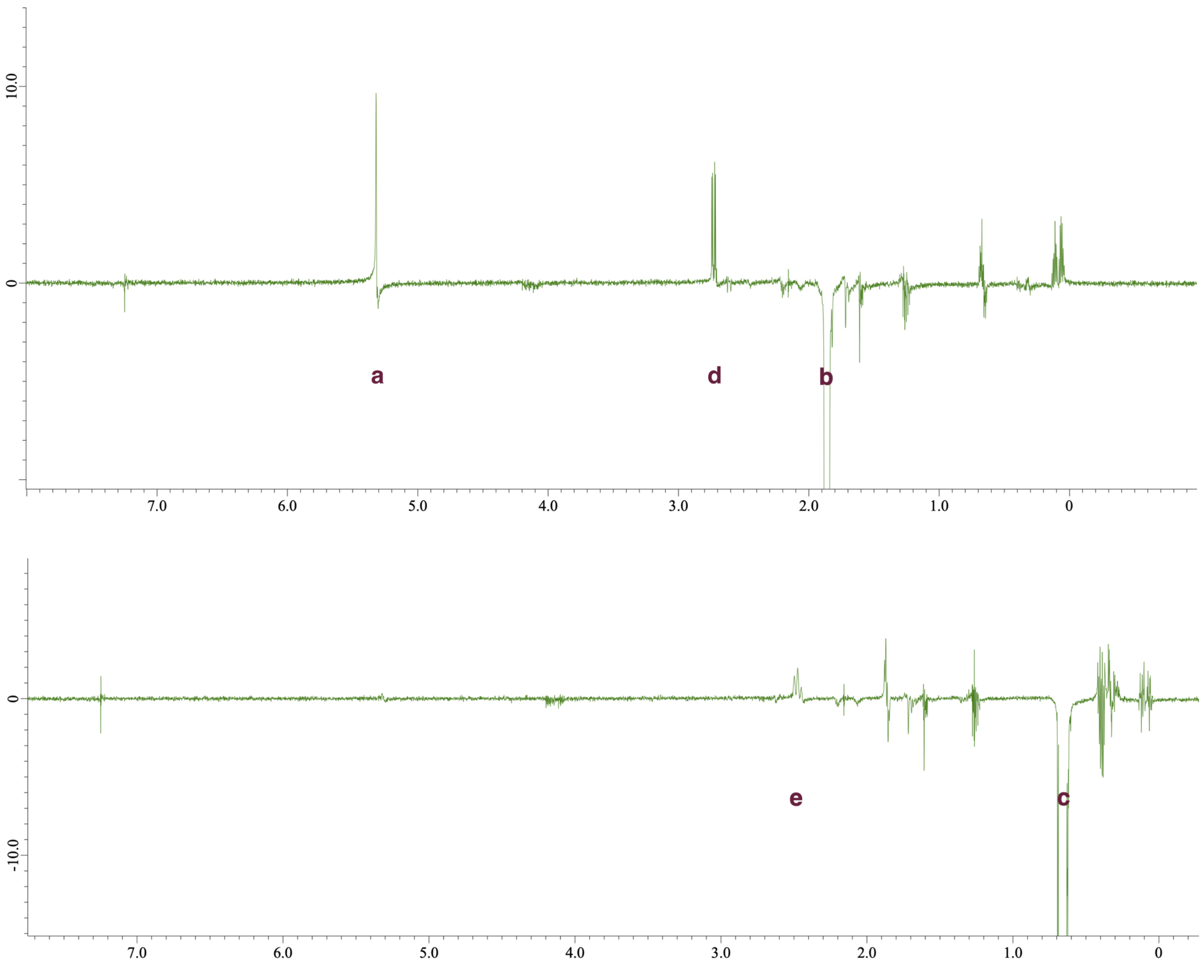


**Figure S37.** $^{1}$H NMR and 1D NOE Spectra of **38** ($CDCl_3$)

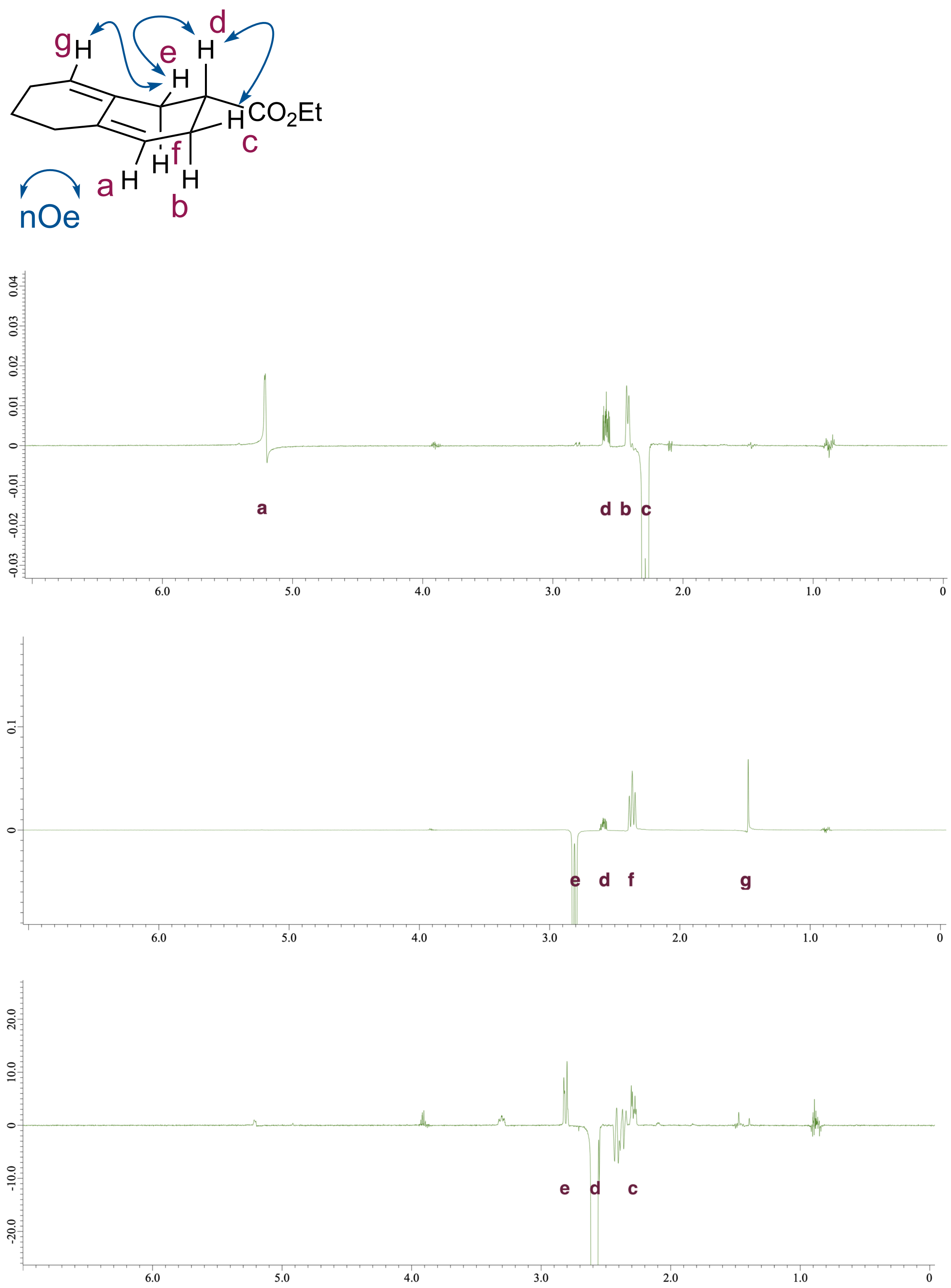


**Figure S38.** $^1$H NMR and 1D NOE Spectra of **32d** ($C_6D_6$)
